# Identification and spontaneous immune targeting of an endogenous retrovirus K envelope protein in the Indian rhesus macaque model of human disease

**DOI:** 10.1186/s12977-016-0238-0

**Published:** 2016-01-15

**Authors:** Helen L. Wu, Enrique J. Léon, Lyle T. Wallace, Francesca A. Nimiyongskul, Matthew B. Buechler, Laura P. Newman, Philip A. Castrovinci, R. Paul Johnson, Robert J. Gifford, R. Brad Jones, Jonah B. Sacha

**Affiliations:** Vaccine and Gene Therapy Institute, Oregon Health and Science University, Beaverton, OR USA; Oregon National Primate Research Center, Oregon Health and Science University, 505 NW 185th Avenue, Beaverton, OR 97007 USA; Department of Pathology and Laboratory Medicine, University of Wisconsin-Madison, Madison, WI USA; Yerkes National Primate Research Center, Emory University, Atlanta, GA USA; MRC-University of Glasgow Centre for Virus Research, Glasgow, UK; Department of Microbiology, Immunology and Tropical Medicine, George Washington University, Washington DC, USA

**Keywords:** Endogenous retroviruses (ERVs), Env proteins, Antibodies, T cells, Simian immunodeficiency virus (SIV)

## Abstract

**Background:**

Endogenous retroviruses (ERVs) are remnants of ancient retroviral infections that have invaded the germ line of both humans and non-human primates. Most ERVs are functionally crippled by deletions, mutations, and hypermethylation, leading to the view that they are inert genomic fossils. However, some ERVs can produce mRNA transcripts, functional viral proteins, and even non-infectious virus particles during certain developmental and pathological processes. While there have been reports of ERV-specific immunity associated with ERV activity in humans, adaptive immune responses to ERV-encoded gene products remain poorly defined and have not been investigated in the physiologically relevant non-human primate model of human disease.

**Findings:**

Here, we identified the rhesus macaque equivalent of the biologically active human ERV-K (HML-2), simian ERV-K (SERV-K1), which retains intact open reading frames for both Gag and Env on chromosome 12 in the macaque genome. From macaque cells we isolated a spliced mRNA product encoding SERV-K1 Env, which possesses all the structural features of a canonical, functional retroviral Envelope protein. Furthermore, we identified rare, but robust T cell responses as well as frequent antibody responses targeting SERV-K1 Env in rhesus macaques.

**Conclusions:**

These data demonstrate that SERV-K1 retains biological activity sufficient to induce cellular and humoral immune responses in rhesus macaques. As ERV-K is the youngest and most active ERV family in the human genome, the identification and characterization of the simian orthologue in rhesus macaques provides a highly relevant animal model in which to study the role of ERV-K in developmental and disease states.

**Electronic supplementary material:**

The online version of this article (doi:10.1186/s12977-016-0238-0) contains supplementary material, which is available to authorized users.

## Findings

Endogenous retroviruses (ERVs) comprise approximately 8 % of the human genome. While the vast majority of ERVs no longer possess coding capacity for the production of viral mRNAs and proteins, some ERV open reading frames (ORFs) remain intact [1, 2]. Human ERV (HERV) activity is normally repressed in healthy tissues, however, activation of HERV ORF transcription and protein production is detected in numerous developmental and pathological contexts, including embryogenesis, pregnancy, neoplasia, autoimmunity, neurodegeneration, and viral infection [3–10]. In addition, HERV-specific cellular and humoral immunity has been reported in humans, particularly in the context of exogenous retroviral infection with human immunodeficiency virus (HIV) [10–13]. However, ERV activity and ERV-specific immune responses remain poorly defined in the physiologically relevant rhesus macaque model frequently used to study human reproduction, development, neurology, and infectious disease. Therefore, we investigated here if rhesus macaques harbor a functional equivalent of HERV-K (HML-2).

### Identification of a spliced SERV-K1 Env mRNA

**Fig. 1:**
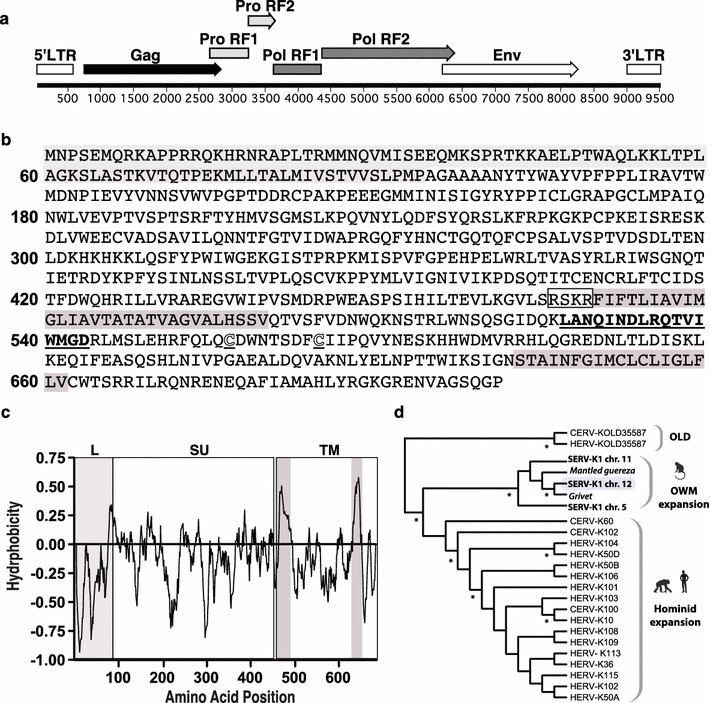
Identification of macque ERV-K provirus and a fully-spliced SERV-K1 Env mRNA. **a** Using human ERV-K sequences to search the rhesus macaque genome for ERV proviral insertions, we identified a proviral insertion of SERV-K1 on macaque chromosome 12. Genomic organization of this SERV-K1 provirus is shown. **b** Using mRNA capture, we identified a fully-spliced mRNA encoding the SERV-K1 Env protein. Predicted protein translation of the captured mRNA is shown with canonical Env structural features: leader sequence (*light grey*), R-X-K/R-R cleavage site (*boxed*), fusion domain (*grey highlight*), immunosuppressive domain (*Bolded* and *underlined*), conserved cysteine residues (*white*, *underlined* Cs), and transmembrane anchor domain (*grey highlight*). **c** Hydrophobicity plot of SERV-K1 Env protein with features highlighted as in part **b**. **d** *Phylogram* showing the relationship of the simian ERVs examined here to previously characterized HERV-K HML-2 insertions in chimpanzees and humans. The phylogram was generated using maximum likelihood as implemented in PHYML [27] with 1000 bootstrap replicates. The tree is rooted on HERV-K(OLD) proviruses previously determined to be ancestors of the HML-2 lineage in Old World primates [28]. The macaque locus examined here is indicated by *background shading*. *Asterisks* indicate nodes that were recovered in >95 % of bootstrap trees. *OWM* old world monkey, *Chr* chromosome

To assess whether a biologically active ERV might exist in the genome of Indian rhesus macaques, we focused on HERV-K (HML-2), the youngest and most active of all ERVs in the human genome [14]. To this end, we used consensus HERV-K sequences to scan the rhesus macaque genome, where we identified three proviral insertion sites on chromosomes 5, 11, and 12. The proviral insertion on chromosome 12 exhibited the greatest sequence homology to HERV-K, with 89 % nucleotide homology to HERV-K Gag, Pro, Pol, and Env open reading frames; we termed this provirus simian ERV-K1 (SERV-K1) (Additional file 1: Figure S1). While the Pro and Pol reading frames contained frameshift mutations leading to premature stop codons, the Gag and Env ORFs remained intact with the potential to code for full length proteins (Fig. 1a). To assess whether the SERV-K1 provirus could produce protein-coding mRNA transcripts, we used mRNA capture to generate a cDNA library from a macaque in which we detected a large SERV-K1 Env T cell response (r02120, see below). The cDNA library from r02120 contained a spliced mRNA transcript coding for the SERV-K1 Env protein possessing canonical structural features of a retroviral Env protein, including a signal leader peptide (L), a RX(K/R)R consensus cleavage site separating the surface (SU) and transmembrane (TM) envelope subunits, a fusion domain, an immunosuppressive domain, and a transmembrane anchor domain (Fig. 1b, c; Additional file 2: Figure S2). Phylogenetic analysis confirmed that SERV-K1 is part of a simian ‘sister’ lineage of the HERV-K (HML-2) lineage that expanded in the hominid germline (Fig. 1d; Additional file 3: Figure S3). As HERV-K HML2 Env and SERV-K1 Env share >90 % sequence homology, it is likely that, as described for HERV-K Env in humans [14], SERV-K1 Env retains functional activity in rhesus macaques, and thus may serve as a target for immune responses.

### SERV-K1 Env-specific T cell responses are rare, but can be high frequency

**Fig. 2:**
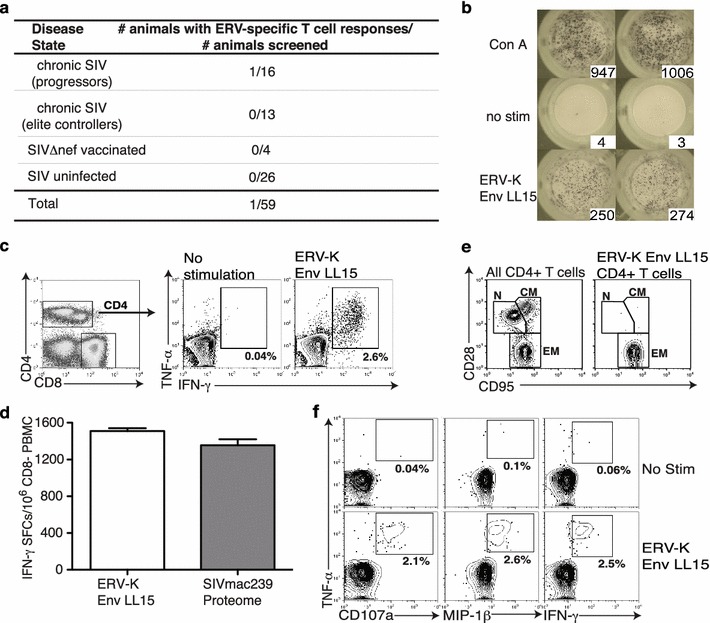
SERV-K1 Env-specific T cell responses are rare, but can be high frequency. **a** Fifty-nine Indian rhesus macaques were screened for SERV-K1 Env T cell responses using overlapping 15-mer peptides spanning SERV-K1 Env in IFN-γ ELISPOT. *Table* shows summarized results. **b** Raw IFN-γ ELISPOT images of SERV-K1 Env LL15-specific T cell response identified in r02120 performed in duplicate alongside positive control (Concanavalin A, Con A) and negative control (no stimulation, no stim) wells. **c** Intracellular cytokine staining (ICS) flow plots showing TNF-α and IFN-γ cytokine induction in SERV-K1 Env LL15-responding T cells. Gating scheme shown on left displays the CD3+ cell population, demonstrating that the SERV-K1 Env LL15-specific T cell response is CD4+ T cell-mediated. **d** ELISPOT was performed on CD8-depleted PBMC from r02120 stimulating with either SERV-K1 Env LL15 peptide, or a pool of overlapping 15-mer peptides spanning the entire SIVmac239 proteome. *Graphs* display summed responses performed in duplicate. SFCs = IFN-γ spot forming cells. **e** Flow plots display memory subset staining of either bulk CD4+ T cells (*left*) or SERV-K1 Env LL15-responding CD4+ T cells (*right*, gated by TNF-α/IFN-γ induction shown in part **c**). Memory subsets are labeled as follows: *N* naïve, *CM* central memory, *EM* effector memory. Note SERV-K1 Env LL15-specific T cells are uniformly effector memory in phenotype. **f** ICS *flow plots* showing functionality of SERV-K1 Env LL15-specific CD4+ T cells based on induction of TNF-α in conjuction with IFN-γ, MIP-1β, and CD107a staining. Plots are gated on CD3+CD4+ as in part **c**. Note frequency of response of double positive cells is similar among plots, suggesting LL15-specific CD4+ T cells are polyfunctional. Of interest, screening of two half-siblings of r02120’s dam and four siblings of r02120’s sire revealed no SERV-K1 Env-specific T cell responses

Because SERV-K1 Env retained the ability to code for functional protein, we next assessed whether this activity in rhesus macaques was sufficient to trigger SERV-K1 Env-specific immune responses. To this end, we generated a set of overlapping 15-mer peptides spanning the SERV-K1 Env protein sequence and performed IFN-γ ELISPOT using PBMC from both SIV-infected and -uninfected rhesus macaques. We selected SIV-infected macaques due to the well-documented association between HIV-1 infection and increased HERV activity [15]. Although we screened 29 chronically SIV-infected rhesus macaques (16 progressors and 13 elite controllers), 4 SIVΔNef vaccinated rhesus macaques, and 26 SIV-naïve rhesus macaques we identified only one rhesus macaque mounting T cell responses against SERV-K1 Env (Fig. 2a). As mentioned above, this SIV-infected progressor, r02120, was the same macaque from which we had captured the SERV-K1 Env-encoding mRNA, providing further evidence that SERV-K1 was transcriptionally active in this animal.

Although SERV-K1 Env-specific T cells were only rarely detected in our cohort of macaques via IFN-γ ELISPOT, we identified one response in r02120 targeting SERV-K1 Env_526–540_ LL15 with surprisingly high magnitude (Fig. 2b). Given that impurities in commercially prepared peptides can result in false positive responses in IFN-γ ELISPOT [16, 17], we confirmed this high magnitude T cell response using SERV-K1 Env_526–540_ LL15 peptide synthesized by three independent sources (data not shown). Intracellular cytokine staining (ICS) revealed that this high frequency response was CD4+ T cell mediated (Fig. 2c). Strikingly, the SERV-K1 Env_526–540_ LL15-specific CD4+ T cell response was larger in magnitude than the entire SIVmac239-specific CD4+ T cell response in r02120 (Fig. 2d), and could be detected in ELISPOT at concentrations of 1 μM (Additional file 4: Figure S4). Further analysis of this unusual CD4+ T cell response revealed that SERV-K1 Env_526–540_ LL15-specific CD4+ T cells were uniformly effector memory in character (Fig. 2e), suggesting continual exposure to low levels of antigen. SERV-K1 Env_526–540_ LL15-specific CD4 + T cells were also highly polyfunctional, as evidenced by secretion of cytokines (TNF-α and IFN-γ) and chemokine (MIP-1β) and degranulation (measured by CD107a) (Fig. 2f). The effector memory phenotype coupled with the high frequency of responding T cells is reminiscent of the phenomenon of “memory inflation”, which is seen with chronic pathogens such as herpes viruses [18].

**Fig. 3:**
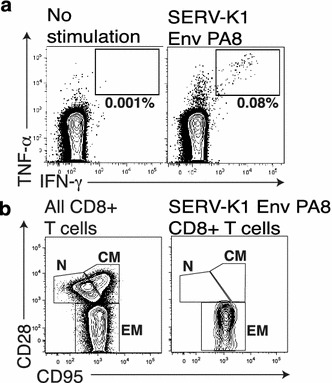
SERV-K1 Env-specific CD8+ T cell response. **a** ICS *flow plots* showing TNF-α and IFN-γ cytokine induction in SERV-K1 Env PA8-responding T cells. **b** Flow plots display memory subset staining of either bulk CD8+ T cells (*left*) or SERV-K1 Env PA8-responding CD8+ T cells (*right*, gated by TNF-α/IFN-γ induction shown in part **a**). Memory subsets are labeled as follows: *N* naïve, *CM* central memory, *EM* effector memory. Note SERV-K1 Env PA8-specific T cells are uniformly effector memory in phenotype

In addition to the large SERV-K1 Env_526–540_ LL15-specific CD4+ T cell response, r02120 also mounted a CD8+ T cell response to SERV-K1 Env_108–115_ PA8 (Fig. 3a). Similar to the CD4+ T cell response, SERV-K1 Env_108–115_ PA8-specific CD8+ T cells could be detected in IFN-γ ELISPOT at concentrations of 100 nM (Additional file 4: Figure S4) and were uniformly effector memory in phenotype (Fig. 3b). Thus, while SERV-K1 Env-directed T cell responses are not frequent in SIV-infected rhesus macaques, rare, high frequency, responses can be detected, suggesting that SERV-K1 is active in at least a subset of these animals.

### SERV-K1 Env-specific antibodies are common in rhesus macaques

**Fig. 4:**
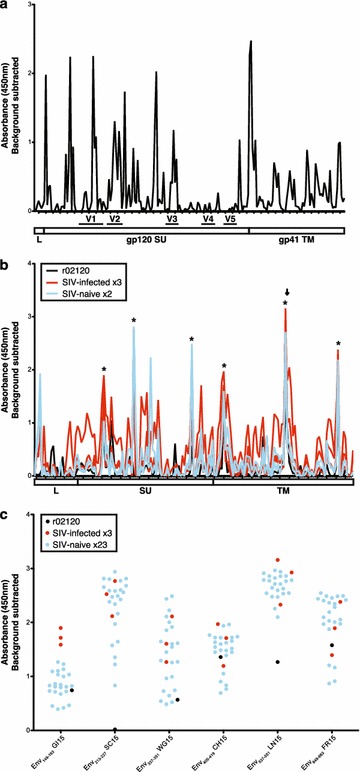
SERV-K1 Env-specific antibodies are common in rhesus macaques. **a** Overlapping 15-mer peptides spanning SIVmac239 Env were used in a peptide-based ELISA for detection of specific antibodies in the serum of r02120. **b** Overlapping 15-mer peptides spanning SERV-K1 Env were used in a peptide-based ELISA for detection of specific antibodies in the serum of 02120 (*black*) as well as the plasma of 3 additional SIV-infected (*red*) and 2 SIV-naïve RMs (*blue*). *Arrow* indicates SERV-K1 Env_541–555_ 15-mer, which is an epitope targeted in HIV-infected patients. Peptides indicated with *asterisks* (*) are shown in part **c**. *Graphs* in **a**, **b** indicate the major subunits and variable loops (V1–V5) of the Env proteins. **c** ELISA detection of antibody responses to six frequently-targeted SERV-K1 15-mer peptides in plasma from 21 SIV-naive rhesus macaques (*blue*). SIV-infected rhesus macaques from part **b** are shown for reference. All *graphs* display the average of two duplicate wells, background subtracted

As we had captured an mRNA encoding SERV-K1 Env with all the structural elements of a functional envelope protein, and detected both CD8+ and CD4+ T cells targeting this protein in r02120, we asked whether SERV-K1 Env-specific antibody responses would be present in this animal. To assess this, and to identify the immunogenic domains of SERV-K1 Env, we performed a peptide-based ELISA assay to screen for antibodies directed to SIVmac239 Env and SERV-K1 Env in rhesus macaque r02120. As anticipated, we detected robust antibody responses to SIVmac239 Env (Fig. 4a). However, we also observed robust antibody responses against SERV-K1 Env in the serum of this macaque (Fig. 4b). As the sequence homology between these two envelopes is low (~19 % amino acid homology), the SERV-K1 Env-specific antibodies detected are unlikely the result of cross-reactive SIVmac239 Env-specific antibodies. Furthermore, we found similar antibody targeting of SERV-K1 Env in 3 additional SIV-infected and 2 SIV-naïve rhesus macaques (Fig. 4b). Interestingly, one of the highly targeted regions in this macaque, SERV-K1 Env_541–555_ TM, is fully conserved with HERV-K Env and a major humoral target in HIV-infected humans [10]. Focusing on six frequently targeted SERV-K1 15-mer peptides, we screened an additional 21 SIV-naïve rhesus macaques, and found consistent antibody targeting of these six SERV-K1 15-mer peptides in all animals tested (Fig. 4c). These data demonstrate that the observed SERV-K1 Env antibody responses represent genuine targeting of SERV-K1 Env and not cross-reactive SIV-specific antibody responses. Thus, SERV-K1 Env protein expression is sufficient to commonly trigger SERV-K1 Env-directed antibody responses in rhesus macaques.

### Viral infection history does not explain the presence of ERV-specific T cell responses in r02120

**Fig. 5:**
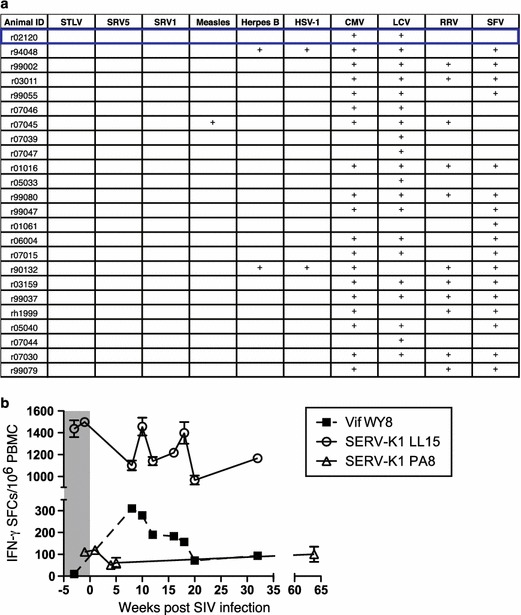
Viral infection history does not explain the presence of ERV-specific T cell responses in r02120. **a** Twenty-four rhesus macaques were screened for previous viral infections by plasma serology. Plasma was tested for antibodies against Simian T Lymphotropic virus (STLV), Simian Retrovirus Groups 1 and 5 (SRV1 and SRV5), Measles virus, Herpes B virus, Herpes Simplex virus type 1 (HSV-1), Cytomegalovirus (CMV), Lymphocryptovirus (LCV), Rhesus rhadinovirus (RRV), and Simian Foamy Virus (SFV). Rhesus macaque r02120, the only macaque in this group with detectable SERV-K1 Env-specific T cell responses, is *boxed* in *blue*. Note previous infection history does not explain r02120’s ability to mount SERV-K1 Env-specific immune responses (for example, r02120 and r07046 have identical plasma serology against these tested viruses). **b** Longitudinal ELISPOT values for IFN-γ spot forming cells (SFCs) measured in r02120 PBMC against either SERV-K1 Env LL15, SERV-K1 Env PA8, or SIV Vif WY8 are shown as mean ± SD of triplicate wells performed at the indicated times. The *grey box* indicates pre-SIV infection

Previous studies reported that infection with viruses such as HSV-1, HIV-1, and cytomegalovirus resulted in an increase in ERV expression in human cells in vitro [19, 20]. To investigate whether the viral infection history of r02120 accounted for its ability to mount SERV-K1 Env-specific T cell responses, we screened a cohort of 24 rhesus macaques, including r02120, for previous viral infections by plasma serology. The pattern of antibody detection against various retroviruses (Simian T lymphotropic virus, Simian retrovirus types one and five, Simian foamy virus), herpes viruses (Herpes B virus, Cytomegalovirus, Lymphocryptovirus, Rhesus rhadinovirus), and paramyxoviruses (Measles virus) did not reveal any correlation between previous viral infections and the presence of SERV-K1-specific immune responses (Fig. 5a). In order to specifically examine whether the observed T cell recognition of SERV-K1 Env LL15 and PA8 in r02120 was the result of cross-reactive SIV-specific T cell responses, we attempted to align these SERV-K1 Env epitopes to SIVmac239 Env and did not find any SIVmac239 Env peptides with greater than 33 and 38 % identity to SERV-K1 LL15 and PA8, respectively (Additional file 5: Figure S5). Furthermore, longitudinal analysis of both the SERV-K1 Env_526–540_ LL15-specific CD4+ T cell response and Env_109–115_ PA8-specific CD8+ T cell response in r02120 revealed that these responses were present prior to SIV infection, in contrast to a Vif_97–104_ WY8-specific CD8+ T cell response that only arose following SIV infection (Fig. 5b). Thus, the LL15- and PA8-specific T cell responses in r02120 likely represent genuine targeting of SERV-K1 Env and not cross-reactive SIV-specific T cell responses. This is in line with a previous report indicating lack of SIV-induced up-regulation of SERV activity in the cells of rhesus macaques [21]. Thus, viral infection status alone does not predict SERV-K1-specific T cell immunity.

In conclusion, these data suggest that, although ERVs have become endogenous self-antigens, ERV activity can generate specific immune responses. The mechanisms by which this ERV-specific immunity is induced and how tolerance to ERV antigens, if any, is overcome, require further investigation. However, these data contribute to the growing body of evidence that ERVs are not inert genomic fossils, but rather represent dynamic protein-coding products that impact developmental, pathological, and immunological processes.

## Methods

### Rhesus macaques

A total of 82 purpose-bred male or female rhesus macaques (RM) (*Macaca mulatta*) of Indian genetic background were used in this study including 34 SIV-infected RM, 46 SIV-naïve RM, and 2 RM with time points taken before and after SIV infection. Rhesus macaques were housed at the Wisconsin National Primate Research Center, New England Primate Research Center, or the Oregon National Primate Research Center. All protocols were approved by the respective Institutional Animal Care and Use Committees, under the standards of the US National Institutes of Health Guide for the Care and Use of Laboratory Animals.

### Identification of SERV-K1

We used HERV-K consensus sequences [23] to search the rhesus macaque genome for the simian orthologue of ERV-K. Sequence analysis was conducted using MacVector (Cary, NC, USA) and Geneious (Auckland, New Zealand) software.

### mRNA capture

Several micrograms of mRNA were purified from BLCL utilizing the Qiagen (Valencia, CA, USA) RNeasy kit and Oligotex RNA kit according to the manufacturer’s protocols. cDNA libraries were made from this mRNA with the Invitrogen (Carlsbad, CA, USA) Superscript II cDNA synthesis kit according to the manufacturer’s protocol. The cDNA was ligated into the pCMVsport vector and transformed into stbl4 electromax cells from Invitrogen (Carlsbad, CA, USA). Greater than 2 × 10^5^ colonies were plated out from the transformation and were physically scrapped off the plates to make a plasmid prep of the cDNA library. Probes of approximately 50nt in length were designed based on conserved sequences found in an alignment of multiple known ERV. The following probes were ordered from IDT (Coralville, IA, USA) as 5′ biotinylated probes that had been HPLC purified: 5′CAGCTAYGGCTGCTGTAGCAGGAGTTGCATTGCACTCTTCTGTTCAGTC3′ (envelope sequence), 5′GCTGCCAATCCTCCAGTTAACATAGATGCAGATCAACTATTAGGAATAG3′ (gag sequence), and 5′CACTATTATTAACATATACTTCAATAGGATTATCCATCCATGTAACTGCC3′ (envelope sequence). The cDNA library was enriched for the target molecules utilizing the biotinylated probes and the RecA affinity capture method of Zhumabayeva et al. [24, 25]. Specifically, the buffer described by Zhumabayeva et al. was mixed with aqueous Adenosine 5′-[γ-thiotriphosphate tetralithium salt (stored at −80 in small aliquots in a 2:1 ratio with ATP) from Sigma-Aldrich (St. Louis, MO, USA) and 50 ng of probe and 2 µg of RecA protein in a 30 µl reaction volume. This was incubated at 37° for 15 min. Then 5 µg of cDNA library was added and a further 37° incubation of 20 min was done. The RecA was then inactivated by adding 10 % SDS and Proteinase K as described by Zhumabayeva et al. The Proteinase K was incubated for 10 min at 37°. Finally, the Proteinase K was inactivated by adding 1 µl of 100 mM PMSF. Once this was thoroughly mixed, 20 µl of M-270 Dynal paramagnetic streptavidin beads from Invitrogen (Carlsbad, CA, USA) that were previously washed twice with a binding buffer consisting of 10 mM TrisHCL, 1 M NaCl, and 1 mM EDTA were added to the reaction. This was gently mixed by hand for 30 min at room temperature. The beads were washed three times with 10 mM TrisHCl, 2 M NaCl, and 1 mM EDTA wash buffer to remove unbound plasmid. A last wash was done with pure water at 37°. The beads were resuspended in 50 µl of TE buffer and extracted with an equal volume of phenol:chloroform:isoamyl alcohol. The aqueous phase was ethanol precipitated and resuspended in 10 µl of TE of which 0.5 µl was used for heat shock transformation of stbl3 cells from Invitrogen (Carlsbad, CA, USA). The resulting colonies were plasmid purified and Sanger sequenced to screen for the target sequences. The resulting clones are typically enriched for target molecule to better than 50 %, although the percentages can vary widely. The non-target molecules are generally random in their nature and probably represent non-specific sticking to the streptavidin beads. Sequence analysis was conducted using MacVector (Cary, NC, USA) and Geneious (Auckland, New Zealand) software. Accesssion numbers for captured SERV-K1 Env mRNA are KU363810 and KU363811.

### T cell assays

ELISPOT screening was carried out as previous described [26] using 15-mer peptides (with 11 amino acid overlap) spanning the entire SERV-K1 Env open reading frame or the entire SIVmac239 proteome. Intracellular cytokine staining assays were carried out as previously described [21, 26]. Positive responses shown were confirmed using peptides synthesized by three independent sources.

### Antibody detection

Heat-inactivated serum was diluted 1:1000 and used in a peptide-based ELISA assay, as previously described [22], against overlapping 15-mer peptides (with 11 amino acid overlap) spanning either the entire SERV-K1 Env open reading frame or the entire SIVmac239 Env open reading frame. ELISA was conducted in duplicate, using absorbance at 450 nm to determine binding antibodies.

### Serological tests for antibodies

Intuitive Biosciences performed serology assays. Individual serum samples were screened for presence of antibodies to a panel of viral pathogens using CSA: Simian Expanded Array and CSA: Simian Detection Kit (Intuitive Biosciences, Madison WI), following the standard manufacturer’s protocol. Briefly, samples were diluted 1:100 in the supplied Sample Dilution Buffer and incubated on the CSA: Simian Expanded Array for 1 hour at room temperature. Each sample well was washed five times with Wash Buffer, and a 1:5000 dilution of anti-Simian IgG in Sample Dilution Buffer was added to each well and incubated at room temperature for 1 h. After washing fives times with Wash Buffer, a 1:100 dilution of gold conjugate reagent in gold conjugated diluent was added to each well and incubated at room temperature for 45 min. After five repeat washes with Wash Buffer, each well was rinsed using 1× rinse buffer. SilverQuant reagent A and B were quickly mixed and added to each well, incubating for 3 min while protected from light. Each sample well was quickly rinsed with ultrapure water several times, and the arrays dried with nitrogen gas at approximately 80 psi. Each array was scanned and analyzed using the AthenaQuant System (Intuitive Biosciences). A report was generated with intensity of each spot recorded by antigen as a mean of five replicate spots, in relative intensity units. Cut off values as specified by the manufacturer were used to determine positive and negative designations for each sample. When a sample generates significant intensity of signal on the array, this indicates that the animal is seropositive to the virus represented by the antigen on the array. This does not detect presence of virus, merely presence of specific IgG to the virus, which is indicative of previous exposure or latent and/or subclinical infection.

## Additional files

10.1186/s12977-016-0238-0 **Figure S1.** SERV-K1 proviral sequence. Nucleotide sequence of SERV-K1 provirus from chromosome 12 of rhesus macaque r02120 is shown. Long terminal repeat sequences (LTRs) as well as coding sequences for Gag, Pro, Pol, and Env open reading frames are annotated. Note Pro and Pol ORFs contain frameshift mutations resulting in premature stop codons, while Gag and Env ORFs appear intact. RF = reading frame.
10.1186/s12977-016-0238-0 **Figure S2.** Alignment of captured SERV-K1 Env mRNA to SERV-K1 provirus. Protein alignment of the predicted amino acid sequence of chromosome 12 provirus and two mRNA variants captured from rhesus macaque r02120 is shown. Matches at each amino acid position are indicated with asterisks (*), while mismatches are indicated with periods (.). Mismatches in each sequence are highlighted in yellow.
10.1186/s12977-016-0238-0 **Figure S3.** Alignment of SERV-K to primate ERVs. Nucleotide alignment showing part of the *pol* locus of SERV-K from rhesus macaque r02120 aligned to various ERVs from hominids (humans, chimpanzees) and old world monkeys. Matches at each amino acid position are indicated with asterisks (*). The SERV-K1 locus described here is highlighted in grey. CERV = chimpanzee endogenous retrovirus; HERV = human endogenous retrovirus. Sequences shown here were used to construct phylogram in Fig. 1d.
10.1186/s12977-016-0238-0 **Figure S4.** ELISPOT titration of SERV-K1 Env-specific T cell responses. **a** Titration of SERV-K1 Env LL15-specific CD4+ T cell response in r02120 (1 week prior to SIV infection) using decreasing concentrations of peptide. Influenza nucleoprotein 15-mer MQ15 is included as a negative control. **b** Titration of SERV-K1 Env PA8-specific CD8+ T cell response in r02120 (20 weeks post-SIV infection) using decreasing concentrations of peptide.
10.1186/s12977-016-0238-0 **Figure S5.** Alignments of SERV-K1 Env T cell epitopes to SIVmac239 Env peptides. **a** Amino acid alignment of r02120 CD4+ T cell epitope SERV-K1 Env LL15 to SIVmac239 Env LL15. This is the only SIVmac239 Env peptide with 10 or less mismatches to SERV-K1 Env LL15. **b** Amino acid alignment of r02120 CD8+ T cell epitope SERV-K1 Env PA8 to SIVmac239 Env PC8 and AQ8. These are the only SIVmac239 Env peptides with 5 or less mismatches to SERV-K1 Env PA8. For both **a** and **b**, matches to the SERV-K1 Env peptide are shown as dashes.

## Abbreviations

ERV: endogenous retrovirus
SERV-K1: simian ERV-K 1
HERV: human endogenous retrovirus
HIV: human immunodeficiency virus
SIV: simian immunodeficiency virus
HSV-1: herpes simplex virus type 1
