# Supplementary material for: Identification and spontaneous immune targeting of an endogenous retrovirus K envelope protein in the Indian rhesus macaque model of human disease
Source: Retrovirology. 2016 Jan 15;13:6. doi: 10.1186/s12977-016-0238-0 (PMC4714462; doi:10.1186/s12977-016-0238-0)
Supplement: Supplementary file 1 — 10.1186/s12977-016-0238-0 Figure S1. SERV-K1 proviral sequence. Nucleotide sequence of SERV-K1 provirus from chromosome 12 of rhesus macaque r02120 is shown. Long terminal repeat sequences (LTRs) as well as coding sequences for Gag, Pro, Pol, and Env open reading frames are annotated. Note Pro and Pol ORFs contain frameshift mutations resulting in premature stop codons, while Gag and Env ORFs appear intact. RF = reading frame. [file 12977_2016_238_MOESM1_ESM.pdf]

Figure S1. SERV-K1 proviral sequence. Nucleotide sequence of SERV-K1 provirus from chromosome 12 of rhesus macaque r02120 is shown. Long terminal repeat sequences (LTRs) as well as coding sequences for Gag, Pro, Pol, and Env open reading frames are annotated. Note Pro and Pol ORFs contain frameshift mutations resulting in premature stop codons, while Gag and Env ORFs appear intact. RF= reading frame.

```
        10        20        30        40        50        60        70        80        90       100       110       120
TGTGGGGAAAAGAAAGAGATCAGCCTGTTACTGTGTCTATATAGAAAGAAGTAGACATAAGAGACTCCATCTTGTTCTGTATTTGAGATGCTGTTAATCTGTGACCCTACCCCCAACCTT
ACACCCCTTTTCTTTCTCTAGTCGGACAATGACACAGATATATCTTTCTTCATCTGTATTCTCTGAGGTAGAACAAGACATAAACTCTACGACAATTAGACACTGGGATGGGGGTTGGAA
________________________________________________________"5'LTR"_________________________________________________________>

       130       140       150       160       170       180       190       200       210       220       230       240
GTCCTTGCAAGAGACATGTGCTGTGGTAACTCAAGGTTTAATGGATTTTGGGCGTGCAGGGTGTGACTTTGTTCCTAGAAAAGCTAGGTATTGTCCAAGGTTTATCCCCATGTGATAGGA
CAGGAACGTTCTCTGTACACGACACCATTGAGTTCCAAATTACCTAAAACCCGCACGTCCCACACTGAAACAAGGATCTTTTCGATCCATAACAGGTTCCAAATAGGGGTACACTATCCT
________________________________________________________"5'LTR"_________________________________________________________>

       250       260       270       280       290       300       310       320       330       340       350       360
TGAAACAATGTTGCTAAAAGGTTTATCTCAAGGCACAGGATTTACCTTTAAACTTATTCATGTCACAGATCCTTGTTCTTATGTCTTACTGCTAATTTCCTCCCTAAAAATGATCCTATT
ACTTTGTTACAACGATTTTCCAAATAGAGTTCCGTGTCCTAAATGGAAATTTGAATAAGTACAGTGTCTAGGAACAAGAATACAGAATGACGATTAAAGGAGGGATTTTTACTAGGATAA
________________________________________________________"5'LTR"_________________________________________________________>

       370       380       390       400       410       420       430       440       450       460       470       480
GTCCTGCCACTCCCTTATCTTTAAGATGGTAAAGATAATTATCTATAAATACTAAGGGAACTCAGAGGCCGGTGCCGGCGTGGGTCCTCTGTAAGCTGAGCGCCGGTCCCCTGGCCCCC
CAGGACGGTGAGGGAATAGAAATTCTACCATTTCTATTAATAGATATTTATGATTCCCTTGAGTCTCCGGCCACGGCCGCACCCAGGAGACATTCGACTCGCGGCCAGGGGACCCGGGGG
________________________________________________________"5'LTR"_________________________________________________________>

       490       500       510       520       530       540       550       560       570       580       590       600
GCTTTTCTTTCTCTATACTTTGTCTCTGTGTCTTATTTCTTTTCTCAAGTCTCTCGTTCCACCTTACGAGAAACACCCACAGGTGTGGAGGGGCAGGCCACCCCTTCATCTGGTGCCCAA
CGAAAAGAAAGAGATATGAAACAGAGACACAGAATAAAGAAAAGAGTTCAGAGAGCAAGGTGGAATGCTCTTTGTGGGTGTCCACACCTCCCCGTCCGGTGGGGAAGTAGACCACGGGTT
__________________________________________________"5'LTR"___________________________________________________>

       610       620       630       640       650       660       670       680       690       700       710       720
CGTGGAAGCTTTTCTCTAAGGTGAAGGTACGCTGGAGCGTGGTCACTGAGGACAAGTCGACGAGAGACTCCCGAGTACGTCTACAGTCAGCCTTGCGGTAAGCTTGTGCGCTCGGAAGAA
GCACCTTCGAAAAGAGATTCCACTTCCATGCGACCTCGCACCAGTGACTCCTGTTCAGCTGCTCTCTGAGGGCTCATGCAGATGTCAGTCGGAACGCCATTCGAACACGCGAGCCTTCTT

       730       740       750       760       770       780       790       800       810       820       830       840
CCTAGGGTAACAATGGGGCAAACTAAGAGTAAATATGCCTCTTATCGCAGCTTTATTAAAATTCTCTTAAAAAGAGGGGGAGTTAAAGTCTCTACCAAAAATCTAATTACGCTATTTCAA
GGATCCCATTGTTACCCCGTTTGATTCTCATTTATACGGAGAATAGCGTCGAAATAATTTTAAGAGAATTTTTCTCCCCCTCAATTTCAGAGATGGTTTTTAGATTAATGCGATAAAGTT
             M  G  Q  T  K  S  K  Y  A  S  Y  R  S  F  I  K  I  L  L  K  R  G  G  V  K  V  S  T  K  N  L  I  T  L  F  Q>
            ___________________________________________________"GAG"____________________________________________________>

       850       860       870       880       890       900       910       920       930       940       950       960
ACGATAGAGCAGTTTTGTCCATGGTTTCCGGAACAGGGAACTTTAGATCTAAAAGATTGGGAAAAAATTGGCAAAGAATTAAAACAAGCAAGCAGGGAAGGTAAAATCATTCCGCTCACA
TGCTATCTCGTCAAAACAGGTACCAAAGGCCTTGTCCCTTGAAATCTAGATTTTCTAACCCTTTTTTAACCGTTTCTTAATTTTGTTCGTTCGTCCCTTCCATTTTAGTAAGGCGAGTGT
 T  I  E  Q  F  C  P  W  F  P  E  Q  G  T  L  D  L  K  D  W  E  K  I  G  K  E  L  K  Q  A  S  R  E  G  K  I  I  P  L  T>
_________________________________________________________"GAG"__________________________________________________________>

       970       980       990      1000      1010      1020      1030      1040      1050      1060      1070      1080
GTATGGAATGATTGGGTCATTATTAAAGTAGCTTTAGAACCATTTCAAACAGAAGAAGATAGCGTTCCAATTTCTGATGTCCCTAAAAGCTGTGCAGTAGATTGTGAAAAAGAGGCAGGG
CATACCTTACTAACCCAGTAATAATTTCATCGAAATCTTGGTAAAGTTTGTCTTCTTCTATCGCAAGGTTAAAGACTACAGGGATTTTCGACACGTCATCTAACACTTTTTCTCCGTCCC
 V  W  N  D  W  V  I  I  K  V  A  L  E  P  F  Q  T  E  E  D  S  V  P  I  S  D  V  P  K  S  C  A  V  D  C  E  K  E  A  G>
_________________________________________________________"GAG"__________________________________________________________>

      1090      1100      1110      1120      1130      1140      1150      1160      1170      1180      1190      1200
ATAGAATCCCGGAAAGGAAAGGAAAGTTCACACTGTGAATGTGTATCAGAGCCGGCTATGGCTCGGTCAACGCAAAATGTTGACGATAATCAATTACAGGAGGTAGTATATCCTGAAACG
TATCTTAGGGCCTTTCCTTTCCTTTCAAGTGTGACACTTACACATAGTCTCGGCCGATACCGAGCCAGTTGCGTTTTACAACTGCTATTAGTTAATGTCCTCCATCATATAGGACTTTGC
 I  E  S  R  K  G  K  E  S  S  H  C  E  C  V  S  E  P  A  M  A  R  S  T  Q  N  V  D  D  N  Q  L  Q  E  V  V  Y  P  E  T>
_________________________________________________________"GAG"__________________________________________________________>

      1210      1220      1230      1240      1250      1260      1270      1280      1290      1300      1310      1320
TTAAAATTAGAAGAAAAAAATCCAGAATTAGCAGAGCCATCAGAGTCTAAACCACGATGGCCAACTGCTCTTCCAGCAGCTCAAATGCCTGTAACTTTACAACCTCAAATGCAGGTTAAA
AATTTTAATCTTCTTTTTTTAGGTCTTAATCGTCTCGGTAGTCTCAGATTTGGTGCTACCGGTTGACGAGAAGGTCGTCGAGTTTACGGACATTGAAATGTTGGAGTTTACGTCCAATTT
 L  K  L  E  E  K  N  P  E  L  A  E  P  S  E  S  K  P  R  W  P  T  A  L  P  A  A  Q  M  P  V  T  L  Q  P  Q  M  Q  V  K>
_________________________________________________________"GAG"__________________________________________________________>

      1330      1340      1350      1360      1370      1380      1390      1400      1410      1420      1430      1440
CAAGTACAAACTGCAAAAGAAGATCAAATAGAAAAAGATAGAGTTTCTGTCATGGCAATGCCATTCCAAATGCAGTGTCCACAATATCAGCCGGTAGAAGATAAGACCCAGCCGCCAGTA
GTTCATGTTTGACGTTTTCTTCTAGTTTATCTTTTTCTATCTCAAAGACAGTACCGTTACGGTAAGGTTTACGTCACAGGTGTTATAGTCGGCCATCTTCTATTCTGGGTCGGCGGTCAT
 Q  V  Q  T  A  K  E  D  Q  I  E  K  D  R  V  S  V  M  A  M  P  F  Q  M  Q  C  P  Q  Y  Q  P  V  E  D  K  T  Q  P  P  V>
_________________________________________________________"GAG"__________________________________________________________>

      1450      1460      1470      1480      1490      1500      1510      1520      1530      1540      1550      1560
GCCTATCAATACTGGCTGCCAGCCGAACTGTGGTATCGGTTGCCCCCAGAAAATCTGTGTGGACAGCCAGGAACATTTCCAGCGCCACAGGGCAGGGTACCATATCCTCAACCGCCCACC
CGGATAGTTATGACCGACGGTCGGCTTGACACCATAGCCAACGGGGGTCTTTTAGACACACCTGTCGGTCCTTGTAAAGGTCGCGGTGTCCCGTCCCATGGTATAGGAGTTGGCGGGTGG
 A  Y  Q  Y  W  L  P  A  E  L  W  Y  R  L  P  P  E  N  L  C  G  Q  P  G  T  F  P  A  P  Q  G  R  V  P  Y  P  Q  P  P  T>
_________________________________________________________"GAG"__________________________________________________________>

      1570      1580      1590      1600      1610      1620      1630      1640      1650      1660      1670      1680
ATGAGACTTAATCCTACAGCACTGCCTAGTACACAGGGTAGTGCGTTACATAAAATTATTGATGAGGCAAGAAAACAAGGAGATATTGAGGCGTGGCAATTCCCAGTAATATTAGAAGCA
TACTCTGAATTAGGATGTCGTGACGGATCATGTGTCCCATCACGCAATGTATTTTAATAACTACTCCGTTCTTTTGTTCCTCTATAACTCCGCACCGTTAAGGGTCATTATAATCTTCGT
 M  R  L  N  P  T  A  L  P  S  T  Q  G  S  A  L  H  K  I  I  D  E  A  R  K  Q  G  D  I  E  A  W  Q  F  P  V  I  L  E  A>
_________________________________________________________"GAG"__________________________________________________________>

      1690      1700      1710      1720      1730      1740      1750      1760      1770      1780      1790      1800
AGACCACCTGGAGAAGGGGCCCAAGAGGGAGAGCTTCCCGTAGCTGAAGCCAGATATAAGTCTTTTTCTATAAAAATGCTAAAAGAAATGAAAGAGGGAGTAAAACAGTATGGACCCAAC
TCTGGTGGACCTCTTCCCCGGGTTCTCCCTCTCGAAGGGCATCGACTTCGGTCTATATTCAGAAAAAGATATTTTTACGATTTTCTTTACTTTCTCCCTCATTTTGTCATACCTGGGTTG
 R  P  P  G  E  G  A  Q  E  G  E  L  P  V  A  E  A  R  Y  K  S  F  S  I  K  M  L  K  E  M  K  E  G  V  K  Q  Y  G  P  N>
_________________________________________________________"GAG"__________________________________________________________>

      1810      1820      1830      1840      1850      1860      1870      1880      1890      1900      1910      1920
```

```
TCTCCTTACATGAGAACATTATTAGATTCCATTGCTCATGGACATAGACTCATTCCTTATGATTGGGAGATTCTGGCAAAATCCTCACTCTCACCCTCTCAGTTTTTACAATTTAAGACT
AGAGGAATGTACTCTTGTAATAATCTAAGGTAACGAGTACCTGTATCTGAGTAAGGAATACTAACCCTCTAAGACCGTTTTAGGAGTGAGAGTGGGAGAGTCAAAAATGTTAAATTCTGA
 S  P  Y  M  R  T  L  L  D  S  I  A  H  G  H  R  L  I  P  Y  D  W  E  I  L  A  K  S  S  L  S  P  S  Q  F  L  Q  F  K  T>
_____________________________________________________________"GAG"___________________________________________________________>

      1930      1940      1950      1960      1970      1980      1990      2000      2010      2020      2030      2040
TGGTGGATTGATGGGGCACAAGTACAGGTCCGAAAAAATAGGACTGCCAATCCTCCAATTGACATAGATGCAGATCAACTATTAGGAATAGGTCAAAATTGGAGCACTGTTGACCAACAA
ACCACCTAACTACCCCGTGTTCATGTCCAGGCTTTTTTATCCTGACGGTTAGGAGGTTAACTGTATCTACGTCTAGTTGATAATCCTTATCCAGTTTTAACCTCGTGACAACTGGTTGTT
 W  W  I  D  G  A  Q  V  Q  V  R  K  N  R  T  A  N  P  P  I  D  I  D  A  D  Q  L  L  G  I  G  Q  N  W  S  T  V  D  Q  Q>
_____________________________________________________________"GAG"___________________________________________________________>

      2050      2060      2070      2080      2090      2100      2110      2120      2130      2140      2150      2160
GTAATAATGCCAAATGAGGCCATTGAGCAAATCAGGGCTATCTGCCTTAGGGCCTGGGAGAAAATCCAAGACCCAGGAACCGCCTGCCCCTCCTTTAATACAATAAGACAAGGCTCTAAA
CATTATTACGGTTTACTCCGGTAACTCGTTTAGTCCCGATAGACGGAATCCCGGACCCTCTTTTAGGTTCTGGGTCCTTGGCGGACGGGGAGGAAATTATGTTATTCTGTTCCGAGATTT
 V  I  M  P  N  E  A  I  E  Q  I  R  A  I  C  L  R  A  W  E  K  I  Q  D  P  G  T  A  C  P  S  F  N  T  I  R  Q  G  S  K>
_____________________________________________________________"GAG"___________________________________________________________>

      2170      2180      2190      2200      2210      2220      2230      2240      2250      2260      2270      2280
GAGCCCTACCCTGATTTTGTAGCAAGACTTCAAGATGCTGCTCAAAAGTCAATTACCGATGAGAATGCCCGTAAGGTCATAGTGGAGTTGATGGCATATGAAAACGCCAATCCTGATTGT
CTCGGGATGGGACTAAAACATCGTTCTGAAGTTCTACGACGAGTTTTCAGTTAATGGCTACTCTTACGGGCATTCCAGTATCACCTCAACTACCGTATACTTTTGCGGTTAGGACTAACA
 E  P  Y  P  D  F  V  A  R  L  Q  D  A  A  Q  K  S  I  T  D  E  N  A  R  K  V  I  V  E  L  M  A  Y  E  N  A  N  P  D  C>
_____________________________________________________________"GAG"___________________________________________________________>

      2290      2300      2310      2320      2330      2340      2350      2360      2370      2380      2390      2400
CAATCAGCCATTAAGCCATTAAAAGGAAAGGTTCCCGCAGGATCAGATGTAATCTCAGAGTACGTTAAGGCCTGCGATGGAATTGGAGGAGCTATGCATAAAGCTATGCTTATGGCTCAA
GTTAGTCGGTAATTCGGTAATTTTCCTTTCCAAGGGCGTCCTAGTCTACATTAGAGTCTCATGCAATTCCGGACGCTACCTTAACCTCCTCGATACGTATTTCGATACGAATACCGAGTT
 Q  S  A  I  K  P  L  K  G  K  V  P  A  G  S  D  V  I  S  E  Y  V  K  A  C  D  G  I  G  G  A  M  H  K  A  M  L  M  A  Q>
_____________________________________________________________"GAG"___________________________________________________________>

      2410      2420      2430      2440      2450      2460      2470      2480      2490      2500      2510      2520
GCAATCACAGGAGTTGCTTTAGGAGGACAGGTTAGAACATTTGGGGGAAGATGTTATAATTGTGGTCAAATTGGTCATCTAAAAAAGAATTGCCTAGTCTCAAATAAACAAAATGTAACT
CGTTAGTGTCCTCAACGAAATCCTCCTGTCCAATCTTGTAAACCCCCTTCTACAATATTAACACCAGTTTAACCAGTAGATTTTTTCTTAACGGATCAGAGTTTATTTGTTTTACATTGA
 A  I  T  G  V  A  L  G  G  Q  V  R  T  F  G  G  R  C  Y  N  C  G  Q  I  G  H  L  K  K  N  C  L  V  S  N  K  Q  N  V  T>
_____________________________________________________________"GAG"___________________________________________________________>

      2530      2540      2550      2560      2570      2580      2590      2600      2610      2620      2630      2640
ACTCAAGCTACTACAAGAACAGATAAAGAGCCACCTGGCCTATGTCCAAGATGTAAAAAGGGAAAACATTGGGGTGATCAATGTCGTTCTAAATTTGATAAAAATGGGCAACTACTGTCG
TGAGTTCGATGATGTTCTTGTCTATTTCTCGGTGGACCGGATACAGGTTCTACATTTTTCCCTTTTGTAACCCCACTAGTTACAGCAAGATTTAAACTATTTTTACCCGTTGATGACAGC
 T  Q  A  T  T  R  T  D  K  E  P  P  G  L  C  P  R  C  K  K  G  K  H  W  G  D  Q  C  R  S  K  F  D  K  N  G  Q  L  L  S>
_____________________________________________________________"GAG"___________________________________________________________>
                                                                                                             K  W  A  T  T  V>
                                                                                                            ___"PROTEASE RF____>

      2650      2660      2670      2680      2690      2700      2710      2720      2730      2740      2750      2760
GGAAACGAGAGGAGGGGCCAGCCTCAGGCCCCGCAACAAACTGGGGCATTCCAAATTCAGCCCTTTGTTCCTCAGGGTTTTCAGGAACAACAACCCCCACTGCCACAAGTGTCTCAGGGA
CCTTTGCTCTCCTCCCCGGTCGGAGTCCGGGGCGTTGTTTGACCCCGTAAGGTTTAAGTCGGGAAACAAGGAGTCCCAAAAGTCCTTGTTGTTGGGGGTGACGGTGTTCACAGAGTCCCT
 G  N  E  R  R  G  Q  P  Q  A  P  Q  Q  T  G  A  F  Q  I  Q  P  F  V  P  Q  G  F  Q  E  Q  Q  P  P  L  P  Q  V  S  Q  G>
_____________________________________________________________"GAG"___________________________________________________________>
G  K  R  E  E  G  P  A  S  G  P  A  T  N  W  G  I  P  N  S  A  L  C  S  S  G  F  S  G  T  T  T  P  T  A  T  S  V  S  G>
_______________________________________________________"PROTEASE RF1"_________________________________________________________>

      2770      2780      2790      2800      2810      2820      2830      2840      2850      2860      2870      2880
ATAAGCCAGTTATCACAATACAGCAATTATTCCCCGCCACAAACGGCAGTGCAGCAGTAGATTTATGCACCATACAAGCAGTCTCTCTGCTTCCAGGGGAGCCTCCACAAAAAATCCCCA
TATTCGGTCAATAGTGTTATGTCGTTAATAAGGGGCGGTGTTTGCCGTCACGTCGTCATCTAAATACGTGGTATGTTCGTCAGAGAGACGAAGGTCCCCTCGGAGGTGTTTTTTAGGGGT
 I  S  Q  L  S  Q  Y  S  N  Y  S  P  P  Q  T  A  V  Q  Q  *>
____________________________"GAG"____________________________>
N  K  P  V  I  T  I  Q  Q  L  F  P  A  T  N  G  S  A  A  V  D  L  C  T  I  Q  A  V  S  L  L  P  G  E  P  P  Q  K  I  P>
_______________________________________________________"PROTEASE RF1"_________________________________________________________>

      2890      2900      2910      2920      2930      2940      2950      2960      2970      2980      2990      3000
CAGGGGTATATGGCCCATTGCCTGAGGGGACTGTAGGACTAATCTTAGGAAGATCAAGTTTAAATCTAAAAGGAGTTCAAATTCATACTGGTGTGGTTGATTCAGACTTTAAAGGCGAAA
GTCCCCATATACCGGGTAACGGACTCCCCTGACATCCTGATTAGAATCCTTCTAGTTCAAATTTAGATTTTCCTCAAGTTTAAGTATGACCACACCAACTAAGTCTGAAATTTCCGCTTT
T  G  V  Y  G  P  L  P  E  G  T  V  G  L  I  L  G  R  S  S  L  N  L  K  G  V  Q  I  H  T  G  V  V  D  S  D  F  K  G  E>
_______________________________________________________"PROTEASE RF1"_________________________________________________________>

      3010      3020      3030      3040      3050      3060      3070      3080      3090      3100      3110      3120
TTCAATTGGTTATTAGTTCCTCAATTCCTTGGAGTGCCAGTCCAGGAGACAGGATTGCTCAATTATTACTCCTACCTTATATTAAAGTTGGAAACAGTGAGATAAAAAGAACAGGAGGGT
AAGTTAACCAATAATCAAGGAGTTAAGGAACCTCACGGTCAGGTCCTCTGTCCTAACGAGTTAATAATGAGGATGGAATATAATTTCAACCTTTGTCACTCTATTTTTCTTGTCCTCCCA
I  Q  L  V  I  S  S  S  I  P  W  S  A  S  P  G  D  R  I  A  Q  L  L  L  L  P  Y  I  K  V  G  N  S  E  I  K  R  T  G  G>
_______________________________________________________"PROTEASE RF1"_________________________________________________________>

      3130      3140      3150      3160      3170      3180      3190      3200      3210      3220      3230      3240
TCGGAAGCACTGATCCGGCAGGAAAGGCTGCATATTGGGCAAATCTGGTCTCTGAGAGCAGACCTGTGTGTAAGGCCATTATTCAAGGAAAACAGTTTTGAAGGGTTGGTAGACACTGGA
AGCCTTCGTGACTAGGCCGTCCTTTCCGACGTATAACCCGTTTAGACCAGAGACTCTCGTCTGGACACACATTCCGGTAATAAGTTCCTTTTGTCAAAACTTCCCAACCATCTGTGACCT
F  G  S  T  D  P  A  G  K  A  A  Y  W  A  N  L  V  S  E  S  R  P  V  C  K  A  I  I  Q  G  K  Q  F  *>
______________________________________________"PROTEASE RF1"_______________________________________________>
                                                                                                           E  G  L  V  D  T  G>
                                                                                                          ___"PROTEASE RF2"____>

      3250      3260      3270      3280      3290      3300      3310      3320      3330      3340      3350      3360
GCAGATGTCTCTGTCATTGCTTTAAATCAGTGGCCAAAAAATTGGCCTAAACAAAAGGCTGTTACAAGACTTGTCGGCGTAGGCACAGCTTCAGAAGTGTACGAAAGTACAATGATTTTA
CGTCTACAGAGACAGTAACGAAATTTAGTCACCGGTTTTTTAACCGGATTTGTTTTCCGACAATGTTCTGAACAGCCGCATCCGTGTCGAAGTCTTCACATGCTTTCATGTTACTAAAAT
 A  D  V  S  V  I  A  L  N  Q  W  P  K  N  W  P  K  Q  K  A  V  T  R  L  V  G  V  G  T  A  S  E  V  Y  E  S  T  M  I  L>
_______________________________________________________"PROTEASE RF2"_________________________________________________________>

      3370      3380      3390      3400      3410      3420      3430      3440      3450      3460      3470      3480
CATTGTTTAGGACCAGATAATCAAGAAAGTACTGTTCAGCCAATGATTACTTCAATTCCTGTTAATCTATGGGGTCGAGATTTATTACAGCAATGGGGTGCGGAAATCATTATGCCCGCT
GTAACAAATCCTGGTCTATTAGTTCTTTCATGACAAGTCGGTTACTAATGAAGTTAAGGACAATTAGATACCCCAGCTCTAAATAATGTCGTTACCCCACGCCTTTAGTAATACGGGCGA
 H  C  L  G  P  D  N  Q  E  S  T  V  Q  P  M  I  T  S  I  P  V  N  L  W  G  R  D  L  L  Q  Q  W  G  A  E  I  I  M  P  A>
_______________________________________________________"PROTEASE RF2"_________________________________________________________>
```

```
      3490       3500       3510       3520       3530       3540       3550       3560       3570       3580       3590       3600
CCATTATACAGCCCCACAAGTCAAAAAATCATAACTAAGATGGGATATATACCAGGAAAGGGGTTAGGGAAAAATGAAAATGGCATTAAAGTCCCAATTGAGACTAAGAAAAATCAAGAA
GGTAATATGTCGGGGTGTTCAGTTTTTTAGTATTGATTCTACCCTATATATGGTCCTTTCCCCAATCCCTTTTTACTTTTACCGTAATTTCAGGGTTAACTCTGATTCTTTTTAGTTCTT
 P  L  Y  S  P  T  S  Q  K  I  I  T  K  M  G  Y  I  P  G  K  G  L  G  K  N  E  N  G  I  K  V  P  I  E  T  K  K  N  Q  E>
____________________________________________________"PROTEASE RF2"____________________________________________________>
                                                                                                              E  K  S  R>
                                                                                                            _____________>

      3610       3620       3630       3640       3650       3660       3670       3680       3690       3700       3710       3720
AGAAAAGGAATAGGGTATCCTTTTTAGGGGCGGCCACTGTAGCGCCTCCTAAACCCATTCCATTAACTTGGAAAACAGAAAAACCGGTATGGGTAAATCAGTGGCCGCTACCGAAGCAAA
TCTTTTCCTTATCCCATAGGAAAAATCCCCGCCGGTGACATCGCGGAGGATTTGGGTAAGGTAATTGAACCTTTTGTCTTTTTGGCCATACCCATTTAGTCACCGGCGATGGCTTCGTTT
 R  K  G  I  G  Y  P  F  *>
______"PROTEASE RF2"_______>
K  K  R  N  R  V  S  F  L  G  A  A  T  V  A  P  P  K  P  I  P  L  T  W  K  T  E  K  P  V  W  V  N  Q  W  P  L  P  K  Q>
_______________________________________________________"POL RF1"_______________________________________________________>

      3730       3740       3750       3760       3770       3780       3790       3800       3810       3820       3830       3840
AACTGGAGGCTTTACATTCATTAGCAAAGGAACAATTTAAAAAAGGACACATTGAGCCTTCATTCTCACCCTGGAATTCTCCTGTATTTGTAATACAGAAAAAATCAGGCAAATGGCGTA
TTGACCTCCGAAATGTAAGTAATCGTTTCCTTGTTAAATTTTTTCCTGTGTAACTCGGAAGTAAGAGTGGGACCTTAAGAGGACATAAACATTATGTCTTTTTTAGTCCGTTTACCGCAT
K  L  E  A  L  H  S  L  A  K  E  Q  F  K  K  G  H  I  E  P  S  F  S  P  W  N  S  P  V  F  V  I  Q  K  K  S  G  K  W  R>
_______________________________________________________"POL RF1"_______________________________________________________>

      3850       3860       3870       3880       3890       3900       3910       3920       3930       3940       3950       3960
TGTTAATGGACTTAAGAGCCGTAAATGCTGTAATTCAACCCATGGGGCCTCTTCAACCCGGATTGCCCTCTCCGGCCATGATCCCAAAAGACTGGCCTTTAATTATAATTGATCTTAAGG
ACAATTACCTGAATTCTCGGCATTTACGACATTAAGTTGGGTACCCCGGAGAAGTTGGGCCTAACGGGAGAGGCCGGTACTAGGGTTTTCTGACCGGAAATTAATATTAACTAGAATTCC
M  L  M  D  L  R  A  V  N  A  V  I  Q  P  M  G  P  L  Q  P  G  L  P  S  P  A  M  I  P  K  D  W  P  L  I  I  I  D  L  K>
_______________________________________________________"POL RF1"_______________________________________________________>

      3970       3980       3990       4000       4010       4020       4030       4040       4050       4060       4070       4080
ATTGCTTTTTTACCATTCCTCTGGCAGAGCAAGATTGTGAAAAATTTGCCTTTACTATACCAGCCATAAATAATAAAGAACCAGCCACCAGGTTCCAGTGGAAAGTACTACCTCAGGGAA
TAACGAAAAAATGGTAAGGAGACCGTCTCGTTCTAACACTTTTTAAACGGAAATGATATGGTCGGTATTTATTATTTCTTGGTCGGTGGTCCAAGGTCACCTTTCATGATGGAGTCCCTT
D  C  F  F  T  I  P  L  A  E  Q  D  C  E  K  F  A  F  T  I  P  A  I  N  N  K  E  P  A  T  R  F  Q  W  K  V  L  P  Q  G>
_______________________________________________________"POL RF1"_______________________________________________________>

      4090       4100       4110       4120       4130       4140       4150       4160       4170       4180       4190       4200
TGCTTAATAGTCCAACTATTTGTCAAACTTTCGTAGGTCAAGTCCTTCAACCAGTTAGAGACAAATTTTCAGATTGTTATATCATTCACTATATTGATGATATTTTATGTGCTGCAGAAA
ACGAATTATCAGGTTGATAAACAGTTTGAAAGCATCCAGTTCAGGAAGTTGGTCAATCTCTGTTTAAAAGTCTAACAATATAGTAAGTGATATAACTACTATAAAATACACGACGTCTTT
M  L  N  S  P  T  I  C  Q  T  F  V  G  Q  V  L  Q  P  V  R  D  K  F  S  D  C  Y  I  I  H  Y  I  D  D  I  L  C  A  A  E>
_______________________________________________________"POL RF1"_______________________________________________________>

      4210       4220       4230       4240       4250       4260       4270       4280       4290       4300       4310       4320
CAAGAGAAAAATTAATTGACTGTTACACATTTCTGCAAGCAGAGGTTGCCAACGCAGGACTAACAATAGCATCGGATAAGATCCAAACCTCTGCTCCTTTTCATTATTTAGGGATGCAGA
GTTCTCTTTTTAATTAACTGACAATGTGTAAAGACGTTCGTCTCCAACGGTTGCGTCCTGATTGTTATCGTAGCCTATTCTAGGTTTGGAGACGAGGAAAAGTAATAAATCCCTACGTCT
T  R  E  K  L  I  D  C  Y  T  F  L  Q  A  E  V  A  N  A  G  L  T  I  A  S  D  K  I  Q  T  S  A  P  F  H  Y  L  G  M  Q>
_______________________________________________________"POL RF1"_______________________________________________________>

      4330       4340       4350       4360       4370       4380       4390       4400       4410       4420       4430       4440
TAGAAAAGTAGAAAAAATTAAGCCACAAAAAATAGAAATAAGAAAAGATACTTTAAAAACTTTAAATGACTTTCAAAAATTGTTAGGCGATATTAATTGGATTCGGCCAACTCTAGGCATT
ATCTTTTCATCTTTTTTAATTCGGTGTTTTTTATCTTTATTCTTTTCTATGAAATTTTTGAAATTTACTGAAAGTTTTTAACAATCCGCTATAATTAACCTAAGCCGGTTGAGATCCGTAA
I  E  K  *>
___________>
          R  K  I  K  P  Q  K  I  E  I  R  K  D  T  L  K  T  L  N  D  F  Q  K  L  L  G  D  I  N  W  I  R  P  T  L  G  I>
         __________________________________________________"POL RF2"_____________________________________________________>

      4450       4460       4470       4480       4490       4500       4510       4520       4530       4540       4550       4560
CCTACTTATGCCATGTCAAATTTGTTCTCTATCCTAAGAGGAGATCCAGACTTAAATAGTAAAAGAATATTAACCCCAGAGGCAACAAAAGAAATTAAATTAGTGGAAGAAAAAATTCAG
GGATGAATACGGTACAGTTTAAACAAGAGATAGGATTCTCCTCTAGGTCTGAATTTATCATTTTCTTATAATTGGGGTCTCCGTTGTTTTCTTTAATTTAATCACCTTCTTTTTTAAGTC
 P  T  Y  A  M  S  N  L  F  S  I  L  R  G  D  P  D  L  N  S  K  R  I  L  T  P  E  A  T  K  E  I  K  L  V  E  E  K  I  Q>
_______________________________________________________"POL RF2"_______________________________________________________>

      4570       4580       4590       4600       4610       4620       4630       4640       4650       4660       4670       4680
TCAGCGCAAATAAATAGGATAGATCCTTTAGCCCCACTCCAACTTTTGATTTTTGCTACTACACATTCTCCAACAGGCATCATTATTCAAAACACTGATCTTGTGGAGTGGTCATTTCTT
AGTCGCGTTTATTTATCCTATCTAGGAAATCGGGGTGAGGTTGAAAACTAAAAACGATGATGTGTAAGAGGTTGTCCGTAGTAATAAGTTTTGTGACTAGAACACCTCACCAGTAAAGAA
 S  A  Q  I  N  R  I  D  P  L  A  P  L  Q  L  L  I  F  A  T  T  H  S  P  T  G  I  I  I  Q  N  T  D  L  V  E  W  S  F  L>
_______________________________________________________"POL RF2"_______________________________________________________>

      4690       4700       4710       4720       4730       4740       4750       4760       4770       4780       4790       4800
TCTCACAGTACAATTAAGACTTTTACATTGTACTTGGATCGAATAGCTACATTAATTGGTCAGGCAAGATTACGGATAATAAAATTGTGTGGTAATGACCCAGACAAAATAGTTGTTCCT
AGAGTGTCATGTTAATTCTGAAAATGTAACATGAACCTAGCTTATCGATGTAATTAACCAGTCCGTTCTAATGCCTATTATTTTAACACACCATTACTGGGTCTGTTTTATCAACAAGGA
 S  H  S  T  I  K  T  F  T  L  Y  L  D  R  I  A  T  L  I  G  Q  A  R  L  R  I  I  K  L  C  G  N  D  P  D  K  I  V  V  P>
_______________________________________________________"POL RF2"_______________________________________________________>

      4810       4820       4830       4840       4850       4860       4870       4880       4890       4900       4910       4920
TTAACCAAGGAACAAGTTAGGCAAGCCTTTATCAATTCTGGTGCATGGCAGATTGGCCTTGCTAATTTTGTGGGAGTTATTGATAATCATTACCCAAAAACAAAAATCTTCCAGTTTTTA
AATTGGTTCCTTGTTCAATCCGTTCGGAAATAGTTAAGACCACGTACCGTCTAACCGGAACGATTAAAACACCCTCAATAACTATTAGTAATGGGTTTTTGTTTTTAGAAGGTCAAAAAT
 L  T  K  E  Q  V  R  Q  A  F  I  N  S  G  A  W  Q  I  G  L  A  N  F  V  G  V  I  D  N  H  Y  P  K  T  K  I  F  Q  F  L>
_______________________________________________________"POL RF2"_______________________________________________________>

      4930       4940       4950       4960       4970       4980       4990       5000       5010       5020       5030       5040
AAATTGACTACTTGGATTTTACCTAAAATCACCAGACATGAGCCTTTAGAAAATGCTCTGACAGTATTTACTGATGGTTCCAGCAATGGAAAAGCGGCTTACACAGGACCAAAAGAGCGA
TTTAACTGATGAACCTAAAATGGATTTTAGTGGTCTGTACTCGGAAATCTTTTACGAGACTGTCATAAATGACTACCAAGGTCGTTACCTTTTCGCCGAATGTGTCCTGGTTTTCTCGCT
 K  L  T  T  W  I  L  P  K  I  T  R  H  E  P  L  E  N  A  L  T  V  F  T  D  G  S  S  N  G  K  A  A  Y  T  G  P  K  E  R>
_______________________________________________________"POL RF2"_______________________________________________________>

      5050       5060       5070       5080       5090       5100       5110       5120       5130       5140       5150       5160
GTAATCAAAACTCAGTATCAATCAGCTCAAAGAGCAGAGTTGGTTGCAGTCATTACAGTGTTACAAGATTTTAATCAACCTGTTAATATTATATCAGATTCTGCATATGTAGTACAGGCT
CATTAGTTTTGAGTCATAGTTAGTCGAGTTTCTCGTCTCAACCAACGTCAGTAATGTCACAATGTTCTAAAATTAGTTGGACAATTATAATATAGTCTAAGACGTATACATCATGTCCGA
 V  I  K  T  Q  Y  Q  S  A  Q  R  A  E  L  V  A  V  I  T  V  L  Q  D  F  N  Q  P  V  N  I  I  S  D  S  A  Y  V  V  Q  A>
_______________________________________________________"POL RF2"_______________________________________________________>

      5170       5180       5190       5200       5210       5220       5230       5240       5250       5260       5270       5280
```

```
ACAAGGGATGTTGAGACAGCTCTAATTAAATATAGCATGGATGATCAGTTAAACCAGTTGTTTAATTTATTACAACAAACTGTAAGAAAAAAGAATTTCTCATTTTATATTACTCATATT
TGTTCCCTACAACTCTGTCGAGATTAATTTATATCGTACCTACTAGTCAATTTGGTCAACAAATTAAATAATGTTGTTTGACATTCTTTTTTCTTAAAGAGTAAAATATAATGAGTATAA
 T  R  D  V  E  T  A  L  I  K  Y  S  M  D  D  Q  L  N  Q  L  F  N  L  L  Q  Q  T  V  R  K  K  N  F  S  F  Y  I  T  H  I>
_______________________________________________________"POL RF2"________________________________________________________>

      5290      5300      5310      5320      5330      5340      5350      5360      5370      5380      5390      5400
CGAGCACACACTAATTTGCCAGGACCTTTAACTATAGCAAATGAACAAGCTGACTTACTGGTATCCTCTGCATTCATAAAAGCACAAGAACTTCATGCTTTGACTCATGTAAATGCAGCA
GCTCGTGTGTGATTAAACGGTCCTGGAAATTGATATCGTTTACTTGTTCGACTGAATGACCATAGGAGACGTAAGTATTTTCGTGTTCTTGAAGTACGAAACTGAGTACATTTACGTCGT
 R  A  H  T  N  L  P  G  P  L  T  I  A  N  E  Q  A  D  L  L  V  S  S  A  F  I  K  A  Q  E  L  H  A  L  T  H  V  N  A  A>
_______________________________________________________"POL RF2"________________________________________________________>

      5410      5420      5430      5440      5450      5460      5470      5480      5490      5500      5510      5520
GGGTTAAAAAACAAATTTGATGTCACATGGAAACAGGCAAAAGATATTGTACGACATTGCACCCAGTGTCAAGTACTACACCTGCCCACTCAAGAGGCAGGAGTGAATCCCAGAGGTCTG
CCCAATTTTTTGTTTAAACTACAGTGTACCTTTGTCCGTTTTCTATAACATGCTGTAACGTGGGTCACAGTTCATGATGTGGACGGGTGAGTTCTCCGTCCTCACTTAGGGTCTCCAGAC
 G  L  K  N  K  F  D  V  T  W  K  Q  A  K  D  I  V  R  H  C  T  Q  C  Q  V  L  H  L  P  T  Q  E  A  G  V  N  P  R  G  L>
_______________________________________________________"POL RF2"________________________________________________________>

      5530      5540      5550      5560      5570      5580      5590      5600      5610      5620      5630      5640
TGTCCTAATGCATTATGGCAAATGGATGTTACCCATGTACCTTCATTTGGAAAATTATCATATGTTCATGTAACAGTTGATACTTATTCACATTTCATATGGGCAACATGCCAGACAGGA
ACAGGATTACGTAATACCGTTTACCTACAATGGGTACATGGAAGTAAACCTTTTAATAGTATACAAGTACATTGTCAACTATGAATAAGTGTAAAGTATACCCGTTGTACGGTCTGTCCT
 C  P  N  A  L  W  Q  M  D  V  T  H  V  P  S  F  G  K  L  S  Y  V  H  V  T  V  D  T  Y  S  H  F  I  W  A  T  C  Q  T  G>
_______________________________________________________"POL RF2"________________________________________________________>

      5650      5660      5670      5680      5690      5700      5710      5720      5730      5740      5750      5760
GAAAGTACTTCCCATGTTAAAAAACATTTATTATCTTGTTTTGCTGTAATGGGAGTTCCAGAAAAAATTAAAACTGACAATGGACCAGGTTATTGTAGTAAAGCTTTCCAAAAATTCTTA
CTTTCATGAAGGGTACAATTTTTTGTAAATAATAGAACAAAACGACATTACCCTCAAGGTCTTTTTTAATTTTGACTGTTACCTGGTCCAATAACATCATTTCGAAAGGTTTTTAAGAAT
 E  S  T  S  H  V  K  K  H  L  L  S  C  F  A  V  M  G  V  P  E  K  I  K  T  D  N  G  P  G  Y  C  S  K  A  F  Q  K  F  L>
_______________________________________________________"POL RF2"________________________________________________________>

      5770      5780      5790      5800      5810      5820      5830      5840      5850      5860      5870      5880
AATCAGTGGAATATTACACATACAACAGGAATTCCTTATAATTCCCAAGGACAGGCCATAGTTGAAAGAACTAATAGAACACTCAAAACTCAATTAATTAAACAAAAAAAAGGGGGAGAC
TTAGTCACCTTATAATGTGTATGTTGTCCTTAAGGAATATTAAGGGTTCCTGTCCGGTATCAACTTTCTTGATTATCTTGTGAGTTTTGAGTTAATTAATTTGTTTTTTTTCCCCCTCTG
 N  Q  W  N  I  T  H  T  T  G  I  P  Y  N  S  Q  G  Q  A  I  V  E  R  T  N  R  T  L  K  T  Q  L  I  K  Q  K  K  G  G  D>
_______________________________________________________"POL RF2"________________________________________________________>

      5890      5900      5910      5920      5930      5940      5950      5960      5970      5980      5990      6000
AGTAAGGAGTATACCACTCCTCAGATGCAGCTTAATCTAGCACTCTATACTTTAAATTTTTTAAACATTTATAGAAATCAGACTACTACTTCTGCAGAACAACATCTTACTGGTAAAAAG
TCATTCCTCATATGGTGAGGAGTCTACGTCGAATTAGATCGTGAGATATGAAATTTAAAAAATTTGTAAATATCTTTAGTCTGATGATGAAGACGTCTTGTTGTAGAATGACCATTTTTC
 S  K  E  Y  T  T  P  Q  M  Q  L  N  L  A  L  Y  T  L  N  F  L  N  I  Y  R  N  Q  T  T  T  S  A  E  Q  H  L  T  G  K  K>
_______________________________________________________"POL RF2"________________________________________________________>

      6010      6020      6030      6040      6050      6060      6070      6080      6090      6100      6110      6120
AACAGTCCACATGAAGGAAAACTGATTTGGTGGAAAGACAACAAAAATAAGACATGGGAAATAGGGAAGGTGATAACCTGGGGAAGAGGTTTTGCTTGTGTTTCACCAGGAGAAAATCAG
TTGTCAGGTGTACTTCCTTTTGACTAAACCACCTTTCTGTTGTTTTTATTCTGTACCCTTTATCCCTTCCACTATTGGACCCCTTCTCCAAAACGAACACAAAGTGGTCCTCTTTTAGTC
 N  S  P  H  E  G  K  L  I  W  W  K  D  N  K  N  K  T  W  E  I  G  K  V  I  T  W  G  R  G  F  A  C  V  S  P  G  E  N  Q>
_______________________________________________________"POL RF2"________________________________________________________>

      6130      6140      6150      6160      6170      6180      6190      6200      6210      6220      6230      6240
CTTCCTGTTTGGATACCCACTAAACATTTGAAGTTCTACAATGAACCCATCGGAGATGCAAAGAAAAGCGCCTCCACGGAGACAGAAACACCGCAATCGAGCACCATTGACTCGCATGAT
GAAGGACAAACCTATGGGTGATTTGTAAACTTCAAGATGTTACTTGGGTAGCCTCTACGTTTCTTTTCGCGGAGGTGCCTCTGTCTTTGTGGCGTTAGCTCGTGGTAACTGAGCGTACTA
 L  P  V  W  I  P  T  K  H  L  K  F  Y  N  E  P  I  G  D  A  K  K  S  A  S  T  E  T  E  T  P  Q  S  S  T  I  D  S  H  D>
_______________________________________________________"POL RF2"________________________________________________________>
                                         M  N  P  S  E  M  Q  R  K  A  P  P  R  R  Q  K  H  R  N  R  A  P  L  T  R  M  M>
                                        _____________________________________"ENV"______________________________________>

      6250      6260      6270      6280      6290      6300      6310      6320      6330      6340      6350      6360
GAACCAAGTGATGATATCAGAAGAACAGATGAAGTCACCACGCACCAAGAAGGCGGAGCTGCCGACCTGGGCACAGTTAAAGAAGCTGACACCGTTAGCTGGAAAAAGCCTAGCTAGCAC
CTTGGTTCACTACTATAGTCTTCTTGTCTACTTCAGTGGTGCGTGGTTCTTCCGCCTCGACGGCTGGACCCGTGTCAATTTCTTCGACTGTGGCAATCGACCTTTTTCGGATCGATCGTG
 E  P  S  D  D  I  R  R  T  D  E  V  T  T  H  Q  E  G  G  A  A  D  L  G  T  V  K  E  A  D  T  V  S  W  K  K  P  S  *  H>
_______________________________________________________"POL RF2"________________________________________________________>
  N  Q  V  M  I  S  E  E  Q  M  K  S  P  R  T  K  K  A  E  L  P  T  W  A  Q  L  K  K  L  T  P  L  A  G  K  S  L  A  S  T>
_________________________________________________________"ENV"__________________________________________________________>

      6370      6380      6390      6400      6410      6420      6430      6440      6450      6460      6470      6480
AAAGGTGACACAAACCCCAGAAAAAATGCTGCTTACAGCTTTAATGATTGTATCAACGGTGGTAAGTCTCCCCATGCCTGCAGGAGCAGCTGCAGCTAATTATACCTACTGGGCCTATGT
TTTCCACTGTGTTTGGGGTCTTTTTTACGACGAATGTCGAAATTACTAACATAGTTGCCACCATTCAGAGGGGTACGGACGTCCTCGTCGACGTCGATTAATATGGATGACCCGGATACA
 K  G  D  T  N  P  R  K  N  A  A  Y  S  F  N  D  C  I  N  G  G  K  S  P  H  A  C  R  S  S  C  S  *>
____________________________________________"POL RF2"______________________________________________>
  K  V  T  Q  T  P  E  K  M  L  L  T  A  L  M  I  V  S  T  V  V  S  L  P  M  P  A  G  A  A  A  A  N  Y  T  Y  W  A  Y  V>
_________________________________________________________"ENV"__________________________________________________________>

      6490      6500      6510      6520      6530      6540      6550      6560      6570      6580      6590      6600
GCCTTTCCCGCCCTTAATTCGGGCAGTTACATGGATGGATAATCCTATTGAAGTATATGTTAATAATAGTGTGTGGGTACCTGGTCCCACAGATGATCGTTGCCCTGCCAAACCGGAGGA
CGGAAAGGGCGGGAATTAAGCCCGTCAATGTACCTACCTATTAGGATAACTTCATATACAATTATTATCACACACCCATGGACCAGGGTGTCTACTAGCAACGGGACGGTTTGGCCTCCT
  P  F  P  P  L  I  R  A  V  T  W  M  D  N  P  I  E  V  Y  V  N  N  S  V  W  V  P  G  P  T  D  D  R  C  P  A  K  P  E  E>
_________________________________________________________"ENV"__________________________________________________________>

      6610      6620      6630      6640      6650      6660      6670      6680      6690      6700      6710      6720
AGAAGGAATGATGATAAATATTTCCATTGGGTATCGTTATCCTCCTATTTGCCTAGGGAGAGCACCAGGATGTTTAATGCCTGCTATTCAAAATTGGTTGGTAGAAGTACCTACTGTCAG
TCTTCCTTACTACTATTTATAAAGGTAACCCATAGCAATAGGAGGATAAACGGATCCCTCTCGTGGTCCTACAAATTACGGACGATAAGTTTTAACCAACCATCTTCATGGATGACAGTC
  E  G  M  M  I  N  I  S  I  G  Y  R  Y  P  P  I  C  L  G  R  A  P  G  C  L  M  P  A  I  Q  N  W  L  V  E  V  P  T  V  S>
_________________________________________________________"ENV"__________________________________________________________>

      6730      6740      6750      6760      6770      6780      6790      6800      6810      6820      6830      6840
TCCCACCAGTAGATTTACTTATCACATGGTAAGCGGAATGTCACTCAAACCACAGGTAAACTATTTACAAGACTTTTCTTATCAAAGATCATTAAAATTTAGGCCAAAAGGGAAACCTTG
AGGGTGGTCATCTAAATGAATAGTGTACCATTCGCCTTACAGTGAGTTTGGTGTCCATTTGATAAATGTTCTGAAAAGAATAGTTTCTAGTAATTTTAAATCCGGTTTTCCCTTTGGAAC
  P  T  S  R  F  T  Y  H  M  V  S  G  M  S  L  K  P  Q  V  N  Y  L  Q  D  F  S  Y  Q  R  S  L  K  F  R  P  K  G  K  P  C>
_________________________________________________________"ENV"__________________________________________________________>

      6850      6860      6870      6880      6890      6900      6910      6920      6930      6940      6950      6960
CCCCAAAGAGATTTCCAGAGAATCGAAAGATTTAGTTTGGGAAGAATGTGTGGCCGATAGTGCAGTGATATTACAAAACAATACATTCGGAACAGTTATAGATTGGGCACCTAGAGGTCA
```

```
GGGGTTTCTCTAAAGGTCTCTTAGCTTTCTAAATCAAACCCTTCTTACACACCGGCTATCACGTCACTATAATGTTTTGTTATGTAAGCCTTGTCAATATCTAACCCGTGGATCTCCAGT
  P  K  E  I  S  R  E  S  K  D  L  V  W  E  E  C  V  A  D  S  A  V  I  L  Q  N  N  T  F  G  T  V  I  D  W  A  P  R  G  Q>
_________________________________________________________"ENV"__________________________________________________________>

      6970      6980      6990      7000      7010      7020      7030      7040      7050      7060      7070      7080
ATTCTACCACAATTGCACAGGACAAACTCAATTCTGTCCCAGTGCACTAGTGAGTCCAACTGTTGACAGTGATTTAACGGAAAATTTAGATAAACATAAGCACAAAAAATTACAGTCTTT
TAAGATGGTGTTAACGTGTCCTGTTTGAGTTAAGACAGGGTCACGTGATCACTCAGGTTGACAACTGTCACTAAATTGCCTTTTAAATCTATTTGTATTCGTGTTTTTTAATGTCAGAAA
  F  Y  H  N  C  T  G  Q  T  Q  F  C  P  S  A  L  V  S  P  T  V  D  S  D  L  T  E  N  L  D  K  H  K  H  K  K  L  Q  S  F>
_________________________________________________________"ENV"__________________________________________________________>

      7090      7100      7110      7120      7130      7140      7150      7160      7170      7180      7190      7200
CTACCCTTGGATATGGGGAGAAAAGGGAATGTCTACTCCAAGACCAAAAATGATAAGTCCTGTTTTTGGTCCTGAACATCCAGAATTATGGAGACTTACTGTGGCTTCATACCGCCTTAG
GATGGGAACCTATACCCCTCTTTTCCCTTACAGATGAGGTTCTGGTTTTTACTATTCAGGACAAAAACCAGGACTTGTAGGTCTTAATACCTCTGAATGACACCGAAGTATGGCGGAATC
  Y  P  W  I  W  G  E  K  G  M  S  T  P  R  P  K  M  I  S  P  V  F  G  P  E  H  P  E  L  W  R  L  T  V  A  S  Y  R  L  R>
_________________________________________________________"ENV"__________________________________________________________>

      7210      7220      7230      7240      7250      7260      7270      7280      7290      7300      7310      7320
AATTTGGTCTGGAAATCAAACTATAGAAACAAGAGATTATAAGCCATTTTACTCTATCAACCTAAATTCCAGTCTAACAGTTCCTTTACAAAGTTGTGTAAAGCCCCCTTATATGTTAGT
TTAAACCAGACCTTTAGTTTGATATCTTTGTTCTCTAATATTCGGTAAAATGAGATAGTTGGATTTAAGGTCAGATTGTCAAGGAAATGTTTCAACACATTTCGGGGGAATATACAATCA
  I  W  S  G  N  Q  T  I  E  T  R  D  Y  K  P  F  Y  S  I  N  L  N  S  S  L  T  V  P  L  Q  S  C  V  K  P  P  Y  M  L  V>
_________________________________________________________"ENV"__________________________________________________________>

      7330      7340      7350      7360      7370      7380      7390      7400      7410      7420      7430      7440
CATAGGAAATATAGTTATTAAACCAGACTCCCAAACTATAACTTGTGAAAATTGCAGATTGTTTACTTGCATTGATTCAACTTTTGATTGGCAGCACCGTATTCTACTGGTGAGAGCAAG
GTATCCTTTATATCAATAATTTGGTCTGAGGGTTTGATATTGAACACTTTTAACGTCTAACAAATGAACGTAACTAAGTTGAAAACTAACCGTCGTGGCATAAGATGACCACTCTCGTTC
  I  G  N  I  V  I  K  P  D  S  Q  T  I  T  C  E  N  C  R  L  F  T  C  I  D  S  T  F  D  W  Q  H  R  I  L  L  V  R  A  R>
_________________________________________________________"ENV"__________________________________________________________>

      7450      7460      7470      7480      7490      7500      7510      7520      7530      7540      7550      7560
AGAAGGCGTGTGGATCCCTGTGTCCATGGACCGACCATGGGAGGCCTCACCATCCATCCATATTTTGACTGAAGTATTAAAAGGCGTTTTAAGTAGATCCAAAAGATTCATTTTTACTTT
TCTTCCGCACACCTAGGGACACAGGTACCTGGCTGGTACCCTCCGGAGTGGTAGGTAGGTATAAAACTGACTTCATAATTTTCCGCAAAATTCATCTAGGTTTTCTAAGTAAAAATGAAA
  E  G  V  W  I  P  V  S  M  D  R  P  W  E  A  S  P  S  I  H  I  L  T  E  V  L  K  G  V  L  S  R  S  K  R  F  I  F  T  L>
_________________________________________________________"ENV"__________________________________________________________>

      7570      7580      7590      7600      7610      7620      7630      7640      7650      7660      7670      7680
AATTGCAGTGATTATGGGATTAATTGCAGTCACAGCTACAGCCTCTGTGGCAGGAGTTGCATTGCACTCTTCTGTTCAGACAGTAAGCTTTGTTGACAATTGGCAAAAGAATTCCACAAG
TTAACGTCACTAATACCCTAATTAACGTCAGTGTCGATGTCGGAGACACCGTCCTCAACGTAACGTGAGAAGACAAGTCTGTCATTCGAAACAACTGTTAACCGTTTTCTTAAGGTGTTC
  I  A  V  I  M  G  L  I  A  V  T  A  T  A  S  V  A  G  V  A  L  H  S  S  V  Q  T  V  S  F  V  D  N  W  Q  K  N  S  T  R>
_________________________________________________________"ENV"__________________________________________________________>

      7690      7700      7710      7720      7730      7740      7750      7760      7770      7780      7790      7800
GTTGTGGAATTCACAATCTGGTATCGATCAAAAATTGGCAAATCAAATTAATGATCTTAGACAAACCGTCATTTGGATGGGAGATAGACTCATGAGCTTGGAACATCGTTTCCAGTTACA
CAACACCTTAAGTGTTAGACCATAGCTAGTTTTTAACCGTTTAGTTTAATTACTAGAATCTGTTTGGCAGTAAACCTACCCTCTATCTGAGTACTCGAACCTTGTAGCAAAGGTCAATGT
  L  W  N  S  Q  S  G  I  D  Q  K  L  A  N  Q  I  N  D  L  R  Q  T  V  I  W  M  G  D  R  L  M  S  L  E  H  R  F  Q  L  Q>
_________________________________________________________"ENV"__________________________________________________________>

      7810      7820      7830      7840      7850      7860      7870      7880      7890      7900      7910      7920
GTGTGACTGGAATACGTCAGATTTTTGTATTACACCCCAAGTTTATAATGAGTCTAAACATCACTGGGACATGGTTAGACGCCATCTACAGGGAAGAGAAGATAATCTCACTTTAGACAT
CACACTGACCTTATGCAGTCTAAAAACATAATGTGGGGTTCAAATATTACTCAGATTTGTAGTGACCCTGTACCAATCTGCGGTAGATGTCCCTTCTCTTCTATTAGAGTGAAATCTGTA
  C  D  W  N  T  S  D  F  C  I  T  P  Q  V  Y  N  E  S  K  H  H  W  D  M  V  R  R  H  L  Q  G  R  E  D  N  L  T  L  D  I>
_________________________________________________________"ENV"__________________________________________________________>

      7930      7940      7950      7960      7970      7980      7990      8000      8010      8020      8030      8040
TTCTAAATTAAAAGAACAAATTTTTGAAGCCTCTCAAAGCCACTTAAATATTGTGCCTGGAGCTGAGGCATTAGATCAAGTGGCAAAAAATCTTTATGAATTAAACCCCACGACTTGGAT
AAGATTTAATTTTCTTGTTTAAAAACTTCGGAGAGTTTCGGTGAATTTATAACACGGACCTCGACTCCGTAATCTAGTTCACCGTTTTTTAGAAATACTTAATTTGGGGTGCTGAACCTA
  S  K  L  K  E  Q  I  F  E  A  S  Q  S  H  L  N  I  V  P  G  A  E  A  L  D  Q  V  A  K  N  L  Y  E  L  N  P  T  T  W  I>
_________________________________________________________"ENV"__________________________________________________________>

      8050      8060      8070      8080      8090      8100      8110      8120      8130      8140      8150      8160
TAAGTCTATTGGAAACTCTACTGCAATAAATTTTGGAATTATGTGTCTCTGTTTAATCAGCTTGTTTTTAGTGTGCTGGACCAGTCGAAGAATCCTGCGTCAAAATCGAGAGAACGAACA
ATTCAGATAACCTTTGAGATGACGTTATTTAAAACCTTAATACACAGAGACAAATTAGTCGAACAAAAATCACACGACCTGGTCAGCTTCTTAGGACGCAGTTTTAGCTCTCTTGCTTGT
  K  S  I  G  N  S  T  A  I  N  F  G  I  M  C  L  C  L  I  S  L  F  L  V  C  W  T  S  R  R  I  L  R  Q  N  R  E  N  E  Q>
_________________________________________________________"ENV"__________________________________________________________>

      8170      8180      8190      8200      8210      8220      8230      8240      8250      8260      8270      8280
AGCCTTCATCGCCATGGCACATTTATATAGAGGAAAAGGGAGGGAGAACGTTGCGGGAAGTCAGGGACCTTGAACGCAGGGACTGGCTGAAGCCACGGCAGAAAAACATAAAATGTAAAG
TCGGAAGTAGCGGTACCGTGTAAATATATCTCCTTTTCCCTCCCTCTTGCAACGCCCTTCAGTCCCTGGAACTTGCGTCCCTGACCGACTTCGGTGCCGTCTTTTTGTATTTTACATTTC
  A  F  I  A  M  A  H  L  Y  R  G  K  G  R  E  N  V  A  G  S  Q  G  P  *>
________________________________"ENV"________________________________________>

      8290      8300      8310      8320      8330      8340      8350      8360      8370      8380      8390      8400
ATTTCATGGACATTTATTAGTTCTCCAAAATTAATACTTTTGTAATTTCTTATGTCTGTCTTTACTTTAATCTTTTAATTCCATCATCTTCTTTGTAAACTGAGGAGGATATATGTCACC
TAAAGTACCTGTAAATAATCAAGAGGTTTTAATTATGAAAACATTAAAGAATACAGACAGAAATGAAATTAGAAAATTAAGGTAGTAGAAGAAACATTTGACTCCTCCTATATACAGTGG

      8410      8420      8430      8440      8450      8460      8470      8480      8490      8500      8510      8520
TCCGGACCCTGTGATGATTGCCTTAACTGCACAAATTGTTTGTGGAGCATGTGTGTTTGAACAATATGAAATCTGGGCATCTTGGAAAAAGAATAAGATAACAGCAATGTTCAGGGAACA
AGGCCTGGGACACTACTAACGGAATTGACGTGTTTAACAAACACCTCGTACACACAAACTTGTTATACTTTAGACCCGTAGAACCTTTTTCTTATTCTATTGTCGTTACAAGTCCCTTGT

      8530      8540      8550      8560      8570      8580      8590      8600      8610      8620      8630      8640
AGGGAGGCAACCTTGAACTGGCCGCCGGTGAGCCGGACGGAACAGAGCCATATTTCTTCTCTTTTCATTATGCAAATAGGAGAAATATCGCTGAATTCTTTTTCTCAGCAAGGAACACCC
TCCCTCCGTTGGAACTTGACCGGCGGCCACTCGGCCTGCCTTGTCTCGGTATAAAGAAGAGAAAAGTAATACGTTTATCCTCTTTATAGCGACTTAAGAAAAAGAGTCGTTCCTTGTGGG

      8650      8660      8670      8680      8690      8700      8710      8720      8730      8740      8750      8760
CTGAGAAAGAGAATGCGCTCTGAGGGTAGGCCTATAAACGACCCCCTTGGAGGCGTGCCGCCTTTTACGGTTGAAGCCGAAGGGATGAAATAAGCCCCGGCCTCCTGTAGCGCTCCCAGG
GACTCTTTCTCTTACGCGAGACTCCCATCCGGATATTTGCTGGGGGAACCTCCGCACGGCGGAAAATGCCAACTTCGGCTTCCCTACTTTATTCGGGGCCGGAGGACATCGCGAGGGTCC

      8770      8780      8790      8800      8810      8820      8830      8840      8850      8860      8870      8880
CTTATTAGGACGAGGAAATTCCCACCTAATAAATTTTGGTCAGACAGGTTGTCTGCTCTTAAACCCTGTTTTCTGACAAGATGTTATCCATGACTATGCATGCCCCAAAATTTTAATTTT
GAATAATCCTGCTCCTTTAAGGGTGGATTATTTAAAACCAGTCTGTCCAACAGACGAGAATTTGGGACAAAAGACTGTTCTACAATAGGTACTGATACGTACGGGGTTTTAAAATTAAAA

      8890      8900      8910      8920      8930      8940      8950      8960      8970      8980      8990      9000
```

```
AACACCATCCTGTGATCTCATCCTGCCTCCACTTGCTTTGTGATATTCCGTTACTTTGTGAAGTATGTAATCTCGGTGTCATGATGACAATGGTGGTTTTGTTGAAAAGAAAAGGGGGAA
TTGTGGTAGGACACTAGAGTAGGACGGAGGTGAACGAAACACTATAAGGCAATGAAACACTTCATACATTAGAGCCACAGTACTACTGTTACCACCAAAACAACTTTTCTTTTCCCCCTT

      9010      9020      9030      9040      9050      9060      9070      9080      9090      9100      9110      9120
ATGTGGGGAAAAGAAAGAGATCAGCCTGTTACTGTGTCTATATAGAAAGAAGTAGACATAAGAGACTCCATCTTGTTCTGTATTTGAGATGCTGTTAATCTGTGACCCTACCCCCAACCT
TACACCCCTTTTCTTTCTCTAGTCGGACAATGACACAGATATATCTTTCTTCATCTGTATTCTCTGAGGTAGAACAAGACATAAACTCTACGACAATTAGACACTGGGATGGGGGTTGGA
 ________________________________________________________"3'LTR"_________________________________________________________>

      9130      9140      9150      9160      9170      9180      9190      9200      9210      9220      9230      9240
TGTCCTTGCAAGAGACATGTGCTGTGGTAACTCAAGGTTTAATGGATTTTGGGCGTGCAGGGTGTGACTTTGTTCCTAGAAAAGCTAGGTATTGTCCAAGGTTTATCCCCATGTGATAGG
ACAGGAACGTTCTCTGTACACGACACCATTGAGTTCCAAATTACCTAAAACCCGCACGTCCCACACTGAAACAAGGATCTTTTCGATCCATAACAGGTTCCAAATAGGGGTACACTATCC
_________________________________________________________"3'LTR"_________________________________________________________>

      9250      9260      9270      9280      9290      9300      9310      9320      9330      9340      9350      9360
ATGAAACAATGTTGCTAAAAGGTTTATCTCAAGGCACAGGATTTACCTTTAAACTTATTCATGTCACAGATCCTTGTTCTTATGTCTTACTGCTAATTTCCTCCCTAAAAATGATCCTAT
TACTTTGTTACAACGATTTTCCAAATAGAGTTCCGTGTCCTAAATGGAAATTTGAATAAGTACAGTGTCTAGGAACAAGAATACAGAATGACGATTAAAGGAGGGATTTTTACTAGGATA
_________________________________________________________"3'LTR"_________________________________________________________>

      9370      9380      9390      9400      9410      9420      9430      9440      9450      9460      9470      9480
TGTCCTGCCACTCCCTTATCTTTAAGATGGTAAAGATAATTATCTATAAATACTAAGGGAACTCAGAGGCCGGTGCCGGCGTGGGTCCTCTGTAAGCTGAGCGCCGGTCCCCTGGCCCC
ACAGGACGGTGAGGGAATAGAAATTCTACCATTTCTATTAATAGATATTTATGATTCCCTTGAGTCTCCGGCCACGGCCGCACCCAGGAGACATTCGACTCGCGGCCAGGGGACCCGGGG
_________________________________________________________"3'LTR"_________________________________________________________>

      9490      9500      9510      9520      9530      9540      9550      9560      9570      9580
CGCTTTTCTTTCTCTATACTTTGTCTCTGTGTCTTATTTCTTTTCTCAAGTCTCTCGTTCCACCTTACGAGAAACACCCACAGGTGTGGAGGGGCAGGCCACCCCTTCA
GCGAAAAGAAAGAGATATGAAACAGAGACACAGAATAAAGAAAAGAGTTCAGAGAGCAAGGTGGAATGCTCTTTGTGGGTGTCCACACCTCCCCGTCCGGTGGGGAAGT
___________________________________________________"3'LTR"_____________________________________________________>
```
