# Supplementary material for: Identification and spontaneous immune targeting of an endogenous retrovirus K envelope protein in the Indian rhesus macaque model of human disease
Source: Retrovirology. 2016 Jan 15;13:6. doi: 10.1186/s12977-016-0238-0 (PMC4714462; doi:10.1186/s12977-016-0238-0)
Supplement: Supplementary file 2 — 10.1186/s12977-016-0238-0 Figure S2. Alignment of captured SERV-K1 Env mRNA to SERV-K1 provirus. Protein alignment of the predicted amino acid sequence of chromosome 12 provirus and two mRNA variants captured from rhesus macaque r02120 is shown. Matches at each amino acid position are indicated with asterisks (*), while mismatches are indicated with periods (.). Mismatches in each sequence are highlighted in yellow. [file 12977_2016_238_MOESM2_ESM.pdf]

Figure S2. Alignment of captured SERV-K1 Env mRNA to SERV-K1 provirus. Protein alignment of the predicted amino acid sequence of chromosome 12 provirus and two mRNA variants captured from rhesus macaque r02120 is shown. Matches at each amino acid position are indicated with asterisks (*), while mismatches are indicated with periods (.). Mismatches in each sequence are highlighted in yellow.

```
r02120 provirus (12)   1 MNPSEMQRKAPPRRQKHRNRAPLTRMMNQVMISEEQMKSPRTKKAELPTWAQLKKLTPLA  60
r02120 Env mRNA v1     1 MNPSEMQRKAPPRRQKHRNRAPLTRMMNQVMISEEQMKSPRTKKAELPTWAQLKKLTPLA  60
r02120 Env mRNA v2     1                                                               0


r02120 provirus (12)  61 GKSLASTKVTQTPEKMLLTALMIVSTVVSLPMPAGAAAANYTYWAYVPFPPLIRAVTWMD 120
r02120 Env mRNA v1    61 GKSLASTKVTQTPEKMLLTALMIVSTVVSLPMPAGAAAANYTYWAYVPFPPLIRAVTWMD 120
r02120 Env mRNA v2     1                               PMPAGAAAANYTYWAYVPFPPLIRAVTWMD  30
                                                       ******************************

r02120 provirus (12) 121 NPIEVYVNNSVWVPGPTDDRCPAKPEEEGMMINISIGYRYPPICLGRAPGCLMPAIQNWL 180
r02120 Env mRNA v1   121 NPIEVYVNNSVWVPGPTDDRCPAKPEEEGMMINISIGYRYPPICLGRAPGCLMPAIQNWL 180
r02120 Env mRNA v2    31 NPIEVYVNNSVWVPGPTDDRCPAKPEEEGMMINISIGYRYPPICLGRAPGCLMPAIQNWL  90
                         ************************************************************

r02120 provirus (12) 181 VEVPTVSPTSRFTYHMVSGMSLKPQVNYLQDFSYQRSLKFRPKGKPCPKEISRESKDLVW 240
r02120 Env mRNA v1   181 VEVPTVSPTSRFTYHMVSGMSLKPQVNYLQDFSYQRSLKFRPKGKPCPKEISRESKDLVW 240
r02120 Env mRNA v2    91 VEVPTVSPTSRFTYHMVSGMSLKPQVNYLQDFSYQRSLKFRPKGKPCPKEISRESKDLVW 150
                         ************************************************************

r02120 provirus (12) 241 EECVADSAVILQNNTFGTVIDWAPRGQFYHNCTGQTQFCPSALVSPTVDSDLTENLDKHK 300
r02120 Env mRNA v1   241 EECVADSAVILQNNTFGTVIDWAPRGQFYHNCTGQTQFCPSALVSPTVDSDLTENLDKHK 300
r02120 Env mRNA v2   151 EECVADSAVILQNNTFGTVIDWAPRGQFYHNCTGQTQFCPSALVSPTVDSDLTENLDKHK 210
                         ************************************************************

r02120 provirus (12) 301 HKKLQSFYPWIWGEKGMSTPRPKMISPVFGPEHPELWRLTVASYRLRIWSGNQTIETRDY 360
r02120 Env mRNA v1   301 HKKLQSFYPWIWGEKGISTPRPKMISPVFGPEHPELWRLTVASYRLRIWSGNQTIETRDY 360
r02120 Env mRNA v2   211 HKKLQSFYPWIWGEKGMSTPRPKMISPVFGPEHPELWRLTVASYRLRIWSGNQTIETRDY 270
                         ****************.*******************************************

r02120 provirus (12) 361 KPFYSINLNSSLTVPLQSCVKPPYMLVIGNIVIKPDSQTITCENCRLFTCIDSTFDWQHR 420
r02120 Env mRNA v1   361 KPFYSINLNSSLTVPLQSCVKPPYMLVIGNIVIKPDSQTITCENCRLFTCIDSTFDWQHR 420
r02120 Env mRNA v2   271 KPFYSINLNSSLTVPLQSCVKPPYMLVIGNIVIKPDSQTITCENCRLFTCIDSTFDWQHR 330
                         ************************************************************

r02120 provirus (12) 421 ILLVRAREGVWIPVSMDRPWEASPSIHILTEVLKGVLSRSKRFIFTLIAVIMGLIAVTAT 480
r02120 Env mRNA v1   421 ILLVRAREGVWIPVSMDRPWEASPSIHILTEVLKGVLSRSKRFIFTLIAVIMGLIAVTAT 480
r02120 Env mRNA v2   331 ILLVRAREGVWIPVSMDRPWEASPSIHILTEVLKGVLSRSKRFIFTLIAVIMGLIAVTAT 390
                         ************************************************************

r02120 provirus (12) 481 ASVAGVALHSSVQTVSFVDNWQKNSTRLWNSQSGIDQKLANQINDLRQTVIWMGDRLMSL 540
r02120 Env mRNA v1   481 ATVAGVALHSSVQTVSFVDNWQKNSTRLWNSQSGIDQKLANQINDLRQTVIWMGDRLMSL 540
r02120 Env mRNA v2   391 ASVAGVALHSSVQTVSFVDNWQKNSTRLWNSQSGIDQKLANQINDLRQTVIWMGDRLMSL 450
                         *.**********************************************************

r02120 provirus (12) 541 EHRFQLQCDWNTSDFCITPQVYNESKHHWDMVRRHLQGREDNLTLDISKLKEQIFEASQS 600
r02120 Env mRNA v1   541 EHRFQLQCDWNTSDFCIIPQVYNESKHHWDMVRRHLQGREDNLTLDISKLKEQIFEASQS 600
r02120 Env mRNA v2   451 EHRFQLQCDWNTSDFCITPQVYNESKHHWDMVRRHLQGREDNLTLDISKLKEQIFEASQS 510
                         ***************** ******************************************

r02120 provirus (12) 601 HLNIVPGAEALDQVAKNLYELNPTTWIKSIGNSTAINFGIMCLCLISLFLVCWTSRRILR 660
r02120 Env mRNA v1   601 HLNIVPGAEALDQVAKNLYELNPTTWIKSIGNSTAINFGIMCLCLIGLFLVCWTSRRILR 660
r02120 Env mRNA v2   511 HLNIVPGAEALDQVAKNLYELNPTTWIKSIGNSTAINFGIMCLCLISLFLVCWTSRRILR 570
                         **********************************************.*************

r02120 provirus (12) 661 QNRENEQAFIAMAHLYRGKGRENVAGSQGP 690
r02120 Env mRNA v1   661 QNRENEQAFIAMAHLYRGKGRENVAGSQGP 690
r02120 Env mRNA v2   571 QNRENEQAFIAMAHLYRGKGRENVAGSQGP 600
                         ******************************
```
