# Supplementary material for: Identification and spontaneous immune targeting of an endogenous retrovirus K envelope protein in the Indian rhesus macaque model of human disease
Source: Retrovirology. 2016 Jan 15;13:6. doi: 10.1186/s12977-016-0238-0 (PMC4714462; doi:10.1186/s12977-016-0238-0)
Supplement: Supplementary file 3 — 10.1186/s12977-016-0238-0 Figure S3. Alignment of SERV-K to primate ERVs. Nucleotide alignment showing part of the pol locus of SERV-K from rhesus macaque r02120 aligned to various ERVs from hominids (humans, chimpanzees) and old world monkeys. Matches at each amino acid position are indicated with asterisks (*). The SERV-K1 locus described here is highlighted in grey. CERV = chimpanzee endogenous retrovirus; HERV = human endogenous retrovirus. Sequences shown here were used to construct phylogram in Fig. 1d. [file 12977_2016_238_MOESM3_ESM.pdf]

Figure S3. Alignment of SERV-K to primate ERVs. Nucleotide alignment showing part of the *pol* locus of SERV-K from rhesus macaque r02120 aligned to various ERVs from hominids (humans, chimpanzees) and old world monkeys. Matches at each amino acid position are indicated with asterisks (*). The SERV-K1 locus described here is highlighted in grey. CERV = chimpanzee endogenous retrovirus; HERV = human endogenous retrovirus. Sequences shown here were used to construct phylogram in Figure 1d.

```
CERV-KOLD35587     1 GAGGCTTTACACTTATTAGCAAAGGAACAATTAGAAAAGGGACATATTGAGCCTTCATTTTCGCCTTGGAATTCTCCTGTGTTTGTAATTCAGAAAAAATCCGGCAGATGGCGCATGCTG  120
HERV-KOLD35587     1 GAGGCTTTACACTTATTAGCAAAGGAACAATTAGAAAAGGGACATATTGAGCCTTCATTTTCGCCTTGGAATTCTCCTGTGTTTGTAATTCAGAAAAAATCCGGCAGATGGCGCGTGCTG  120
SERV-K1 chr.11     1 GAGGCTTTACATTTATTAGCAAAGGAACAATTAGAAAAGGGACACATTGAGCCTTCATTCTCGCCCTGAAATTCTCCTGTATTTGTAATTCAGAAAAAATCAGGCAAATGGCATATGTTA  120
Guereza            1 GAGGCTTTACACTTATTAGCAAAGGAACAATTAGAAAAGGGACACATTGTGCCTTCATTCTCACCCTGGAATTCTCCTGTATTTGTAATTCAGAAAAAATCAGGCAAATGGCGTATGTTA  120
SERV-K1 chr.12     1 GAGGCTTTACATTCATTAGCAAAGGAACAATTTAAAAAAGGACACATTGAGCCTTCATTCTCACCCTGGAATTCTCCTGTATTTGTAATACAGAAAAAATCAGGCAAATGGCGTATGTTA  120
Grivet             1 GAGGCTTTACATTCATTAGCAAAGGAACAATTAGAAAAGGGACACATTGAGCCTTCATTCTCACCCTGGAATTCTCCTGTATTTGTAATTAAGAAAAAATCAGGCAAGTGGCGTATGTTA  120
SERV-K1 chr.5      1 GAGGCTTTACATTTATTAGCAAAGGAACAGTCAGAAAAGGGACACACTGAGGCATCATTTTCGCCCTGGAATTCTCCTGTATTTGTAATTCAGAAAAAATCAGGCAGATGGCGTATGTTA  120
CERV-K60           1 GAGGCTTTACATTTATTAGCAAATGAACAGTTAGAAAAGCGACATATTGAGCCTTCATTCTCGCCTTGGAATTCTCCTGTGTTTGTAATTCAGAAGAAATCCAGCAAATGGCGTATGTTA  120
CERV-K102          1 GAGGCTTTACATTTATTAGCAAATGAACAGTTAGAAAAGGGTCATATTGAGCCTTCATTCTCGCCTTGGAATTCTCCTGTGTTTGTAATTCAGAAGAAATCAGGCAAATGGCGTATGTTA  120
HERV-K104          1 GAGGCTTTACATTTATTAGCAAATGAACAGTTAGAAAAGGGTCATATTGAGCCTTCATTCTCGCCTTGGAATTCTCCTGTGTTTGTGATTCAGAAGAAATCAGGCAAATGGCGTATGTTA  120
HERV-K50D          1 GAGGCTTTACATTTATTAGCAAATGAACAGTTAGAAAAGGGTCATATTGAGCCTTCATTCTCGCCTTGGAATTCTCCTGTGTTTGTGATTCAGAAGAAATCAGGCAAATGGCGTATGTTA  120
HERV-K50B          1 GAGGCTTTACATTTATTAGCAAATGAACAGTTAGAAAAGGGTCATATTGAGCCTTCATTCTCGCCTTGGAATTCTCCTGTGTTTGTAATTCAGAAGAAATCAGGCAAATGGCGTATGTTA  120
HERV-K106          1 GAGGCTTTACATTTATTAGCAAATGAACAGTTAGAAAAGGGTCATATTGAGCCTTCATCCTCGCCTTGGAATTCTCCTGTGTTTGTAATTCAGAAGAAATCAGGCAAATGGCGTATGTTA  120
HERV-K101          1 GAGGCTTTACATTTATTAGCAAATGAACAGTTAGAAAAGGGTCATATTGAGCCTTCGTTCTCACCTTGGAATTCTCCTGTGTTTGTAATTCAGAAGAAATCAGGCAAATGGCGTATGTTA  120
HERV-K103          1 GAGGCTTTACATTTATTAGCAAATGAACAGTTAGAAAAGGGTCACATTGAGCCTTCGTTCTCACCTTGGAATTCTCCTGTGTTTGTAATTCAGAAGAAATCAGGCAAATGGCGTATGTTA  120
CERV-K100          1 GAGGCTTTACATTTATTAGCAAATGAACAGTTAGAAAAGGGTCACATTGAGCCTTCGTTCTCACCTTGGAATTCTCCTGTGTTTGTAATTCAGAAGAAATCAGGCAAATGGCATACGTTA  120
HERV-K10           1 GAGGCTTTACATTTATTAGCAAATGAACAGTTAGAAAAGGGTCACATTGAGCCTTCGTTCTCACCTTGGAATTCTCCTGTGTTTGTAATTCAGAAGAAATCAGGCAAATGGCATACGTTA  120
HERV-K108          1 GAGGCTTTACATTTATTAGCAAATGAACAGTTAGAAAAGGGTCATATTGAGCCTTCGTTCTCACCTTGGAATTCTCCTGTGTTTGTAATTCAGAAGAAATCAGGCAAATGGCGTATGTTA  120
HERV-K109          1 GAGGCTTTACATTTATTAGCAAATGAACAGTTAGAAAAGGGTCATATTGAGCCTTCGTTCTCACCTTGGAATTCTCCTGTGTTTGTAATTCAGAAGAAATCAGGCAAATGGCGTATGTTA  120
HERV-K113          1 GAGGCTTTACATTTATTAGCAAATGAACAGTTAGAAAAGGGTCACATTGAACCTTCGTTCTCACCTTGGAATTCTCCTGTGTTTGTAATTCAGAAGAAATCAGGCAAATGGCGTATGTTA  120
HERV-K36           1 GAGGCTTTACATTTATTAGCAAATGAACAGTTAGAAAAGGGTCATATTGAGCCTTCGTTCTCACCTTGGAATTCTCCTGTGTTTGTAATTCAGAAGAAATCAGGCAAATGGCGTATGTTA  120
HERV-K115          1 GAGGCTTTACATTTATTAGCAAATGAACAGTTAGAAAAGGGTCATATTGAGCCTTCGTTCTCACCTTGGAATTCTCCTGTGTTTGTAATTCAGAAGAAATCAGGCAAATGGCGTATGTTA  120
HERV-K102          1 GAGGCTTTACATTTATTAGCAAATGAACAGTTAGAAAAGGGTCACATTGAGCCTTCGTTCTCACCTTGGAATTCTCCTGTGTTTGTAATTCAGAAGAAATCAGGCAAATGGCGTATGTTA  120
HERV-K50A          1 GAGGCTTTACATTTATTAGCAAATGAACAGTTAGAAAAGGGTCACATTGAGCCTTCGTTCTCACCTTGGAATTCTCCTGTGTTTGTAATTCAGAAGAAATCAGGCAAATGGCGTATGTTA  120
                     *********** * ********* ***** *    ****  * ** * **    * ** *  ** ** ** *********** ***** **  **** *****  ***  ****    * *

CERV-KOLD35587   121 ACCGACTTAAGAGCCGTTAATGCTGTAATTCAACCCATGGGGGCTCTCCAACCTGGGTTGCTGTCTCCGGTCATGATCCCCAAAGATCGGCCTTTAGTTATAATTGATCTGAAGGATTGC  240
HERV-KOLD35587   121 ACCGACTTAAGAGCCGTTAATGCTGTAATTCAACCCATGGGGGCTCTCCAACCTGGGTTGCTCTCTCCGGTCATGATCCCCAAAGATCGGCCTTTAGTTATAATTGATCTGAAGGATTGC  240
SERV-K1 chr.11   121 ATGGACTTAAGAGCCGTAAATGCCGTAATTCAACCCACGGGGCCTCTTCAACCCGGACTGCCCTCTCCGGCCATGATCCCAAAAGACTGGCCTTTAATTATAATTGATCTTAAGGATTGC  240
Guereza          121 ATGGACTTAAGAGCCATAAACGCTGTAATTCAACCCATGGGGCCTCTCCAACCTGGATTGCCCTCTCCGGCCATGATCCCAAAAGACTGGCCTTTAATTATAATTGATCTTAAGGATTGT  240
SERV-K1 chr.12   121 ATGGACTTAAGAGCCGTAAATGCTGTAATTCAACCCATGGGGCCTCTTCAACCCGGATTGCCCTCTCCGGCCATGATCCCAAAAGACTGGCCTTTAATTATAATTGATCTTAAGGATTGC  240
Grivet           121 ATGGACTTAAGAGCCGTAAATGCTGTAATTCAACCCATGGGGCCTCTTCAACCCGGATTGCCCTCTCCGGCCATGATCCCAAAAGACTGGCCTTTAATTATAATTGATCTTAAGGACTGC  240
SERV-K1 chr.5    121 ACCGACTTAAGAGCCATAAATGCTGTAATTCAACCCATGGGGCCTCTTCAACCCGGATTGCCCTCCCCAGCCACGATTCCAAAAGACTGGCCTTTAATTCTAATTGATCTGAAGGACTGC  240
CERV-K60         121 ACTGACTTAAGGGCTGTAAATGCTGTAATTCAACCCATGGGGCCTCTCCAACCTGGGTTGCCCTCTCCGGCCATGATCCCAAAAGATTGGCCTTTAATTATAATTGATCTAAAGGACTGC  240
CERV-K102        121 ACTGACTTTAGGGCCGTAAACGCCGTAATTCAACCCATGGGGCCTCTCCAACCTGGGTTGCCCTCTCTGGCCATGATCCCAAAAGACTGGCCTTTAATTATAATTGATCTAAAGGATTGC  240
HERV-K104        121 ACTGACTTAAGGGCCGTAAACGCCATAATTCAACCCATGGGGCCTCTCCAATCTAGGTTGCCCTCTCCGGCCATGATCCCAAAAGATTGGCCTTTAATTATAATTGATCTAAAGGATTGC  240
HERV-K50D        121 ACTGACTTAAGGGCCGTAAACGCCATAATTCAACCCATGGGGCCTCTCCAATCTGGGTTGCCCTCTCCGGCCATGATCCCAAAAGATTGGCCTTTAATTATAATTGATCTAAAGGATTGC  240
HERV-K50B        121 ACTGACTTAAGGGCCGTAAACGCCGTAATTCAACCCATGGGGCCTCTCCAACCCGGGTTGCCCTCTCCGGCCATGATCCCAAAAGATTGGCCTTTAATTATAATTGATCTAAAGGATTGC  240
HERV-K106        121 ACTAACTTAAGGGCCGTAAACGCCGTAATTCAACCCATGGGGCCTCTCCAACCCGGGTTGCCCTCTCCGGCCATGATCCCAAAAGATTGGCCTTTAATTATAATTGATCTAAAGGATTGC  240
HERV-K101        121 ACTGACTTAAGGGCCGTAAACGCCGTAATTCAACCCATGGGGCCTCTCCAACCCGGGTTGCCCTCTCCGGCCATGATCCCAAAAGATTGGCCTTTAATTATAATTGATCTAAAGGATTGC  240
HERV-K103        121 ACTGACTTAAGGGCTGTAAACGCCTTAATTCAACCCATGGGGCCTCTCCAACCCGGGTTGCCCTCTCCGGCCATGATCCCAAAAGATTGGCCTTTAATTATAATTGATCTAAAGGACTGC  240
CERV-K100        121 ACTGACTTAAGGGCTGTAAACGCCGTAATTCAACCCATGGGGCCTCTCCAACCCGGGTTGCCCTCTCCGGCCATGATCCCAAAAGATTGGCCTTTAATTATAATTGATCTAAAGGATTGC  240
HERV-K10         121 ACTGACTTAAGGGCTGTAAACGCCGTAATTCAACCCATGGGGCCTCTCCAACCCGGGTTGCCCTCTCCGGCCATGATCCCAAAAGATTGGCCTTTAATTATAATTGATCTAAAGGATTGC  240
HERV-K108        121 ACTGACTTAAGGGCTGTAAACGCCGTAATTCAACCCATGGGGCCTCTCCAACCCGGGTTGCCCTCTCCGGCCATGATCCCAAAAGATTGGCCTTTAATTATAATTGATCTAAAGGATTGC  240
HERV-K109        121 ACTGACTTAAGGGCTGTAAACGCCGTAATTCAACCCATGGGGCCTCTCCAACCCGGGTTGCCCTCTCCGGCCATGATCCCAAAAGATTGGCCTTTAATTATAATTGATCTAAAGGATTGC  240
HERV-K113        121 ACTGACTTAAGGGCTGTAAACGCCGTAATTCAACCCATGGGGCCTCTCCAACCTGGGTTGCCCTCTCCAGCCATGATCCCAAAAGATTGGCCTTTAATTATAATTGATCTAAAGGATTGC  240
HERV-K36         121 ACTGACTTAAGGGCTGTAAACGCCGTAATTCAACCCATGGGGCCTCTCCAACCTGGGTTGCCATCTCCAGCCATGATCCCAAAAGATTGGCCTTTAATTATAATTGATCTAAAGGATTGC  240
HERV-K115        121 ACTGACTTAAGGGCTGTAAACGCCGTAATTCAACCCATGGGGCCTCTCCAACCCGGGTTGCCCTCTCCAGCCATGATCCCAAAAGATTGGCCTTTAATTATAATTGATCTAAAGGATTGC  240
HERV-K102        121 ACTGACTTAAGGGCTGTAAACGCCGTAATTCAACCCATGGGGCCTCTCCAACCTGGGTTGCCCTCTCCAGCCATGATCCCAAAAGATTGGCCTTTAATTATAATTGATCTAAAGGATTGC  240
HERV-K50A        121 ACTGACTTAAGGGCTGTAAACGCCGTAATTCAACCCATGGGGCCTCTCCAACCTGGGTTGCCCTCTCCAGCCATGATCCCAAAAGATTGGCCTTTAATTATAATTGATCTAAAGGATTGC  240
                     *   **** ** **  * ** **  *********** **** **** *** *  *  ***  ** *  * ** *** ** *****  ******** ** ********** ***** **

CERV-KOLD35587   241 TTTTTTACCGTTCCTCTGGCAAAACAGGATTTTGAAAAATTTGCTTTTACTATACCAGCCATAAATAATAAAGAACCAGCCACCAGGTTTCAGTGGAAAGTGTTGCCTCAGGGAATGCTT  360
HERV-KOLD35587   241 TTTTTTACCGTTCCTCTGGCAAAACAGGATTTTGAAAAATTTGCTTTTACTATACCAGCCATAAATAATAAAGAACCAGCCACCAGGTTTCAGTGGAAAGTGTTGCCTCAGGGAATGCTT  360
SERV-K1 chr.11   241 TTTTTTACCATTCCTCTGGCAGAGCAGGATTGTGAAAAATTTGCCTTTACTATACCAGCCATAAAAAATAAAGAACCAGCCACCAGGTTTCAGTGGAAAGTATTACCTCAGGGAATGCTT  360
Guereza          241 TTTTTTACCATTCCTCTGGCAGAGCAAGATTGTGAAAAATTTGCCTTTACTATACCAGCCATAAATAATAAAGAACCAGCCACCAGGTTTCAGTGGAAAGTTTTACCTCAAGGAATGCTT  360
SERV-K1 chr.12   241 TTTTTTACCATTCCTCTGGCAGAGCAAGATTGTGAAAAATTTGCCTTTACTATACCAGCCATAAATAATAAAGAACCAGCCACCAGGTTCCAGTGGAAAGTACTACCTCAGGGAATGCTT  360
Grivet           241 TTTTTTACCATTCCTCTGGCAGAGCAAGATTGTGAAAAATTTGCCTTTACTATACCAGCCATAAATAATAAAGAACCAGCCACCAGATTTCAGTGGAAAGTACTACCTCAGGGAATGCTT  360
SERV-K1 chr.5    241 TTTTTTACCATTCCTCTGGCAGAGCAGGATTGTGAAAAATTTGCTTTTACTATACCAGCCGTAAATAATAAAGAACCAGCCACCAGGTTTCAGTGGAAAGTGTTACCTCGGGGAATGCTT  360
CERV-K60         241 TTTTTTACCATCCCTCTGGCGGAGCCGGATTGCGAAAAATTTGCCTTTACTATACCAGCCATAAATAATAAAGAACCAGCCACCAGGTTTCAGTGGAAAGTGTTACCTCAGGGAATGCTT  360
CERV-K102        241 TTTTTTACCATCCCTCTGGCGGAGCAGGATTGCGAAAAATTTGCCTTTACTATACCAGCCATAAATAATAAAGAACCAGCCACCAGGTTTCAGTGGAAAGTGTTACCTCAGGGAATGCTT  360
HERV-K104        241 TTTTTTACCATCCCTCTGGCGGAGCAGGATTGCGAAAAATTTGCCTTTACTGTACTAGCCATAAATAATAAAGAACCAGCCACCAGGTTTCAGTGGAAAGTGTTACCTCAGGGAATGCTT  360
HERV-K50D        241 TTTTTTACCATCCCTCTGGCGGAGCAGGATTGCGAAAAATTTGCCTTTACTATACTAGCCATAAATAATAAAGAACCAGCCACCAGGTTTCAGTGGAAAGTGTTACCTCAGGGAATGCTT  360
HERV-K50B        241 TTTTTTACCATCCCTCTGGCAGAGCAGGATTGTGAAAAATTTGCCTTTACTATACCAGCCATAAATAATAAAGAACCAGCCACCAGGTTTCAGTGGAAAGTGTTACCTCAGGGAATGCTT  360
HERV-K106        241 TTTTTTACCATCCCTCTGGCAGAGCAGGATTGTGAAAAATTTGCCTTTACTATACCAGCCATAAATAATAAAGAACCAGCCACCAGGTTTCAGTGGAAAGTGTTACCTCAGGGAATGCTT  360
HERV-K101        241 TTTTTTACCATCCCTCTGGCAGAGCAGGATTGTGAAAAATTTGCCTTTACTATACCAGCCATAAATAATAAAGAACCAGCCACCAGGTTTCAGTGGAAAGTGTTACCTCAGGGAATGCTT  360
HERV-K103        241 TTTTTTACCATCCCTCTGGCAGAGCAGGATTGCGAAAAATTTGCCTTTACTATACCAGCCATAAATAATAAAGAACCAGCCACCAGGTTTCAGTGGAAAGTGTTACCTCAGGGAATGCTT  360
CERV-K100        241 TTTTTTACCATCCCTCTGGCAGAGCAGGATTGTGAAAAATTTGCCTTTACTATACCAGCCATAAATAATAAAGAACCAGCCACCAGGTTTCAGTGGAAAGTGTTACCTCAGGGAATGCTT  360
HERV-K10         241 TTTTTTACCATCCCTCTGGCAGAGCAGGATTGTGAAAAATTTGCCTTTACTATACCAGCCATAAATAATAAAGAACCAGCCACCAGGTTTCAGTGGAAAGTGTTACCTCAGGGAATGCTT  360
HERV-K108        241 TTTTTTACCATCCCTCTGGCAGAGCAGGATTGCGAAAAATTTGCCTTTACTATACCAGCCATAAATAATAAAGAACCAGCCACCAGGTTTCAGTGGAAAGTGTTACCTCAGGGAATGCTT  360
HERV-K109        241 TTTTTTACCATCCCTCTGGCAGAGCAGGATTGTGAAAAATTTGCCTTTACTATACCAGCCATAAATAATAAAGAACCAGCCACCAGGTTTCAGTGGAAAGTGTTACCTCAGGGAATGCTT  360
HERV-K113        241 TTTTTTACCATCCCTCTGGCAGAGCAGGATTGTGAAAAATTTGCCTTTACTATACCAGCCATAAATAATAAAGAACCAGCCACCAGGTTTCAGTGGAAAGTGTTACCTCAGGGAATGCTT  360
HERV-K36         241 TTTTTTACCATCCCTCTGGCAGAGCAGGATTGTGAAAAATTTGCCTTTACTATACCAGCCATAAATAATAAAGAACCAGCCACCAGGTTTCAGTGGAAAGTGTTACCTCAGGGAATGCTT  360
HERV-K115        241 TTTTTTACCATCCCTCTGGCAGAGCAGGATTGCGAAAAATTTGCCTTTACTATACCAGCCATAAATAATAAAGAACCAGCCACCAGGTTTCAGTGGAAAGTGTTACCTCAGGGAATGCTT  360
HERV-K102        241 TTTTTTACCATCCCTCTGGCAGAGCAGGATTGCGAAAAATTTGCCTTTACTATACCAGCCATAAATAATAAAGAACCAGCCACCAGGTTTCAGTGGAAAGTGTTACCTCAGGGAATGCTT  360
HERV-K50A        241 TTTTTTACCATCCCTCTGGCAGAGCAGGATTGCGAAAAATTTGCCTTTACTATACCAGCCATAAATAATAAAGAACCAGCCACCAGGTTTCAGTGGAAAGTGTTACCTCAGGGAATGCTT  360
                     ********* * ********  * *  ****  *********** ****** *** **** **** ******************* ** ***********  * ****  *********

CERV-KOLD35587   361 AATGGTCCAACTATTTGCCAGACTTTTGTAGCTCAAGCTCTTCAACCAGTTAGAGAC-AGTTTTCAGACTCTTATATCATTCATTATGTTGATGATATTTTGTGTGCTACAGAAAGGAGA  479
HERV-KOLD35587   361 AATGGTCCAACTATTTGCCAGACTTTTGTAGCTCAAGCTCTTCAACCAATTAGAGAC-AGTTTTCAGACTCTTATATCATTCATTATGTTGATGATATTTTGTGTGCTACAGAAAGGAGA  479
SERV-K1 chr.11   361 AATAGTCCAACTATTTGTCATACTTTTGTAGGTCAAGTCCTTCAACCAGTTAGAGACAAATTTTCAGATTGTTCTATCGTTCACTATATTCATGCTATTTTATGTGCTGCAGAAACAAGA  480
Guereza          361 AATAGTCCAACTATTTGTCAAACTTTTGTAGGTCAAGTCCTTCAACCAGTTAGAGACAAATTTTCAGATTGTTATATCATTCACTATATTGATGATATTTTATGTGCTGCAGAAACAAGA  480
SERV-K1 chr.12   361 AATAGTCCAACTATTTGTCAAACTTTCGTAGGTCAAGTCCTTCAACCAGTTAGAGACAAATTTTCAGATTGTTATATCATTCACTATATTGATGATATTTTATGTGCTGCAGAAACAAGA  480
Grivet           361 AATAGTCCAACTATTTGTCAAACTTTCGTGGGTCAAGTCCTTCAACCAGTTAGAGAAAAATTTTCAGATTGTTATATCATTCACTATATTGATGATATTTTATGTGCTGCAGAAACAAGA  480
SERV-K1 chr.5    361 AATAGTCCAACTATTTGTCAAACTTTCGTAGGTCAAGTTCTTCAACCAGTTAGAGACAAATGTTCCGATTGTTACATCATTCACTATATTGATGCTATTTTATGTGCTGCACAAACAAGA  480
CERV-K60         361 AATAGTCCAACTATTTGTCAGATTTTTGTAGGTCGAGCTCTTCAACCAGTTAGAGACAAGTTTTCAGACTGTTATTTTATTCATTATTTTGATGATATTTTATGTGCTGCAGAAACGAAA  480
CERV-K102        361 ACTAGTCCAACTATTTGTCAGACTTTTGTAGGTCGAGCTCTTCAAACAGTTAGAGACAAGTTTTCAGACTGTTATATTATTCATTATATTGATGATATTTTATGTGCTGCAGAAACGAGA  480
HERV-K104        361 AATAGTCCAACTATTTGTCAGACTTTTGTAGGTCGAGCTCTTCAACCAGTTAGAGACAAGTTTTCAGACTGTTATATTATTCATTATATTGATGATATTTTATGTGCTGCAGAAACGAAA  480
HERV-K50D        361 AATAGTCCAACTATTTGTCAGACTTTTGTAGGTCGAGCTCTTCAACCAGTTAGAGACAAGTTTTCAGACTGTTATATTATTCATTATATTGATGATATTTTATGTGCTGCAGAAACGAAA  480
HERV-K50B        361 AATAGTCCAACTATTTGTCAGACTTTTGTAGGTCGAGCTCTTCAACCAGTTAGAGAAAAGTTTTCAGACTGTTATATTATTCATTATATTGATGATATTTTATGTGCTGCAGAAACGAAA  480
HERV-K106        361 AATAGTCCAACTATTTGTCAGACTTTTGTAGGTCGAGCTCTTCAACCAGTTAGAGAAAAGTTTTCAGACTGTTATATTATTCATTATATTGATGATATTTTATGTGCTGCAGAAACGAAA  480
HERV-K101        361 AATAGTCCAACTATTTGTCAGACTTTTGTAGGTCGAGCTCTTCAACCAGTTAGAGAAAAGTTTTCAGACTGTTATATTATTCATTATATTGATGATATTTTATGTGCTGCAGAAACGAAA  480
HERV-K103        361 AATAGTCCAACTATTTGTCAGACTTTTGTAGGTCGAGCTCTTCAACCAGTTAGAGAAAAGTTTTCAGACTGTTATATTATTCATTATATTGATGATATTTTATGTGCTGCAGAAACGAAA  480
CERV-K100        361 AATAGTCCAACTATTTGTCAGACTTTTGTAGGTCGAGCTCTTCAACCAGTGAGAGAAAAGTTTTCAGACTGTTATATTATTCATTATATTGATGATATTTTATGTGCTGCAGAAACGAAA  480
HERV-K10         361 AATAGTCCAACTATTTGTCAGACTTTTGTAGGTCGAGCTCTTCAACCAGTGAGAGAAAAGTTTTCAGACTGTTATATTATTCATTATATTGATGATATTTTATGTGCTGCAGAAACGAAA  480
HERV-K108        361 AATAGTCCAACTATTTGTCAGACTTTTGTAGGTCGAGCTCTTCAACCAGTTAGAGAAAAGTTTTCAGACTGTTATATTATTCATTGTATTGATGATATTTTATGTGCTGCAGAAACGAAA  480
HERV-K109        361 AATAGTCCAACTATTTGTCAGACTTTTGTAGGTCGAGCTCTTCAACCAGTT-----AAAGTTTTCAGACTGTTATATTATTCATTATATTGATGATATTTTATGTGCTGCAGAAACGAAA  475
HERV-K113        361 AATAGTCCAACTATTTGTCAGACTTTTGTAGGTCGAGCTCTTCAACCAGTTAGAGACAAGTTTTCAGACTGTTATATCATTCATTATATTGATGATATTTTATGTGCTGCAGAAACGAAA  480
HERV-K36         361 AATAGTCCAACTATTTGTCAGACTTTTGTAGGTCGAGCTCTTCAACCAGTTAGAGAAAAGTTTTCAGACTGTTATATTATTCATTATATTGATGATATTTTATGTGCTGCAGAAACGAAA  480
HERV-K115        361 AATAGTCCAACTATTTGTCAGACTTTTGTAGGTCGAGCTCTTCAACCAGTTAGAAAAAAGTTTTCAGACTGTTATATTATTCATTATATTGATGATATTTTATGTGCTGCAGAAACGAAA  480
HERV-K102        361 AATAGTCCAACTATTTGTCAGACTTTTGTAGGTTGAGCTCTTCAACCAGTTAGAGAAAAGTTTTCAGACTGTTATATTATTCATTATATTGATGATATTTTATGTGCTGCAGAAACGAGA  480
HERV-K50A        361 AATAGTCCAACTATTTGTCAGACTTTTGTAGGTCGAGCTCTTCAACCAGTTAGAGAAAAGTTTTCAGACTGTTATATTATTCATTATATTGATGATATTTTATGTGCTGCAGAAACGAGA  480
                     * * ************ ** * *** ** * *  **  ****** ** *        * * *** ** * **   *  **** * * ** *** ****** ****** ** ***  * *
```

```
CERV-KOLD35587   480 GACAAATTAATTGACTGTTACACATTTCTGCAGACAGAGGTTGCAAACGCAGGACTGACGACAGCATCTGATAAGATTCAAACCTCTACTCCTTTCCATTACTTGGAAATGCAGGTGGAG  599
HERV-KOLD35587   480 GACAAATTAATTGACTGTTACACATTTCTGCAGACAGAGGTTGCAAACGCAGGACTGACGACAGCATCTGATAAGATTCAAACCTCTACTCCTTTCCATTACTTGGAAATGCAGGTGGAG  599
SERV-K1 chr.11   481 GACAAATTAATTGACTGTTACACATTTCTGCAAGCAGAGGTTGCCAACGCAGGACTAAAAATAGCATCTGATAAGATCCAAACCTCTACTCCTTTTCATTATTTAGGGATGCAGATAGAA  600
Guereza          481 GAAAGATTAATTGACTGTTACACATTTCTGCAAGCAGAGGTTGCCAGCGCAGGACTAACAATAGCATCTGATAAGATCCAAACCTCTGATCCTTTTCATTATTTAGGGATGCAAATAGAA  600
SERV-K1 chr.12   481 GAAAAATTAATTGACTGTTACACATTTCTGCAAGCAGAGGTTGCCAACGCAGGACTAACAATAGCATCGGATAAGATCCAAACCTCTGCTCCTTTTCATTATTTAGGGATGCAGATAGAA  600
Grivet           481 GAAAAATTAATTGACTGTTATACATTTCTGCAAGCAGAGGTTGCCAACGCAGGACTAACAATAGCATCTGATAAGATCCAAACCTCTGCTCCTTTTCATTATTTAGGGATGCAGATAGAA  600
SERV-K1 chr.5    481 GACAAATGAATTGACTGTTACACATTTCTGCAAGCAGAGGTTGCCAACGCAGGACTGACAAGAGCATCTGATAAGATTCAGACCTCTACTCCTTTTCATTATTTAGGGATGCAGATAGAA  600
CERV-K60         481 GATAAATTAATTGACTGTTATACATTTCTGCAAGCAGAGGTTGCCAATGCAGGACTGGCAATAGCATCTGATAAGATCCAAACCTCTACTCCTTTTCATTATTTAGGGATGCAGATAGAA  600
CERV-K102        481 GATAAATTAATTGACTGTTACACATTTCTGCAAGCAGAGGTTGCCAACGCAGGACTGGCAATAGCATCTGATAAGATCCAAACCTCTACTCCTTTTCATTATTTAGGGATGCAGATAGAA  600
HERV-K104        481 GATAAATTAATTGACTGTTATACATTTCTGCAAGCAGAGGTTGCCAACGCAGGACTGGCAATAGCATCTGATAAGATCCAAACCTCTACTCCTTTTCATTATTTAGGGATGCAGATAGAA  600
HERV-K50D        481 GATAAATTAATTGACTGTTATACATTTCTGCAAGCAGAGGTTGCCAACGCAGGACTGGCAATAGCATCTGATAAGATCCAAACCTCTACTCCTTTTCATTATTTAGGGATGCAGATAGAA  600
HERV-K50B        481 GATAAATTAATTGACTGTTATACATTTCTGCAAGCAGAGGTTGCCAATGCTGGACTGGCAATAGCATCTGATAAGATCCAAACCTCTACTCCTTTTCATTATTTAGGGATGCAGATAGAA  600
HERV-K106        481 GATAAATTAATTGACTGTTATACATTTCTGCAAGCAGAGGTTGCCAATGCTGGACTGGCAATAGCATCTGATAAGATCCAAACCTCTACTCCTTTTCATTATTTAGGGATGCAGATAGAA  600
HERV-K101        481 GATAAATTAATTGACTGTTATACATTTCTGCAAGCAGAGGTTGCCAGTGCTGGACTGGCAATAGCATCTGATAAGATCCAAACCTCTACTCCTTTTCATTATTTAGGGATGCAGATAGAA  600
HERV-K103        481 GATAAATTAATTGACTGTTATACATTTCTGCAAGCAGAGGTTGCCAATGCTGGACTGGCAATAGCATCTGATAAGATCCAAACCTCTACTCCTTTTCATTATTTAGGGATGCAGATAGAA  600
CERV-K100        481 GATAAATTAATTGACTGTTATACATTTCTGCAAGCAGAGGTTGCCAATGCTGGACTGGCAATAGCATCCGATAAGATCCAAACCTCTACTCCTTTTCATTATTTAGGGATGCAGATAGAA  600
HERV-K10         481 GATAAATTAATTGACTGTTATACATTTCTGCAAGCAGAGGTTGCCAATGCTGGACTGGCAATAGCATCCGATAAGATCCAAACCTCTACTCCTTTTCATTATTTAGGGATGCAGATAGAA  600
HERV-K108        481 GATAAATTAATTGACTGTTATACATTTCTGCAAGCAGAGGTTGCCAATGCTGGACTGGCAATAGCATCTGATAAGATCCAAACCTCTACTCCTTTTCATTATTTAGGGATGCAGATAGAA  600
HERV-K109        476 GATAAATTAATTGACTGTTATACATTTCTGCAAGCAGAGGTTGCCAATGCTGGACTGGCAATAGCATCTGATAAGATCCAAACCTCTACTCCTTTTCATTATTTAGGGATGCAGATAGAA  595
HERV-K113        481 GATAAATTAATTGACTGTTATACATTTCTGCAAGCAGAGGTTGCCAATGCTGGACTGGCAATAGCATCTGATAAGATCCAAACCTCTACTCCTTTTCATTATTTAGGGATGCAGATAGAA  600
HERV-K36         481 GATAAATTAATTGACTGTTATACATTTCTGCAGGCAGAGGTTGCCAATGCTGGACTGGCAATAGCATCTGATAAGATCCAAACCTCTACTCCTTTTCATTATTTAGGGATGCAGATAGAA  600
HERV-K115        481 GATAAATTAATTGACTGTTATACATTTCTGCAAGCAGAGGTTGCCAGTGCTGGACTGGCAATAGCATCTGATAAGATCCAAACCTCTACTCCTTTTCATTATTTAGGGATGCAGATAGAA  600
HERV-K102        481 GATAAATTAATTGACTGTTATACATTTCTGCAAGCAGAGGTTGCCAATGCTGGACTGGCAATAGCATCTGATAAGATCCAAACCTCTACTCCTTTTCATTATTTAGGGATGCAGATAGAA  600
HERV-K50A        481 GATAAATTAATTGACTGTTATACATTTCTGCAAGCAGAGGTTGCCAATGCTGGACTGGCAATAGCATCTGATAAGATCCAAACCTCTACTCCTTTTCATTATTTAGGGATGCAGATAGAA  600
                     ** * ** ************ ***********  ********** *  ** *****     * ****** ******** ** ******  ****** ***** ** *  *****  * **

CERV-KOLD35587   600 GAAATGAAAATTAAACCACAAAAAATAGAAATAAGAAAAGACACATTAAAAACATTAAATGACTTTCAAAAATTGCTAGGAGATATTAATTGGATTCAGCCAACTCTAGGCATCTCTACT  719
HERV-KOLD35587   600 GAAACGAAAATTAAACCATAAAAAATAGAAATAAGAAAAGACACATTAAAAACATTAAATGACTTTCAAAAATTGCTAGGAGATATTAATTGGATTCAGCCAACTCTAGGCATCTCTACT  719
SERV-K1 chr.11   601 AATAGAAAAATTAAGCCACAAAAAATAGAAATAAGAAAAGACACATTAAAAACATTAAATGACTTTCAGAAATTGTTAGGCGATATTAATTGGATTCGGCCAACTCTAGGCATTCCTACT  720
Guereza          601 AATAGAAAAATTAAGCCACAAAAAATAGAAATAAGAAAAGATGCATTAAAAACATTAAATGATTTTCAAAAATTGTTAGGTGATATTAATTGGATTCGGCCAACTCTAGGCATTCCTACT  720
SERV-K1 chr.12   601 AGTAGAAAAATTAAGCCACAAAAAATAGAAATAAGAAAAGATACTTTAAAAACTTTAAATGACTTTCAAAAATTGTTAGGCGATATTAATTGGATTCGGCCAACTCTAGGCATTCCTACT  720
Grivet           601 AATAGAAAAATTAAGCCACAAAAAATAGAAATAAGAAAAGATACATTAAAAACATTAAATGACTTTCAAAAATTGTTAGGCGATATTAATTGGATTCGGCCAACTCTAGGCATTCCTACT  720
SERV-K1 chr.5    601 ATTAGAAAATTTAAGCCACAAAAAATAGAAATAA-----GACACATTAAAAACACCAAATGACTTTCAGAAATTGCTAGGCGATATTAATTGGATTCGGCCAACTCTAGGCATTCCTACT  715
CERV-K60         601 AATAGAAAAATTAAGCCACAAAAAATAGAAATAAGAAAAGACACATTAAAAACACTAAATGATTTTCAAAAATTGCTGGGAGATATTAATTGGATTCGGCCAACTCTAGGCATTCCTACT  720
CERV-K102        601 AATAGAAAAATTAAGCCACAAAAAATAGAAATAAGAAAAGACACATTAAAAACACTAAATGATTTTCAAAAATTGCTGGGAGATATTAATTGGATTCGGCCAACTCTAGGCATTCCTACT  720
HERV-K104        601 AATAGAAAAATTAAGCCACAAAAAATAGAAATAAGAAAATACACATTAAAAACACTAAATGATTTTCAAAAATTGCTGGGAGATATTAATTGGATTCGGCCAACTCTAGGCATTCCTACT  720
HERV-K50D        601 AATAGAAAAATTAAGCCACAAAAAATAGAAATAAGAAAATACACATTAAAAACACTAAATGATTTTCAAAAATTGCTGGGAGATATTAATTGGATTCGGCCAACTCTAGGCATTCCTACT  720
HERV-K50B        601 AATAGAAAAATTAAGCCACAAAAAATAGAAATAAGAAAAGACACATTAAAAACACTAAATGATTTTCAAAAATTACTAGGAGATATTAATTGGATTCGGCCAACTCTAGGCATTCCTACT  720
HERV-K106        601 AATAGAAAAATTAAGCCACAAAAAATAGAAATAAGAAAAGACACATTAAAAACACTAAATGATTTTCAAAAATTACTAGGAGATATTAATTGGATTTGGCCAACTCTAGGCATTCCTACT  720
HERV-K101        601 AATAGAAAAATTAAGCCACAAAAA-TAGAAATAAGAAAAGACACATTAAAAACACTAAATGATTTTCAAAAATTACTAGGAGATATTAATTGGATTCGGCCAACTCTAGGCATTCCTACT  719
HERV-K103        601 AATAGAAAAATTAAGCCACAAAAAATAGAAATAAGAAAAGACACATTAAAAACACTAAATGATTTTCAAAAATTACTAGGAGATATTAATTGGATTCGGCCAACTCTAGGCATTCCTACT  720
CERV-K100        601 AATAGAAAAATTAAGCCACAAAAAATAGAAATAAGAAAAGACACATTAAAAACACTAAATGATTTTCAAAAATTACTAGGAGATATTAATTGGATTCGGCCAACTCTAGGCATTCCTACT  720
HERV-K10         601 AATAGAAAAATTAAGCCACAAAAAATAGAAATAAGAAAAGACACATTAAAAACACTAAATGATTTTCAAAAATTACTAGGAGATATTAATTGGATTCGGCCAACTCTAGGCATTCCTACT  720
HERV-K108        601 AATAGAAAAATTAAGCCACAAAAAATAGAAATAAGAAAAGACACATTAAAAACACTAAATGATTTTCAAAAATTACTAGGAGATATTAATTGGATTCGGCCAACTCTAGGCATTCCTACT  720
HERV-K109        596 AATAGAAAAATTAAGCCACAAAAAATAGAAATAAGAAAAGACACATTAAAAACACTAAATGATTTTCAAAAATTACTAGGAGATATTAATTGGATTTGGCCAACTCTAGGCATTCCTACT  715
HERV-K113        601 AATAGAAAAATTAAGCCACAAAAAATAGAAATAAGAAAAGACACATTAAAAACACTAAATGATTTTCAAAAATTACTAGGAGATATTAATTGGATTCGGCCAACTCTAGGCATTCCTACT  720
HERV-K36         601 AATAGAAAAATTAAGCCACAAAAAATAGAAATAAGAAAAGACACATTAAAAGCACTAAATGATTTTCAAAAATTACTAGGAGATATTAATTGGATTCGGCCAACTCTAGGCATTCCTACT  720
HERV-K115        601 AATAGAAAAATTAAGCCACAAAAAATAGAAATAAGAAAAGACACATTAAAAACACTAAATGATTTTCAAAAATTACTAGGAGATATTAATTGGATTCAGCCAACTCTAGGCATTCCTACT  720
HERV-K102        601 AATAGAAAAATTAAGCCACAAAAAGTAGAAATAAGAAAAGACACATTAAAAACACTAAATGATTTTCAAAAATTACTAGGAGATATTAATTGGATTCGGCCAACTCTAGGCATTCCTACT  720
HERV-K50A        601 AATAGAAAAATTAAGCAACAAAAAATAGAAATAAGAAAAGACACATTAAAAACACTAAATGATTTTCAAAAATTACTAGGAGATATTAATTGGATTCGGCCAACTCTAGGCATTCCTACT  720
                        *  *** **** * * ***** *********       *  * ****** *    ****** ***** *****  * ** **************  **************  *****

CERV-KOLD35587   720 TATGCCATGTCAAATTTGTTCTCTATCTTAAGAGGGGATCCAGAATTGAATAGTAAAAGAACATTAACTCCAGAAGCAACTAAAGAAATTGAATTAGTTGAAGAAAAAATTCGGTCAGCA  839
HERV-KOLD35587   720 TATGCCATGTCAAATTTGTTCTCTATCTTAAGAGGGGATCCAGAATTGAATAGTAAAAGAACATTAACTCCAGAAGCAACTAAAGAAATTGAATTAGTTGAAGAAAAAATTCGGTCAGCA  839
SERV-K1 chr.11   721 TATGCCATGTCAAATTTGTTCTCTATCCTTAGAGGAGACCCAGACTTAAATAGTAAAAGAATATTAACCCCAGAGGCAACAAAAGAAATTAAATTAGTGGAAGAAAAAATTCAGTCAGCG  840
Guereza          721 TATGCCATGTCAAATTTGTTCTCTATCCTAAGAGGAGATCCAGACTTAAATAGTAGGAGAATATTAACCCCAGAGGCAACAAAAGAAATTAAATTAGTGGAAGAAAAAATTCAGTCAGCG  840
SERV-K1 chr.12   721 TATGCCATGTCAAATTTGTTCTCTATCCTAAGAGGAGATCCAGACTTAAATAGTAAAAGAATATTAACCCCAGAGGCAACAAAAGAAATTAAATTAGTGGAAGAAAAAATTCAGTCAGCG  840
Grivet           721 TATGCCATGTCAAATTTGTTCTCTATCCTAAGAGGAGATCCAGACTTAAATAGTAAAAGAATATTAACCCCAGAGGCAACAAAAGAAATTAAATTAGTGGAAGAAAAAATTCAGTCAGCG  840
SERV-K1 chr.5    716 TATGCCATGTCAAATTTGTTCTCTATCCTAAGAGGAGACCCAGACTTAAACAGTAAAAGAATATTAACCCCAGAGGCAACAAAAGAAATTAAATTAGTGGAAGAAAAAATTCAGTCAGTG  835
CERV-K60         721 TATGCCATGTCAAATTTGTTCTCTATCTCAAGAGGAGACTCAGACTTAAATAGTAAAAGAATGTTAACCCCAGAGGCAACAAAAGAAATTAAATTAGTGGAAGAAAAAATTCAGTCAGCG  840
CERV-K102        721 TATGCCACGTCAAATTTGTTCTCTATCTTAAGAGGAGACTCAGACTTAAATAGTAAAAGAATGTTAACCCCAGAGGCAACAAAAGAAATTAAATTAGTGGAAGAAAAAATTCAGTCAGCA  840
HERV-K104        721 TATGCCATGTCAAATTTGTTCTCTATCTTAAGAGGAGACTCAGACTTAAATAGTAAAAGAATGTTAACCCCAGAGGCAACAAAAGAAATTAAATTAGTGGAAGAAAAAATTCAGTCAGTG  840
HERV-K50D        721 TATGCCATGTCAAATTTGTTCTCTATCTTAAGAGGAGACTCAGACTTAAATAGTAAAAGAATGTTAACCCCAGAGGCAACAAAAGAAATTAAATTAGTGGAAGAAAAAATTCAGTCAGTG  840
HERV-K50B        721 TATGCCATGTCAAATTTGTTCTCTATATTAAGAGGAGACTCAGACTTAAATAGTAAAAGAATATTAACCCCAGAGGCAACAAAAGAAATTAAATTAGTGGAAGAAAAAATTCAGTCAGCG  840
HERV-K106        721 TATGCCATGTCAAATTTGTTCTCTATCTTAAGAGGAGACTCAGACTTAAATAGTAAAAGAATGTTAACCCCAGAGGCAACAAAAGAAATTAAATTAGTGGAAGAAAAAATTCAGTCAGCG  840
HERV-K101        720 TATGCCATGTCAAATTTGTTCTCTATCTTAAGAGGAGACTCAGACTTGAATAGTAAAAGAATATTAACCCCAGAGGCAACAAAAGAAATTAAATTAGTGGAAGAAAAAATTCAGTCAGCG  839
HERV-K103        721 TATGCCATGTCAAATTTGTTCTCTATCTTAAGAGGAGACTCAGACTTAAATAGTAAAAGAATATTAACCCCAGAGGCAACAAAAGAAATTAAATTAGTGGAAGAAAAAATTCAGTCAGCG  840
CERV-K100        721 TATGCCATGTCAAATTTGTTCTCTATCTTAAGAGGAGACTCAGACTTAAATAGTCAAAGAATATTAACCCCAGAGGCAACAAAAGAAATTAAATTAGTGGAAGAAAAAATTCAGTCAGCG  840
HERV-K10         721 TATGCCATGTCAAATTTGTTCTCTATCTTAAGAGGAGACTCAGACTTAAATAGTCAAAGAATATTAACCCCAGAGGCAACAAAAGAAATTAAATTAGTGGAAGAAAAAATTCAGTCAGCG  840
HERV-K108        721 TATGCCATGTCAAATTTGTTCTCTATCTTAAGAGGAGACTCAGACTTAAATAGTAAAAGAATGTTAACCCCAGAGGCAACAAAAGAAATTAAATTAGTGGAAGAAAAAATTCAGTCAGCG  840
HERV-K109        716 TATGCCATGTCAAATTTGTTCTCTATCTTAAGAGGAGACTCAGACTTAAATAGTAAAAGAATGTTAACCCCAGAGGCAACAAAAGAAATTAAATTAGTGGAAGAAAAAATTCAGTCAGCG  835
HERV-K113        721 TATGTCATGTCAAATTTGTTCTCTATCTTAAGAGGAGACTCAGACTTAAATAGTAAAAGAATGTTAACCCCAGAGACAACAAAAGAAATTAAATTAGTGGAAGAAAAAATTCAGTCAGCA  840
HERV-K36         721 TATGCCATGTCAAATTTGTTCTCTATCTTAAGAGGAGACTCAGACTTAAATAGTAAAAGAATGTTAACCCCAGAGGCAACAAAAGAAATTAAATTAGTGGAAGAAAAAATTCAGTCAGCG  840
HERV-K115        721 TATGCCATGTCAAATTTGTTCTCTATCTTAAGAGGAGACTCAGACTTAAATAGTAAAAGAATATTAACCCCAGAGGCAACAAAAGAAATTAAATTAGTGGAAGAAAAAATTCAGTCAGCA  840
HERV-K102        721 TATGCCATGTCAAATTTGTTCTCTATCTTAAGAGGAGACTCAGACTTAAATAGTAAAAGAATATTAACCCCAGAGGCAACAAAAGAAATTAAATTAGTGGAAGAAAAAATTCAGTCAGCG  840
HERV-K50A        721 TATGCCATGTCAAATTTGTTCTCTATCTTAAGAGGAGACTCAGACTTAAATAGTAAAAGAATATTAACCCCAGAGGCAACAAAAGAAATTAAATTAGTGGAAGAAAAAATTCAGTCAGCG  840
                     **** ** *****************    ***** **  **** ** ** ***    ****  ***** *****  **** ********* ******* ************ *****

CERV-KOLD35587   840 CAAGTAAATAGAATAGATCACTTAGCCCCACTCCAACTTTTGATTTTTGCTACTGCACATTCTCCAACAGGCATTATTGTTCAAAATACAGATCTTGTGGAGTGGTTCTTCCTTCCTCAT  959
HERV-KOLD35587   840 CAAGTAAATAGAATAGATCACTTAGCCCCACTCCAACTTTTGATTTTTGCTACTGCACATTCTCCAACAGGCATTATTGCTCAAAATACAGATCTTGTGGAGTGGTTCTTCCTTCCTCAT  959
SERV-K1 chr.11   841 CAAATAAGTAAAATAGATCCCTTAGCCCCACTCCAACTTTTGATTTTTGCTACTGCACATTCTCCAACAGGCATCATTGTTCAAAATACTGATCTTGTGGAGTGGTCATTCCTTCCTCAC  960
Guereza          841 CAAATAAATAGAATAGATTCTTTAGCCCCACTCCAACTTTTAATTTTTGCCACTGCACATTCTCCAACAGGCATCATTGTTCAAAATACTGATCTTGTAGAGTGGTCATTCCTTCCTCAC  960
SERV-K1 chr.12   841 CAAATAAATAGGATAGATCCTTTAGCCCCACTCCAACTTTTGATTTTTGCTACTACACATTCTCCAACAGGCATCATTATTCAAAACACTGATCTTGTGGAGTGGTCATTTCTTTCTCAC  960
Grivet           841 CAAATAAGTAGAATAGATCCTTTAGCCCCACTCCAACTTTTAATTTTTGCTACTACACATTCTCCAACAGGCATCATTATTCAAAATACTGATCTTGTGGAGTGGTCATTTCTTCCTCAC  960
SERV-K1 chr.5    836 CAAATAAGTAGAATAGATCCCTTAGCCCCACTCCAACTTTTGATTTTTGCTACTGCACATTCTCCAACAGGCAGCATTGTTCAAAATACTGATCTTGTGGAGTGGTCGTTCCTTCCTCAG  955
CERV-K60         841 CAAATAAATAGAATAGATCCCTTAGCCCCACTCCAACTTTTGATTTTTGCCACTGCACATTCTCCAACAGGCATCATTATTCAAAATACTGATCTTGTGGAGTGGTCATTCCTTCCTCGC  960
CERV-K102        841 CAAATAAATAGAATAGATCCCTTAGCCCCACTCCAACTTTTGATTTTTGCCACTGCACATTCTCCAACAGGCATCATTATTCAAAATACTGATCTTGTGGAGTGGTCATTCCCTCCTCAC  960
HERV-K104        841 CAAATAAATAGAATAGATCCCTTAGCCCCACTCCAACTTTTGATTTTTGCCACTGCACATTCTCCAACAGGCATCATTATTCAAAATACTGATCTTGTGGAGTGGTCATTCCTTCCTCAC  960
HERV-K50D        841 CAAATAAATAGAATAGATCCCTTAGCCCCACTCCAACTTTTGATTTTTGCCACTGCACATTCTCCAACAGGCATCATTATTCAAAATACTGATCTTGTGGAGTGGTCATTCCTTCCTCAC  960
HERV-K50B        841 CAAATAAATAGAATAGATCCCTTAGCCCCACTCCAACTTTTGATTTTTGCCACTGCACATTCTCCAACAGGCATCATTATTCAAAATACTGATCTTGTGGAGTGGTCATTCCTTCCTCAC  960
HERV-K106        841 CAAATAAATAGAATAGATCCCTTAGCCCCACTCCAACTTTTGATTTTTGCCACTGCACATTCTCCAACAGGCATCATTATTCAAAATACTGATCTTGTGGAGTGGTCATTCCTTCCTCAC  960
HERV-K101        840 CAAATAAATAGAATAGATCCCTTAACCCCACTCCAACTTTTGATTTTTGCCACTGCACATTCTCCAACAGGCATCATTATTCAAAATACTGATCTTGTGGAGTGGTCATTCCTTCCTCAC  959
HERV-K103        841 CAAATAAATAGAATAGGTCCCTTAGCCCCACTCCAACTTTTGATTTTTGCCACTGCACATTCTCCAACAGGCATCATTATTCAAAATACTGATCTTGTGGAGTGGTCATTCCTTCCTCAC  960
CERV-K100        841 CAAATAAATAGAATAGATCCCTTAGCCCCACTCCAACTTTTGATTTTTGCCACTGCACATTCTCCAACAGGCATCATTATTCAAAATACTGATCTTGTGGAGTGGTCATTCCTTCCTCAC  960
HERV-K10         841 CAAATAAATAGAATAGATCCCTTAGCCCCACTCCAACTTTTGATTTTTGCCACTGCACATTCTCCAACAGGCATCATTATTCAAAATACTGATCTTGTGGAGTGGTCATTCCTTCCTCAC  960
HERV-K108        841 CAAATAAATAGAATAGATCCCTTAGCCCCACTCCAACTTTTGATTTTTGCCACTGCACATTCTCCAACAGGCATCATTATTCAAAATACTGATCTTGTGGAGTGGTCATTCCTTCCTCAC  960
HERV-K109        836 CAAATAAATAGAATAGATCCCTTAGCCCCACTCCAACTTTTGATTTTTGCCACTGCACATTCTCCAACAGGCATCATTATTCAAAATACTGATCTTGTGGAGTGGTCATTCCTTCCTCAC  955
HERV-K113        841 CAAATAAATAGAATAGATCCCTTAGCCCCACTCCGACTTTTGATTTTTGCCACTGCACATTCTCCAATAGGCATCATTATTCAAAATACTGATCTTGTGGAGTGGTCATTCCTTCCTCAC  960
HERV-K36         841 CAAATAAATAGAATAGATCCCTTAGCCCCACTCCAACTTTTGATTTTTGCCACTGCACATTCTCCAACAGGCATCATTATTCAAAATACTGATCTTGTGGAGTGGTCATTCCTTCCTCAC  960
HERV-K115        841 CAAATAAATAGAATAGATCCCTTAGCCCCACTCCAACTTTTGATTTTTGCCACTGCACATTCTCCAACAGGTATCATTATTCAAAATACTGATCTTGTGGAGTGGTCATTCCTTCCTCAC  960
HERV-K102        841 CAAATAAATAGAATAGATCCCTTAGCCCCACTCCAACTTTTGATTTTTGCCACTGCACATTCTCCAACAGGTATCATTATTCAAAATACTGATCTTGTGGAGTGGTCATTCCTTCCTCAC  960
HERV-K50A        841 CAAATAAATAGAATAGATCCCTTAGCCCCACTCCAACTTTTGATTTTTGCCACTGCACATTCTCCAACAGGTATCATTATTCAAAATACTGATCTTGTGGAGTGGTCATTCCTTCCTCAC  960
                     *** *** **  **** *   *** ********* ****** ******** *** ************ *** *  ***  ****** ** ******** *******  ** * * ***
```

```
CERV-KOLD35587   960 AGTACAATTAAGACTTTTACATTGTACTTGGATCAAATGGCTACATTAATTGGTCAGGCAAGATTACGAATTATAAAATTGTGTGAAAGTGACCCAGATAAAATCATTGTTCCTTTAAAC 1079
HERV-KOLD35587   960 AGTACAATTAAGACTTTTACATTGTACTTGGATCAAATGGCTACATTAATTGGTCAGGCAAGATTACGAATAATAAAATTGTGTGAAAGTGACTCAGATAAAATCATTGTTCCTTTAAAC 1079
SERV-K1 chr.11   961 AGTACAATTAAGACTTTTACATTGTACTTGGATGAAATAGCTACATTAATTGGTCAGGCAAGATTACAAATAATAAAATTGTGTGGTAATGACCCAGACAAAATAGTTGTTCCCTTAACC 1080
Guereza          961 AGTACAATTAAAACTTTTACATTGTACTTGGATCAAATAGCTACATTAATAGGTCAGACAAGATTACGAATAATAAAATTGTGTGGTAATGACCCAGACAAAATAGTTGTTCCTTTAACC 1080
SERV-K1 chr.12   961 AGTACAATTAAGACTTTTACATTGTACTTGGATCGAATAGCTACATTAATTGGTCAGGCAAGATTACGGATAATAAAATTGTGTGGTAATGACCCAGACAAAATAGTTGTTCCTTTAACC 1080
Grivet           961 AGTACAATTAAGACTTTTACATTGTACTTGGATCAAATAGCTACATTAATTGGTCAGGCAAGATTACGAATAATAAAATTATGTGGTAATGACCCAGACAAAATAGTTGTTCCCTTAACC 1080
SERV-K1 chr.5    956 AGTACAATTAAGACTTTTACGTTGTACTTGGATCAAATAGCTACATTAATTGGTCAGGCAAGATTACGAATAATAAAATTGTGTGGAAATGACCCAGACAAAATAGTTGTTCCTTTAACC 1075
CERV-K60         961 AGTACAGTTAAGACTTTTACATTGTACTTGGATCAAATAGCTACATTAATTGGTCAGACAAGATTACGAATAATAAAATTATGTGGAAATGACCCAGACAAAATAGTTGTCCCTTTAACC 1080
CERV-K102        961 AGTACAGTTAAGACTTTTACATTGTACTTGGATCAAATAGCTACATTAATTGGTCAGACAAGATTACGAATAATAAAATTATGTGGCAATGACCCAGACAAAATAGTTGTCCCTTTAACC 1080
HERV-K104        961 AGTACAGTTAAGACTTTTACATTGTACTTGGATCAAATAGCTACATTAATTGGTCAGACAAGATTACGAATAATAAAATTATGTGGAAATGACCCAGACAAAATAGTTGTCCCTTTAACC 1080
HERV-K50D        961 AGTACAGTTAAGACTTTTACATTGTACTTGGATCAAATAGCTACATTAATTGGTCAGACAAGATTACGAATAATAAAATTATGTGGAAATGACCCAGACAAAATAGTTGTCCCTTTAACC 1080
HERV-K50B        961 AGTACAGTTAAGACTTTTACATTGTACTTGGATCAAATAGCTACATTAATCGGTCAGACAAGATTACGAATAATAAAATTATGTGGAAATGACCCAGACAAAATAGTTGTCCCTTTAACC 1080
HERV-K106        961 AGTACAGTTAAGACTTTTACATTGTACTTGGATCAAATAGCTACATTAATCGGTCAGACAAGATTACGAATAATAAAATTATGTGGAAATGACCCAGACAAAATAGTTGTCCCTTTAACC 1080
HERV-K101        960 AGTACAGTTAAGACTTTTACATTGTACTTGGATCAAATGGCTACATTAATCGGTCAGACAAGATTACGAATAATAAAATTATGTGGAAATGACCAAGACAAAATAGTTGTCCCTTTAACC 1079
HERV-K103        961 AGTACAGTTAAGACTTTTACATTGTACTTGGATCAAATAGCTACATTAATCGGTCAGACAAGATTACGAATAACAAAATTATGTGGAAATGACCCAGACAAAATAGTTGTCCCTTTAACC 1080
CERV-K100        961 AGTACAGTTAAGACTTTTACATTGTACTTGGATCAAATAGCTACATTAATCGGTCAGACAAGATTACGAATAACAAAATTATGTGGAAATGACCCAGACAAAATAGTTGTCCCTTTAACC 1080
HERV-K10         961 AGTACAGTTAAGACTTTTACATTGTACTTGGATCAAATAGCTACATTAATCGGTCAGACAAGATTACGAATAACAAAATTATGTGGAAATGACCCAGACAAAATAGTTGTCCCTTTAACC 1080
HERV-K108        961 AGTACAGTTAAGACTTTTACATTGTACTTGGATCAAATAGCTACATTAATCGGTCAGACAAGATTACGAATAATAAAATTATGTGGGAATGACCCAGACAAAATAGTTGTCCCTTTAACC 1080
HERV-K109        956 AGTACAGTTAAGACTTTTACACTGTACTTGGATCAAATAGCTACATTAATCGGTCAGACAAGATTACGAATAATAAAATTATGTGGAAATGACCCAGACAAAATAGTTGTCCCTTTAACC 1075
HERV-K113        961 AGTACAGTTAAGACTTTTACATTGTACTTGGATCAAATAGCTACATTAATCGGTCAGACAAGATTACGAATAATAAAATTATGTGGGAATGACCCAGACAAAATAGTTGTCCCTTTAACC 1080
HERV-K36         961 AGTACAGTTAAGACTTTTACACTGTACTTGGATCAAATAGCTACATTAATTGGTCAGACAAGATTACGAATAATAAAATTATGTGGAAATGACCCAGACAAAATAGTTGTCCCTTTAACC 1080
HERV-K115        961 AGTACAGTTAAGACTTTTACACTGTACTTGGATCAAATAGCTACATTAATCGGTCAGACAAGATTACGAATAATAAAATTATGTGGAAATGACCCAGACAAAATAGTTGTCCCTTTAACC 1080
HERV-K102        961 AGTACAGTTAAGACTTTTACACTGTACTTGGATCAAATAGCTACATTAATTGGTCAGACAAGATTACGAATAATAAAATTATATGGAAATGACCCAGACAAAATAGTTGTCCCTTTAACC 1080
HERV-K50A        961 AGTACAGTTAAGACTTTTACACTGTACTTGGATCAAATAGCTACATTAATTGGTCAGACAAGATTACGAATAATAAAATTATGTGGAAATGACCCAGACAAAATAGTTGTCCCTTTAACC 1080
                     ****** **** ********  ***********  *** *********** ****** *********   ** * ****** * **   * ****   *** *****   **** ** **** *

CERV-KOLD35587  1080 AAGGAACAGGTTAGACAAGCCTTTATCAATTCTGGTGCATGGCAGATTGGTCTTGCTGATTTTGTGGGAATTATTGACAATCATTACCCAAAAACAAAAATCTTCCAGTTTTTAAAATTG 1199
HERV-KOLD35587  1080 AAGGAACAGGTTAGACAAGCCTTTATCAATTCTGGTGCATGGCAGATTGGTCTTGCTGATTTTGTGGGAATTATTGACAATCATTACCCAAAAACAAAAATCTTCCAGTTTTTAAAATTG 1199
SERV-K1 chr.11  1081 AAGGAACAAGTTAGACAAGCCTTTATCAATTCTGGTGCATAGCAGATTGGTCTTGCTGATTTTGTGGGAATTATTGATAATCATTACCCAAAAACAAAAATCTTCCAGTTTTTAAAATTG 1200
Guereza         1081 AAGGAACAAGTTAAACAAGCCTTTATCAATTCTGGTGCATGGCAAATTGGCCTTGCTGATTTTGTGGGAATTATTGATAATCATTACCCAAAAACAAAAATCTTCCAGTTTTTAAAATTG 1200
SERV-K1 chr.12  1081 AAGGAACAAGTTAGGCAAGCCTTTATCAATTCTGGTGCATGGCAGATTGGCCTTGCTAATTTTGTGGGAGTTATTGATAATCATTACCCAAAAACAAAAATCTTCCAGTTTTTAAAATTG 1200
Grivet          1081 AAGGAACAAGTTAGGCAAGCCTTTATCAATTCTGCTGCATGGCAGATTGGCCTTGCTAATTTTGTGGGAGTTATTGATAATCACTACCCAAAAACAAAAATCTTCCAGTTTTTAAAATTG 1200
SERV-K1 chr.5   1076 AAGGAACAAGTTAGACAAGCCTTTATCAGTTCTGGTGCATGGCAGATTGGTCTTGTTGATTTTGTGGGAATTATTGATAATCATTACCCAAAACCAAAAATCTTCCAGTTTTTAAAATTG 1195
CERV-K60        1081 AAGGAACAAGTTAGACAAGCCTTTATCAATTCTGGTGCATGGCAGATTGGTCTTGCTAATTTTGTGGGAATTATTGATAATCATTACCCAAAAACAAAAATCTTCCAGTTCTTAAAATTG 1200
CERV-K102       1081 AAGGAACAAGTTAGACAAGGCTTTATCAATTCTGGTGCATGGCAGATTGGTCTTGCTAATTTTGTGGGAATTATTGATAATCATTACCCAAAAACAAAAATCTTCCAGTTCTTAAAATTG 1200
HERV-K104       1081 AAGGAACAAGTTAGACAAGACTTTATCAATTCTGGTGCATGGCAGATTGGTCTTGCTAATTTTGTGGGAATTATTGATAATCATTACCCAAAAACAAAGATCTTCCAGTTCTTAAAATTG 1200
HERV-K50D       1081 AAGGAACAAGTTAGACAAGCCTTTATCAATTCTGGTGCATGGCAGATTGGTCTTGCTAATTTTGTGGGAATTATTGATAATCATTACCCAAAAACAAAGATCTTCCAGTTCTTAAAATTG 1200
HERV-K50B       1081 AAGGAACAAGTTAGACAAGCCTTTATCAATTCTGGTGCATGGCAGATTGGTCTTGCTAATTTTGTGGGAATTATTGATAATCATTACCCAAAAACAAAGATCTTCCAGTTCTTAAAAATG 1200
HERV-K106       1081 AAGGAACAAGTTAGACAAGCCTTTATCAATTCTGGTGCATGGCAGATTGGTCTTGCTAATTTTGTGGGAATTATTGATAATCATTACCCAAAAACAAAGATCTTCCAGTTCTTAAAACTG 1200
HERV-K101       1080 AAGGAACAAGTTAGACAAGCCTTTATCAATTCTGGTGCATGGCAGATTGGTCTTGCTAATTTTGTGGGAATTATTGATAATCATTACCCAAAAACAAAGATCTTCCAGTTCTTAAAATTG 1199
HERV-K103       1081 AAGGAACAAGTTAGACAAGCCTTTATCAATTCTGGTGCATGGCAGATTGGTCTTGCTAATTTTGTGGGAATTATTGATAATCATTACCCAAAAACAAAGATCTTCCAGTTCTTAAAATTG 1200
CERV-K100       1081 AAGGAACAAGTTAGACAAGCCTTTATCAATTCTGGTGCATGGCAGATTGGTCTTGCTAATTTTGTGGGACTTATTGATAATCATTACCCAAAAACAAAGATCTTCCAGTTCTTAAAATTG 1200
HERV-K10        1081 AAGGAACAAGTTAGACAAGCCTTTATCAATTCTGGTGCATGGCAGATTGGTCTTGCTAATTTTGTGGGACTTATTGATAATCATTACCCAAAAACAAAGATCTTCCAGTTCTTAAAATTG 1200
HERV-K108       1081 AAGGAACAAGTTAGACAAGCCTTTATCAATTCTGGTGCATGGAAGATTGGTCTTGCTAATTTTGTGGGAATTATTGATAATCATTACCCAAAAACAAAGATCTTCCAGTTCTTAAAATTG 1200
HERV-K109       1076 AAGGAACAAGTTAGACAAGCCTTTATCAATTCTGGTGCATGGAAGATTGGTCTTGCTAATTTTGTGGGAATTATTGATAATCATTACCCAAAAACAAAGATCTTCCAGTTCTTAAAATTG 1195
HERV-K113       1081 AAGGAACAAGTTAGACAAGCCTTTATCAATTCTGGTGCATGGCAGATTGGTCTTGCTAATTTTGTGGGAATTATTGATAACCATTACCCAAAAACAAAGATCTTCCAGTTCTTAAAATTG 1200
HERV-K36        1081 AAGGAACAAGTTAGACAAGCCTTTATCAATTCTGGTGCATGGCAGATTGGTCTTGCTAATTTTGTGGGAATTATTGATAATCATTACCCAAAAACAAAGATCTTCCAGTTCTTAAAATTG 1200
HERV-K115       1081 AAGGAACAAGTTAGACAAGCCTTTATCAATTCTGGTGCATGGCAGATTGGTCTTGCTAATTTTGTGGGAATTATTGATAATCATTACCCAAAAACAAAGATCTTCCAGTTCTTAAAACTG 1200
HERV-K102       1081 AAGGAACAAGTTAGACAAGCCTTTATCAATTCTGGTGCATGGCAGATTGGTCTTGCTAATTTTGTGGGAATTATTGATAATCATTACCCAAAAACAAAGATCTTCCAGTTCTTAAAACTG 1200
HERV-K50A       1081 AAGGAACAAGTTAGACAAGCCTTTATCAATTCTGGTGCATGGCAGATTGGTCTTGCTAATTTTGTGGGAATTATTGATAATCATTACCCAAAAACAAAGATCTTCCAGTTCTTAAAACTG 1200
                     ******** ****  **** ******** ***** ***** * * ***** **** * *********** ******* ** ** ********* **** *********** ****** **

CERV-KOLD35587  1200 AATACTTGGATTTTACCTAAAATTACTAGACATAAACCTTTAGAAAATGCTCTGACAGTGTTTACTGATGGTTCCAGCAATGGAAAAGCGGCTTACACCAGGCCAAAAGAACGACTCATT 1319
HERV-KOLD35587  1200 ACTACTTGGATTTTACCTAAAATTACTAGACATAAACCTTTAGAAAATGCTCTGACAGTGTTTACTGATGGTTCCAGCAATGGAAAAGCGGCTTACACCAGGCCAAAAGAACGACTCATT 1319
SERV-K1 chr.11  1201 ACTACTTGGATTTTACCTAAAATTATCAGACATGAACCTCTAGAAAATGCTCTGACAGTATTTACTGATGGTTCCAGCAATGGAAAAGCAGCTTACACAGGGCCAAAAGAGCGAGTAATC 1320
Guereza         1201 ACTACTTGGATTTTACCTAAAATTACCAGACATAAACCTTTAGAAAATGCTCTGACAGTATTTACTGATGGTTCCAGCAATGGAAAAGCGGCTTACACAGGGCCAAAAGAGCGAGTAATC 1320
SERV-K1 chr.12  1201 ACTACTTGGATTTTACCTAAAATCACCAGACATGAGCCTTTAGAAAATGCTCTGACAGTATTTACTGATGGTTCCAGCAATGGAAAAGCGGCTTACACAGGACCAAAAGAGCGAGTAATC 1320
Grivet          1201 ACTACTTGGATTTTACCTAAAATTACCAGACATGAGCCTCTAGAAAATGCTCTGACAATATTTACTGATGGTTCCAGCAATGGAAAAGCAGCTTACACAGGACCAAAAGAGCGAGTAATC 1320
SERV-K1 chr.5   1196 ACTACTTGGATTTTACCTAAAATTACCAGACATGAACCTTTAGAAAATGCTCTGACAGTATTTACTGATGGTTCCAGCAATGGAAAAGCGGCTTACACAGGGCCAAAAGAGCGAGTAATC 1315
CERV-K60        1201 ACTACTTGGATTCTACCTAAAATTACCAGACACGAACCTTTAGAAAATGCTCTAACAGTATTTAGTGATGGTTCCAGCAATGGAAAAGTGGCTTACACAGGGCCAAAAGAACGAGTAATC 1320
CERV-K102       1201 ACTACTTGGATTCTACCTAAAATTACCAGACGTGAACCTTTAGAAAATGCTCTAACAGTATTTACTGATGGTTCCAGCAATGGAAAAGCGGCTTACACAGGGCCGAAAGAACGAGTAATC 1320
HERV-K104       1201 ACTACTTGGATTCTACCTAAAATTACCAGACATGAACCTTTAGAAAATGCTCTAACAGTATTTACTGATGGTTCCAGCAATGGAAAAGCAGCTTACACAGGGCCGAAAGAATGAGTAATC 1320
HERV-K50D       1201 ACTACTTGGATTCTACCTAAAATTACCAGACATGAACCTTTAGAAAATGCTCTAACAATATTTACTGATGGTTCCAGCAATGGAAAAGCAGCTTACACAGGGCCGAAAGAATGAGTAATC 1320
HERV-K50B       1201 ACTACTTGGATTCTACCTAAAATTACCAGACGTGAACCTTTAGAAAATGCTCTAACAGTATTTACTGATGGTTCCAGCAATGGAAAAGCAGCTTACACAGGGCCGAAAGAACGAGTAATC 1320
HERV-K106       1201 ACTACTTGGATTCTACCTAAAATTACCAGACGTGAACCTTTAGAAAATGCTCTAACAGTATTTACTGATGGTTCCAGCAATGGAAAAGCAGCTTACACAGGGCCGAAAGAACGAGTAATC 1320
HERV-K101       1200 ACTACTTGGATTCTACCTAAAATAACCAGACGTGAACCTTTAGAAAATGCTCTAACAGTATTTACTGATGGTTCCAGCAATGGAAAAGCAGCTTACACAGGGCCGAAAGAACGAGTAATC 1319
HERV-K103       1201 ACTACTTGGATTCTACCTAAAATTACCAGATGTGAACCTTTAGAAAATGCTCTAACAGTATTTACTGATGGTTCCAGCAATGGAAAAGCAGCTTACACAGGACCGAAAGAACGAGTAATC 1320
CERV-K100       1201 ACTACTTGGATTCTACCTAAAATTACCAGACGTGAACCTTTAGAAAATGCTCTAACAGTATTTACTGATGGTTCCAGCAATGGAAAAGCAGCTTACACAGGGCCGAAAGAACGAGTAATC 1320
HERV-K10        1201 ACTACTTGGATTCTACCTAAAATTACCAGACGTGAACCTTTAGAAAATGCTCTAACAGTATTTACTGATGGTTCCAGCAATGGAAAAGCAGCTTACACAGGGCCGAAAGAACGAGTAATC 1320
HERV-K108       1201 ACTACTTGGATTCTACCTAAAATTACCAGACGTGAACCTTTAGAAAATGCTCTAACAGTATTTACTGATGGTTCCAGCAATGGAAAAGCAGCTTACACAGGACCGAAAGAACGAGTAATC 1320
HERV-K109       1196 ACTACTTGGATTCTACCTAAAATTACCAGACGTGAACCTTTAGAAAATGCTCTAACAGTATTTACTGATGGTTCCAGCAATGGAAAAGCAGCTTACACAGGGCCGAAAGAACGAGTAATC 1315
HERV-K113       1201 ACTACTTGGATTCTACCTAAAATTACCAGACGTGAACCTTTAGAAAATGCTCTAACAGTATTTACTGATGGTTCCAGCAATGGAAAAGCAGCTTACACAGGGCTGAAAGAACGAGTAATC 1320
HERV-K36        1201 ACTACTTGGATTCTACCTAAAATTACCAGACGTGAACCTTTAGAAAATGCTCTAACAGTATTTACTGATGGTTCCAGCAATGGAAAAGCAGCTTACACAGGGCCGAAAGAACGAGTAATC 1320
HERV-K115       1201 ACTACTTGGATTCTACCTAAAATTACCAGACGTGAACCTTTAGAAAATGCTCTAACAGTATTTACTGATGGTTCCAGCAATGGAAAAGCAGCTTACACAGGGCCGAAAGAACGAGTAATC 1320
HERV-K102       1201 ACTACTTGGATTCTACCTAAAATTACCAGACGTGAACCTTTAGAAAATGCTCTAACAGTATTTACTGATGGTTCCAGCAATGGAAAAGCAGCTTACACAGGACCGAAAGAACGAGTAATC 1320
HERV-K50A       1201 ACTACTTGGATTCTACCTAAAATTACCAGACGTGAACCTTTAGAAAATGCTCTAACAGTATTTACTGATGGTTCCAGCAATGGAAAAGCAGCTTACACAGGACCGAAAGAACGAGTAATC 1320
                     * ********** ********* *   ***     * *** ************* *** * **** **********************   ********   * *   *****   ** * **

CERV-KOLD35587  1320 GAAACTCAATATCACTGGGCTCAAAGAGCAGAGTTGGTTGCTGTCATTATAGTGTTACAAGATTTTAATCAGCCTATTAACATTGTATCAGATTCTGCATATGTAGTACAGGCTACAAAG 1439
HERV-KOLD35587  1320 GAAACTCAATATCACTGGGCTCAAAGAGCAGAGTTGGTTGCTGTCATTACAGTGTTACAAGATTTTAATCAGCCTATTAACATTGTATCAGATTCTGCATATGTAGTACAGGCTACAAAG 1439
SERV-K1 chr.11  1321 AAAACTCAATATCAATCGGCTCAAAGGGCAGAGTTGGTTGCAGTCATTACAGTGTTACAAGATTTTAAGCAACCTGTTAATATTATATCAGATTCTGCATATGTAGTACAGGCTACAAGG 1440
Guereza         1321 AAAACTCAATATCAATCAGCTCAAAGGGCAGAGTTGGTTGCAGTCATTACAGTGTTAGAAGATTTTAATCAACCTGTTAATATTATATCAGATTCTGCATATGTAGTACAGGCTACAAGG 1440
SERV-K1 chr.12  1321 AAAACTCAGTATCAATCAGCTCAAAGAGCAGAGTTGGTTGCAGTCATTACAGTGTTACAAGATTTTAATCAACCTGTTAATATTATATCAGATTCTGCATATGTAGTACAGGCTACAAGG 1440
Grivet          1321 AAAACTCAGTATCAATCAGCTCAAAGGGCAGAGTTGGTTGCAGTCATTACAGTGTTACAAGATTTTAATCAACCTGTTAATATTATATCAGATTCTGCATATGTAGTACAGGCTACAAAG 1440
SERV-K1 chr.5   1316 AAAACTCAATATCAATCG--ATATTGGGCAGAGTTGGTTGCAGTCATTACAGTGTTACAAGATTGTAATCAACCTGTTAATATTGTATCAGATTCTGCATATGTAGTACAGGCTACAAGG 1433
CERV-K60        1321 AAAACTCCATATTAATCGGCTCAAAGAGCAGAGTTGGTTGCAGTCATTACAGTGTTACAAGATTTTGATCAACCTATCAATATTATATCAGATTCTGCATATGTAGTACAGGCTACAAGG 1440
CERV-K102       1321 AAAACTCCGTATCAATCGGCTCAAAGAGCAGAGTTGGTTGCAGTCATTACAGTGTTACAAGATTTTGACCAACCTATCAATATTATATCAGATTCTGCATATGTAGTACAGGCTACAAGG 1440
HERV-K104       1321 AAAACTCCATATCAATCAGCTCAAAGAGCAGAGTTGGTTGCAGTCATTACAGTGTTACAAGATTTTGACCAACCTATCAATATTATATCAGATTCTGCATATGTAGTACAGGCTACAAGG 1440
HERV-K50D       1321 AAAACTCCATATCAATCAGCTCAAAGAGCAGAGTTGGTTGCAGTCATTAAAGTGTTACAAGATTTTGACCAACCTATCAATATTATATCAGATTCTGCATATGTAGTACAGGCTACAAGG 1440
HERV-K50B       1321 AAAACTCCATATCAATCTGCTCAAAGAGCAGAGTTGGTTGCAGTCATTACAGTGTTACAAGATTTTGACCAACCTATCAATATTATATCAGATTCTGCCTATGTAGTACAGGCTACAAGG 1440
HERV-K106       1321 AAAACTCCATATCAATCAGCTCAAAGAGCAGAGTTGGTTGCAGTCATTACAGTGTTACAAGATTTTGACCAACCTATCAATATTATATCAGATTCTGCATATGTAGTACAGGCTACAAGG 1440
HERV-K101       1320 AAAACTCCATATCAGTCGGCTCAAAGAGCAGAGTTGGTTGCAGTCATTACAGTGTTACAAGATTTTGACCAACCTATCAATATTATATCAGATTCTGCCTATGTAGTACAGGCTACAAGG 1439
HERV-K103       1321 AAAACTCCATATCAATCGGCTCAAAGAGCAGAGTTGGTTGCAGTCATTACAGTGTTACAAGATTTTGACCAACCTATCAATATTATATCAGATTCTGCATATGTAGTACAGGCTACAAGG 1440
CERV-K100       1321 AAAACTCCATATCAATCGGCTCAAAGAGACGAGTTGGTTGCAGTCATTACAGTGTTACAAGATTTTGACCAACCTATCAATATTATATCAGATTCTGCATATGTAGTACAGGCTACAAGG 1440
HERV-K10        1321 AAAACTCCATATCAATCGGCTCAAAGAGACGAGTTGGTTGCAGTCATTACAGTGTTACAAGATTTTGACCAACCTATCAATATTATATCAGATTCTGCATATGTAGTACAGGCTACAAGG 1440
HERV-K108       1321 AAAACTCCATATCAATCGGCTCAAAGAGCAGAGTTGGTTGCAGTCATTACAGTGTTACAAGATTTTGACCAACCTATCAATATTATATCAGATTCTGCATATGTAGTACAGGCTACAAGG 1440
HERV-K109       1316 AAAACTCCATATCAATCGGCTCAAAGAGCAGAGTTGGTTGCAGTCATTACAGTGTTACAAGATTTTGACCAACCTATCAATATTATATCAGATTCTGCATATGTAGTACAGGCTACAAGG 1435
HERV-K113       1321 AAAACTCCATATCAATCGGCTCAAAGAGCAGAGTTGGTTGCAGTCATTACAGTGTTACAAGATTTTGACCAGCCTATCAATATTATATCAGATTCTGCATATGTAGTACAGGCTACAAGG 1440
HERV-K36        1321 AAAACTCCATATCAATCGGCTCAAAGAGCAGAGTTGGTTGCAGTCATTACAGTGTTACAAGATTTTGA------TATCAATATTATATCAGATTCTGCCTATGTAGTACAGGCTACAAGG 1434
HERV-K115       1321 AAAACTCCATATCAATCAGCTCAAAGAGCAGAGTTGGTTGCAGTCATTACAGTGTTACAAGATTTTGACCAACCTATCAATATTATATCAGATTCTGCATATGTAGTACAGGCTACAAGG 1440
HERV-K102       1321 AAAACTCCATATCAATCGGCTCAAAGAGCAGAGTTGGTTGCAGTCATTACAGTGTTACAAGATTTTGACCAACCTATCAATATTATATCAGATTCTGCATATGTAGTACAGGCTACAAGG 1440
HERV-K50A       1321 AAAACTCCATATCAATCGGCTCAAAGAGCAGAGTTGGTTGCAGTCATTACAGTGTTACAAGATTTTGACCAACCTATCAATATTATATCAGATTCTGCATATGTAGTACAGGCTACAAGG 1440
                      ******  *** * *      *  * *  *********** ******* ******* ****** * *      * * ** *** ************ ****************** *
```

```
CERV-KOLD35587   1440 GATGTTGAGACAGCCCTAATCAAATATAGTATAGATGATCAGTTAAACCAGCTGTTTAATATGTTACAACAAACTGTAAGAAAAAGAAATTTCCCATTTTATGTTACTCATATTCGAGCA 1559
HERV-KOLD35587   1440 GATGTTGAGACAGCCCTAATCAAATATAGTATAGATGATCAGTTAAACCAGCTGTTTAATATGTTACAACAAACTGTAAGAAAAAGAAATTTCCCATTTTATGTTACTCATATTCGAGCA 1559
SERV-K1 chr.11   1441 GATGTTGAGACAGCTCTAATTAAATATAGCATGGATGATCAGTTAAACCAGCTGTTCAATTTACTACAACAAACTGTAAGAAAAAGGAATTTCCCATTTTATATTACTCATATTCGAGCA 1560
Guereza          1441 GATGTTGAGACAGCTCTAATTAAATATAGCATGGATGATCAGTTAAACCAGCTATTCACTTTGTTACAACAAACTGTAAGAAAGAGAAATTTCCCATTTTATATTACTCATATTCGAGCA 1560
SERV-K1 chr.12   1441 GATGTTGAGACAGCTCTAATTAAATATAGCATGGATGATCAGTTAAACCAGTTGTTTAATTTATTACAACAAACTGTAAGAAAAAAGAATTTCTCATTTTATATTACTCATATTCGAGCA 1560
Grivet           1441 GATGTTGAGACAGCTCTAATTAAATATAGCATGGATGATCAGTTAAACCAGTTGTTCAATTTATTACAACAAACTGTAAGAAAAAGGAATTTCCCATTTTATATCACTCATATTCGAGCA 1560
SERV-K1 chr.5    1434 GATGTTGAGACAGCTCTAATTAAATACAGCATGGATGATCAGTTAAACCAGCTGTTCAATTTATTACAACAAACTGTAAGAAAAAGGAATTTCCCATTTTATATTACTGGTATTCGAGCA 1553
CERV-K60         1441 GATGTTGAGACAGCTCTAATTAAATATAGCATGGACGATCAGTTAAACCAGCTATTCAATTTATTACAACAAACTGTAAGAAAAAGAAACTTCCCATTTTATATTACTCATATTCGAGCA 1560
CERV-K102        1441 GATGTTGAGACAGCTCTAATTAAATATAGCATGGATGATCAGTTAAACCAGCTATTCAATTTATTACAACAAACTGTAAGAAAAAGAAATTTCCCATTTTATATTACTCATATTCGAGCA 1560
HERV-K104        1441 GATGTTGAGACAGCTCTAATTAAATATAGCATGGATGATCAGTTAAACCAGCTATTCAATTTATTACAACAAACTGTAAGAAAAAGAAATTTCCCATTTTATATTACTCATATTCGAGCA 1560
HERV-K50D        1441 GATGTTGAGACAGCTCTAATTAAATATAGCATGGATGATCAGTTAAACCAGCTATTCAATTTATTACAACAAACTGTAAGAAAAAGAAATTTCCCATTTTATATTACTCATATTCGAGCA 1560
HERV-K50B        1441 GATGTTGAGACAGCTCTAATTAAATATAGCATGGATGATCAGTTAAACCAGCTATTCAATTTATTACAACAAACTGTAAGAAAAAGAAATTTCCCATTTTATATTACTCATATTCGAGCA 1560
HERV-K106        1441 GATGTTGAGACAGCTCTAATTAAATATAGCATGGATGATCAGTTAAACCAGCTATTCAATTTATTACAACAAACTGTAAGAAAAAGAAATTTCCCATTTTATATTACTCATATTCGAGCA 1560
HERV-K101        1440 GATGTTGAGACAGCTCTAATTAAATATAGCATGGATGATCAGTTAAACCAGCTATTCAATTTATTACAACAAACTGTAAGAAAAAGAAATTTCCCATTTTATATTACTCATATTCGAGCA 1559
HERV-K103        1441 GATGTTGAGACAGCTCTAATTAAATATAGCATGGATGATCAGTTAAACCAGCTATTCAATTTATTACAACAAACTGTAAGAAAAAGAAATTTCCCATTTTATATTACTCATATTCGAGCA 1560
CERV-K100        1441 GATGTTGAGACAGCTCTAATTAAATATAGCATGGATGATCAGTTAAACCAGCTATTCAATTTATTACAACAAACTGTAAGAAAAAGAAATTTCCCATTTTATATTACTTATATTCGAGCA 1560
HERV-K10         1441 GATGTTGAGACAGCTCTAATTAAATATAGCATGGATGATCAGTTAAACCAGCTATTCAATTTATTACAACAAACTGTAAGAAAAAGAAATTTCCCATTTTATATTACTTATATTCGAGCA 1560
HERV-K108        1441 GATGTTGAGACAGCTCTAATTAAATATAGCATGGATGATCAGTTAAACCAGCTATTCAATTTATTACAACAAACTGTAAGAAAAAGAAATTTCCCATTTTATATTACACATATTCGAGCA 1560
HERV-K109        1436 GATGTTGAGACAGCTCTAATTAAATATAGCATGGATGATCAGTTAAACCAGCTATTCAATTTATTACAACAAACTGTAAGAAAAAGAAATTTCCCATTTTATATTACTCATATTCGAGCA 1555
HERV-K113        1441 GATGTTGAGACAGCTCTAATTAAATATAGCATGGATGATCAGTTAAACCAGCTATTCAATTTATTACAACAAACTGTAAGAAAAAGAAATTTCCCATTTTATATTACTCATATTCGAGCA 1560
HERV-K36         1435 GATGTTGAGACAGCTCTAATTAAATATAGCATGGATGATCAGTTAAACCAGCTATTCAATTTATTACAACAAACTGTAAGAAAAAGAAATTTCCCATTCTATATTACTCATATTCGAGCA 1554
HERV-K115        1441 GTTGTTGAGACAGCTCTAATTAAATATAGCATGGATGATCAGTTAAACCAGCTATTCAATTTATTACAACAAACTGTAAGAAAAAGAAATTTCCCATTTTATATTACTCATATTCGAGCA 1560
HERV-K102        1441 GATGTTGAGACAGCTCTAATTAAATATAGCATGGATGATCAGTTAAACCAGCTATTCAATTTATTACAACAAACTGTAAGAAAAAGAAATTTCCCATTTTATATTACTCATATTCGAGCA 1560
HERV-K50A        1441 GATGTTGAGACAGCTCTAATTAAATATAGCATGGATGATCAGTTAAACCAGCTATTCAATTTATTACAACAAACTGTAAGAAAAAGAAATTTCCCATTTTATATTACTCATATTCGAGCA 1560
                      * ************ ***** ***** ** ** ** *************** * ** * * *  ******************* *  ** *** **** *** * **   **********

CERV-KOLD35587   1560 CATACTAATTTATCAGAGCCTTTAACTAAAGCAAATGAACAAGTTGACTTGCTAGTATCATCTGCATTCCTGGAAGCACAAGAACTTCATGCCTTGACTCATGTAAATGCAACAGGACTA 1679
HERV-KOLD35587   1560 CATACTAATTTACCAGAGCCTTTAACTAAAGCAAATGAACAAGTTGACTTGCTAGTATCATCTGCATTCCTGGAAGCACAAGAACTTCATGCCTTGACTCATGTAAATGCAACAGGACTA 1679
SERV-K1 chr.11   1561 CACACTAATTTACCAGGACCTTTAACTAAAGCAAATGAACAAGCTGACTTACTGGTATCCTCTGCATTCATAAAAGCACAAGAACTTCATGCTTTGACTCATGTAAATGCAGCAGGGTTA 1680
Guereza          1561 CACACTAATTTACCAGGACCTTTAACTAAAGCTAATGAAAAGGCTGACTTACTGGTATCCTCTGCATTCATAAAAGCACAAGAACTTCATGCTTTGACTCATGTAAATGCAGCAGGGTTA 1680
SERV-K1 chr.12   1561 CACACTAATTTGCCAGGACCTTTAACTATAGCAAATGAACAAGCTGACTTACTGGTATCCTCTGCATTCATAAAAGCACAAGAACTTCATGCTTTGACTCATGTAAATGCAGCAGGGTTA 1680
Grivet           1561 CACACTAATTTACCAGGACCTTAAACTATAGCAAATGAACAAGCTGACTTACTGGTATCCTCTGCATTCTTAAAAGCACAAGAACTTCATGCTTTAACTCATGTAAATGCAGCAGGGTTA 1680
SERV-K1 chr.5    1554 CACACTAATTTACCGGGACCTTTAACTAAAGGAAATGAACAAGCTGACTTACTAGTATCATATGCATTCATAAAAGCATAAGAACTTCATGTTTTAACTCATGTAAATGCAGCAGGGTTA 1673
CERV-K60         1561 CACACTAATTTACCAGGGCCTTTGACTAAAGCAAATGAACAAGCTGACTTACTGGTGTCATCTGCATTCATAAAAGCACAAGAACTTCATGCTTTGACTCATGTAAATGCAGCAGGATTA 1680
CERV-K102        1561 CACACTAATTTACCAGGGCCTTTGACTAAAGCAAATGAACAAGCTGACTTACTGGTATCATCTGCATTCATAAAAGCACAAGAACTTCATGCTTTGACTCACGTAAATGCAGCAGGATTA 1680
HERV-K104        1561 CACACTAATTTACCAGGGCCTTTGACTAAAGCAAATGAACAAGCTGACTTACTGGTATCATCTGCATTCATAAAAGCACAAGAACTTCATGCTTTGACTCATGTAAATGCAGCAGGATTA 1680
HERV-K50D        1561 CACACTAATTTACCAGGGCCTTTGACTAAAGCAAATGAACAAGCTGACTTACTGGTATCATCTGCATTCATAAAAGCACAAGAACTTCATGCTTTGACTCATGTAAATGCAGCAGGATTA 1680
HERV-K50B        1561 CACACTAATTTACCAGGGCCTTTGACTAAAGCAAATGAAGAAGCTGACTTACTGGTATCATCTGCACTCATAAAAGCACAAGAACTTCATGCTTTGACTCATGTAAATGCAGCAGGATTA 1680
HERV-K106        1561 CACACTAATTTACCAGGGCCTTTGACTAAAGCAAATGAAGAAGCTGACTTACTGGTATCATCTGCACTCATAAAAGCACAAGAACTTCATGCTTTGACTCATGTAAATGCAGCAGGATTA 1680
HERV-K101        1560 CACACTAATTTACCAGGGCCTTTGACTAAAGCAAATGAACAAGCTGACTTACTGGTATCATCTGCACTCATAAAAGCACAAGAACTTCATGCTTTGACTCATGTAAATGCAGCAGGATTA 1679
HERV-K103        1561 CACACTAATTTACCAGGGCCTTTGACTAAAGCAAATGAACAAGCTGACTTACTGGTATCATCTGCACTCATAAAAGCACAAGAACTTCATGCTTTGACTCATGTAAATGCAGCAGGATTA 1680
CERV-K100        1561 CACACTAATTTACCAGGGCCTTTGACTAAAGCAAATGAACAAGCTGACTTACTGGTATCATCTGCACTCATAAAAGCACAAGAACTTCATGCTTTGACTCATGTAAATGCAGCAGGATTA 1680
HERV-K10         1561 CACACTAATTTACCAGGGCCTTTGACTAAAGCAAATGAACAAGCTGACTTACTGGTATCATCTGCACTCATAAAAGCACAAGAACTTCATGCTTTGACTCATGTAAATGCAGCAGGATTA 1680
HERV-K108        1561 CACACTAATTTACCAGGGCCTTTGACTAAAGCAAATGAACAAGCTGACTTACTGGTATCATCTGCACTCATAAAAGCACAAGAACTTCATGCTTTGACTCATGTAAATGCAGCAGGATTA 1680
HERV-K109        1556 CACACTAATTTACCAGGGCCTTTGACTAAAGCAAATGAACAAGCTGACTTACTGGTATCATCTGCACTCATAAAAGCACAAGAACTTCATGCTTTGACTCATGTAAATGCAGCAGGATTA 1675
HERV-K113        1561 CACACTAATTTACCAGGGCCTTTGACTAAAGCAAATGAACAAGCTGACTTACTGGTATCATCTGCACTCATAAAAGCACAAGAACTTCATGCTTTGACTCATGTAAATGCAGCAGGATTA 1680
HERV-K36         1555 CACACTAATTTACCAGGGCCTTTGACTAAAGCAAATAAACAAGCTGACTTACTGGTATCATCTGCACTCATAAAAGCACAAGAACTTCATGCTTTGACTCATGTAAATGCAGCAGGATTA 1674
HERV-K115        1561 CACACTAATTTACCAGGGCCTTTGACTAAAGCAAATGAACAAGCTGACTTACTGGTATCATCTGCACTCATAAAAGCACAAGAACTTCATGCTTTGACTCATGTAAATGCAGCAGGATTA 1680
HERV-K102        1561 CACACTAATTTACCAGGGCCTTTGACTAAAGCAAATGAACAAGCTGACTTACTGGTATCATCTGCACTCATAAAAGCACAAGAACTTCATGCTTTGACTCATGTAAATGCAGCAGGATTA 1680
HERV-K50A        1561 CACACTAATTTACCAGGGCCTTTGACTAAAGCAAATGAACAAGCTGACTTACTGGTATCATCTGCACTCATAAAAGCACAAGAACTTCATGCTTTGACTCATGTAAATGCAGCAGGATTA 1680
                      ** ********  * *  ****  **** **  *** ** * * ****** ** ** ** * **** ** *  ***** ************  ** ***** ********* ****  **

CERV-KOLD35587   1680 AAAAATAAATTTGACATCACATGGAAACAGGCAAAAAATATTGTACAACATTACACCCAGTGTCAGATTCCA---CACCTGCCCACTCAGGAGGCAGGAGTTAATCCCAGAGGTCTGTGT 1796
HERV-KOLD35587   1680 AAAAATAAATTTGACATCACATGGAAACAGGCAAAAAATATTGTACAACATTACACCCAGTGTCAGATTCCA---CACCTGCCCACTCAGGAGGCAGGAGTTAATCCCAGAGGTCTGTGT 1796
SERV-K1 chr.11   1681 AAAAACAAATTTAATGTCACATGGAAACAGGCAAAAGATATAGTACAACATTGCACCCAGTGTCAAGTACTA---CACCTGCCCACTCAAGAGGCAGGAGTTAATCCCAGAGGTCTGTGT 1797
Guereza          1681 AAAAACAAATTTGATGTCACATGGAAACAGGCAAAAAATATTGTACAACATTGTACCCAGTGTCAAGTACTA---CACCTGCCCACCCAAGAGGCAGGAGTTAACCCCAGAGGTCTGTGT 1797
SERV-K1 chr.12   1681 AAAAACAAATTTGATGTCACATGGAAACAGGCAAAAGATATTGTACGACATTGCACCCAGTGTCAAGTACTA---CACCTGCCCACTCAAGAGGCAGGAGTGAATCCCAGAGGTCTGTGT 1797
Grivet           1681 AAAAACAAATTTGATGTCACATGGAAACAGGCAAAAGATATCGTACAACACTGCACCCAGTGTCAAGTACTA---CACCTGCCCACTCAAGAGGCAGGAGTTAATCCCAGAGGTCTGTGT 1797
SERV-K1 chr.5    1674 AAAAACAAATTTGATGTCACTTGGAAACAGGCAAAAGATATTGTACAACCCTGCACCCAATGTTAAGTCCTA---CACCTGCCCACTCAAGAGGCAGGAGTTAATCCCAGAGGTCTGTGT 1790
CERV-K60         1681 AAAAACAAATTTGATGTCACATGGAAACAGGCAAAAGATATTGTACAACATTGCACCCAGTGTCAAGTCTTA---CACCTGCCCACTCAAGAGGCAGGAGTTAATCCCAGAGGTCTGTGT 1797
CERV-K102        1681 AAAAACAAATTTGATGTCACATGGAAATAGGCAAAAGATATTGTACAACATTGCACCCAGTGTCAAGTCTTA---CACCTGCCCACTCAAGAGGCAGGAGTTAATCCCAGAGGTCTGTGT 1797
HERV-K104        1681 AAAAACAAATTTGATGTCACATGGAAACAGGCAAAAGATATTGTACAACATTGCACCCAGTGTCAAGTCTTA---CACCTGCCCACTCAAGAGGCAGGAGTTAATCCCAGAGGTCTGTGT 1797
HERV-K50D        1681 AAAAACAAATTTGATGTCACATGGAAACAGGCAAAAGATATTGTACAACATTGCACCCAGTGTCAAGTCTTA---CACCTGCCCACTCAAGAGGCAGGAGTTAATCCCAGAGGTCTGTGT 1797
HERV-K50B        1681 AAAAACAAATTTGATGTCACATGGAAACAGGCAAAAGATATTGTACAACATTGCACCCAGTGTCAAGTCTTA---CACCTGCCCACTCAAGAGGCAGGAGTTAATCCCAGAGGTCTGTGT 1797
HERV-K106        1681 AAAAACAAATTTGATGTCACATGGAAACAGGCAAAAGATATTGTACAACATTGCACCCAGTGTCAAGTCTTA---CACCTGCCCACTCAAGAGGCAGGAGTTAATCCCAGAGGTCTGTGT 1797
HERV-K101        1680 AAAAACAAATTTGATGTCACATGGAAACAGGCAAAAGATATTGTACAACATTGCACCCAGTGTCAAGTCTTA---CACCTGCCCACTCAAGAGGCAGGAGTTAATCCCAGAGGTCTGTGT 1796
HERV-K103        1681 AAAAACAAATTTGATGTCACATGGAAACAGGCAAAAGATATTGTACAACATTGCACCCAGTGTCAAGTCTTA---CACCTGCCCACTCAAGAGGCAGGAGTTAATCCCAGAGGTCTGTGT 1797
CERV-K100        1681 AAAAACAAATTTGATGTCACATGGAAACAGGCAAAAGATATTGTACAACATTGCACCCAGTGTCAAGTCTTA---CACCTGCCCACTCAAGAGGCAGGAGTTAATCCCAGAGGTCTGTGT 1797
HERV-K10         1681 AAAAACAAATTTGATGTCACATGGAAACAGGCAAAAGATATTGTACAACATTGCACCCAGTGTCAAGTCTTA---CACCTGCCCACTCAAGAGGCAGGAGTTAATCCCAGAGGTCTGTGT 1797
HERV-K108        1681 AAAAACAAATTTGATGTCACATGGAAACAGGCAAAAGATATTGTACAACATTGCACCCAGTGTCAAGTCTTA---CACCTGCCCACTCAAGAGGCAGGAGTTAATCCCAGAGGTCTGTGT 1797
HERV-K109        1676 AAAAACAAATTTGATGTCACATGGAAACAGGCAAAAGATATTGTACAACATTGCACCCAGTGTCAAGTCTTA---CACCTGCCCACTCAAGAGGCAGGAGTTAATCCCAGAGGTCTG--- 1789
HERV-K113        1681 AAAAACAAATTTGATGTCACATGGAAACAGGCAAAAGATATTGTACAACATTGCACCCAGTGTCAAGTCTTA---CACCTGCCCACTCAAGAGGCAGGAGTTAATCCCAGAGGTCTGTGT 1797
HERV-K36         1675 AAAAACAAATTTGATGTCACATGGAAACTGGCAAAAGATATTGTACAACATTGCACCCAGTGTCAAGTCTTA---CACCTGCCCACTCAAGAGGCAGGAGTTAATCCCAGAGGTCTGTGT 1791
HERV-K115        1681 AAAAACAAATTTGATGTCACATGGAAACAGGCAAAAGATATTGTACAACATTGCACCCAGTGTCAAGTCTTA---CACCTGCCCACTCAAGAGGCAGGAGTTAATCCCAGAGGTCTGTGT 1797
HERV-K102        1681 AAAAACAAATTTGATGTCACATGGAAACAGGCAAAAGATATTGTACAACATTGCACCCAGTGTCAAATCTTA---CACCTGCCCACTCAAGAGGCAGGAGTTAATCCCAGAGGTCTGTGT 1797
HERV-K50A        1681 AAAAACAAATTTGATGTCACATGGAAACAGGCAAAAGATATTGTACAACATTGCACCCAGTGTCAAATCTTA---CACCTGCCCACTCAAGAGGCAGGAGTTAATCCCAGAGGTCTGTGT 1797
                      ***** ****** *  **** ******  ******* **** **** **  *  ***** *** *  *   *************** ** *********** ** ************

CERV-KOLD35587   1797 CCTAATGCGTTATGGCAAATGGATGTCACACATGTACCTTCAGTTGGAAAATTGTCATTTCTCCATGTGACAGTTGATGCTTATTCACATTTTGTAAGGGCAAACTGCCAGACAGGAGAA 1916
HERV-KOLD35587   1797 CCTAATGCGTTATGGCAAATGGATGTCACACATGTACCTTCAGTTGGAAAATTGTCATTTCTCCATGTGACAGTTGATGCTTATTCACATTTTGTAAGGGCAAACTGCCAGACAGGAGAA 1916
SERV-K1 chr.11   1798 CCTAATGCATTATGGCAAATGGATGTTACACATGTACCTTCATTTGGAAAATTATCATATGTTCATGTAACAGTTGATACTTATTCACATTTCATATGGGCAGCCTGCCAGACAGGAGAA 1917
Guereza          1798 CCTAATGCCTTATGGCAAATGGATGTTACCCATGTACCTTCATTTGGAAAATTATCATATGTTCATGTAACAGTTAATACTTATTCACATTTCATATGGGCAACATGCCAGACAGGAGAA 1917
SERV-K1 chr.12   1798 CCTAATGCATTATGGCAAATGGATGTTACCCATGTACCTTCATTTGGAAAATTATCATATGTTCATGTAACAGTTGATACTTATTCACATTTCATATGGGCAACATGCCAGACAGGAGAA 1917
Grivet           1798 CCTAATGCATTATGGCAAATGGATGTTACCCATGTACCTTCATTTGGAAAATTATCATATGTTCATGTAACAGTTGATACTTATTCACATTTCATATGGGCAACATGCCAGACAGGAGAA 1917
SERV-K1 chr.5    1791 CCTAACGCATTATGGCAAATGTATGTTACACATGTACCTTCATTTGGAAAATTATCATACGTTCATGAAACAGTTGATACTTATTCACATTTCATATGGGCAACCTGCTAGACAGGAGAA 1910
CERV-K60         1798 CCTAATGCATTATGGCAAATGGATGTCACGCATGTACCTTCATTTGGAAGATTATCATATGTTCATGTAACAGTTGATACTTATTCACATTTCATATGGGCAACTTGCCAAACAGGAGAA 1917
CERV-K102        1798 CCTAATGCATTATGGCAAATGGATGTCACGCATGTACCTTCATTTGGAAAATTATCACATGTTCATGTAACCGTTGATACTTATTCACATTTCATATGGGCAACTTGCCAAACAGGAGAA 1917
HERV-K104        1798 CCTAATGCATTATGGCAAATGGATGTCACGCATGTACCTTCATTTGGAAGATTATCATATGTTCATGTAACAGTTGATACTTATTCACATTTCATATGGGCAACTTGCCAAACAGGAGAA 1917
HERV-K50D        1798 CCTAATGCATTATGGCAAGTGGATGTCACGCATGTACCTTCATTTGGAAGATTATCATATGTTCATGTAACAGTTGATACTTATTCACATTTCATATGGGCAACTTGCCAAACAGGAGAA 1917
HERV-K50B        1798 CCTAATGCATTATGGCAAATGGATGTCACGCATGTACCTTCATTTGGAAGATTATCGTATGTTCATGTAACAGTTGATACTTATTCACATTTCATATGGGCAACTTGCCAAACAGGAGAA 1917
HERV-K106        1798 CCTAATGCATTATGGCAAATGCATGTCACGCATGTACCTTCATTTGGAAGATTATCATATGTTCATGTAACAGTTGATACTTATTCACATTTCATATGGGCAACTTGCCAAACAGGAGAA 1917
HERV-K101        1797 CCTAATGCATTATGGCAAATGGATGTCACGCATGTACCTTCATTTGGAAGATTATCATATGTTCATGTAACAGTTGATACTTATTCACATTTCATATGGGCAACTTGCCAAACAGGAGAA 1916
HERV-K103        1798 CCTAATGCATTATGGCAAATGGATGTCACGCATGTACCTTCATTTGGAAGATTATCATATGTTCATGTAACAGTTGATACTTATTCACATTTCATATGGGCAACTTGCCAAACAGGAGAA 1917
CERV-K100        1798 CCTAATGCATTATGGCAAATGGATGTCACGCATGTACCTTCATTTGGAAGATTATCATATGTTCATGTAACAGTTGATACTTATTCACATTTCATATGGGCAACTTGCCAAACAGGAGAA 1917
HERV-K10         1798 CCTAATGCATTATGGCAAATGGATGTCACGCATGTACCTTCATTTGGAAGATTATCATATGTTCATGTAACAGTTGATACTTATTCACATTTCATATGGGCAACTTGCCAAACAGGAGAA 1917
HERV-K108        1798 CCTAATGCATTATGGCAAATGGATGTCACGCATGTACCTTCATTTGGAAGATTATCATATGTTCACGTAACAGTTGATACTTATTCACATTTCATATGGGCAACTTGCCAAACAGGAGAA 1917
HERV-K109        1790 -----------------------TGTCACGCATGTACCTTCATTTGGAAGATTATCATATGTTCATGTAACAGTTGATACTTATTCACATTTCATATGGGCAACTTGCCAAACAGGAGAA 1886
HERV-K113        1798 CCTAATGCATTATGGCAAATGGATGTCACACATGTACCTTCATTTGGAAGATTATCATATGTTCATGTAACAGTTGATACTTATTCACATTTCATATGGGCAACTTGCCAAACAGGAGAA 1917
HERV-K36         1792 CCTAATGCATTATGGCAAATGGATGTCACGCATGTACCTTCATTTGGAAGATTATCATATGTTCATGTAACAGTTGATACTTATTCACATTTCATATGGGCAACTTGCCACACAGGAGAA 1911
HERV-K115        1798 CCTAATGCATTATGGCAAATGGATGTCACGCATGTACCTTCATTTGGAAGATTATCATATGTTCATGTAACAGTTGATACTTATTCACATTTCATATGGGCAACTTGCCAAACAGGAGAA 1917
HERV-K102        1798 CCTAATGCATTATGGCAAATGGATGTCACGCATGTACCTTCATTTGGAAGATTATCATATGTTCACGTAACAGTTGATACTTATTCACATTTCATATGGGCAACTTGCCAAACAGGAGAA 1917
HERV-K50A        1798 CCTAATGCATTATGGCAAATGGATGTCACGCATGTACCTTCATTTGGAAGATTATCATATGTTCACGTAACAGTTGATACTTATTCACATTTCATATGGGCAACTTGCCAAACAGGAGAA 1917
                                             *** ** ************ ****** *** **     * ** *  ** *** ** *************  ** *****   *** * *********
```

```
CERV-KOLD35587  1917 AATACTTCCCATGTTAAAAGACATTTAGTATCTTGTTTTGCTGTCATGGGAGTTCCAGAAAAAATTAAAACGCATAATGGGCCAGGATACTGTAGTAAGACATTTCAAAAATTCTTAAAT 2036
HERV-KOLD35587  1917 AATACTTCCCATGTTAAAAGACATTTATTATCTTGTTTTGCTGTCATGGGAGTTCCAGAAAAAATTAAAACGGATAATGGGCCAGGATACTGTAGTAAAACATTTCAAAAATTCTTAAAT 2036
SERV-K1 chr.11  1918 AGTACTTCCCATGTTAAAAAACACTTATTATCTTGCTTTGCTGTAATGGGAGTTCCAGAAAAAATTAAAACTGACAATGGACCAAGATATTGTAGTAAAGCTTTCCAAAAATTCTTAAAT 2037
Guereza         1918 AGTACTTCCCATGTTAAAAAACATTTATTATCTTGTTTTGCTGTAATGGGAGTTCCAGAAAAAATTAAAACTGACAATGGACCAGGTTATTGTAGTAAAGCTTTCCAAAAATTCTTAAAT 2037
SERV-K1 chr.12  1918 AGTACTTCCCATGTTAAAAAACATTTATTATCTTGTTTTGCTGTAATGGGAGTTCCAGAAAAAATTAAAACTGACAATGGACCAGGTTATTGTAGTAAAGCTTTCCAAAAATTCTTAAAT 2037
Grivet          1918 AGTACTTCCCATGTTAAAAAACATTTATTATCTTGTTTTGCTGTAATGGGAGTTCCAGAAAAAATTAAAACTGACAATGGACCAGGTTATTGTAGTAAAGCTTTCCAAAAATTCTTAAAT 2037
SERV-K1 chr.5   1911 AGTACTTCCCATGTTAAAAAACATTCATTATCTTGTTTTGCTGTAATGGGAGTTCCAGAAAAAATTAAAACTGACAATGGACCAGGATATTGTAGTAAAGCTTTACAAAAATTCTTAAAT 2030
CERV-K60        1918 AGTACTTCCCATGTTAAAAAACATTTATTGTCTTGTTTTGCTGTAATGGGAGTTCCAGAAAAAATCAAAACTGACAATGGACCAGGATATTGTAGTAAAGCTTTCCAAAAATTCTTAAGT 2037
CERV-K102       1918 AGTACTTCCCATGTTAAAAAACATTTATTGTCTTGTTTTGCTGTAATGGGAGTTCCAGAAAAAATCAAAACTGACAATGGACCAAGATATTGTAGTAAAGCTTTCCAAAAATTCTTAAGT 2037
HERV-K104       1918 AGTACTTCCCATGTTAAAAAACATTTATTGTCTTGTTTTGCTGTAATGGGAGTTCCAGAAAAAATCAAAACTGACAATGGACCAGGATATTGTAGTAAAGCTTTCCAAAAATTCTTAAGT 2037
HERV-K50D       1918 AGTACTTCCCATGTTAAAAAACATTTATTGTCTTGTTTTGCTGTAATGGGAGTTCCAGAAAAAATCAAAACTGACAATGGACCAGGATATTGTAGTAAAGCTTTCCAAAAATTCTTAAGT 2037
HERV-K50B       1918 AGTACTTCCCATGTTAAAAAACATTTATTGTCTTGTTTTGCTGTAATGGGAGTTCCAGAAAAAATCAAAACTGACAATGGACCAGGATATTGTAGTAAAGCTTTCCAAAAATTCTTAAGT 2037
HERV-K106       1918 AGTACTTCCCATGTTAAAAAACATTTATTGTTTTG-----CTATAATGGGAGTTCCAGAAAAAATCAAAACTGGCAATGGACCAGGATATTGTAGTAAAGCTTTCCAAAAATTCTTAAGT 2032
HERV-K101       1917 AGTACTTCCCATGTTAAAAAACATTTATTGTCTTGTTTTGCTGTAATGGGAGTTCCAGAAAAAATCAAAACTGACAATGGACCAGGATATTGTAGTAAAGCTTTCCAAAAATTCTTAAGT 2036
HERV-K103       1918 AGTACTTCCCATGTTAAAAAACATTTATTGTCTTGTTTTGCTGTAATGGGAGTTCCAGAAAAAATCAAAACTGACAATGGACCAGGATATTGTAGTAAAGCTTTCCAAAAATTCTTAAGT 2037
CERV-K100       1918 AGTACTTCCCATGTTAAAAAACATTTATTGTCTTGTTTTGCTGTAATGGGAGTTCCAGAAAAAATCAAAACTGACAATGGACCAGGATATTGTAGTAAAGCTTTCCAAAAATTCTTAAGT 2037
HERV-K10        1918 AGTACTTCCCATGTTAAAAAACATTTATTGTCTTGTTTTGCTGTAATGGGAGTTCCAGAAAAAATCAAAACTGACAATGGACCAGGATATTGTAGTAAAGCTTTCCAAAAATTCTTAAGT 2037
HERV-K108       1918 AGTACTTCCCATGTTAAAAAACATTTATTGTCTTGTTTTGCTGTAATGGGAGTTCCAGAAAAAATCAAAACTGACAATGGACCAGGATATTGTAGTAAAGCTTTCCAAAAATTCTTAAGT 2037
HERV-K109       1887 AGTACTTCCCATGTTAAAAAACATTTATTGTCTTGTTTTGCTGTAATGGGAGTTCCAGAAAAAATCAAAACTGACAATGGACCAGGATATTGTAGTAAAGCTTTCCAAAAATTCTTAAGT 2006
HERV-K113       1918 AGTACTTCCCATGTTAAAAAACATTTATTGTCTTGTTTTGCTGTAATGGGAGTTCCAGAAAAAATCAAAACTGACAATGGACCAGGATATTGTAGTAAAGCTTTCCAAAAATTCTTAAGT 2037
HERV-K36        1912 AGTACTTCCCATGTTAAAAAACACTTATTGTCTTGTTTTGCTGTAATGGGAGTTCCAGAAAAAATCAAAACTGACAATGGACCAGGATATTGTAGTAAAGCTTTCCAAAAATTCTTAAGT 2031
HERV-K115       1918 AGTACTTCCCATGTTAAAAAACATTTATTGTCTTGTTTTGCTGTAATGGGAGTTCCAGAAAAAATCAAAACTGACAATGGACCAGGATATTGTAGTAAAGCTTTCCAAAAATTCTTAAGT 2037
HERV-K102       1918 AGTACTTCCCATGTTAAAAAACATTTATTGTCTTGTTTTGCTGTAATGGGAGTTCCAGAAAAAATCAAAACTGACAATGGACCAGGATATTGTAGTAAAGCTTTCCAAAAATTCTTAAGT 2037
HERV-K50A       1918 AGTACTTCCCATGTTAAAAAACATTTATTGTCTTGTTTTGCTGTAATGGGAGTTCCAGAAAAAATCAAAACTGACAATGGACCAGGATATTGTAGTAAAGCTTTCCAAAAATTCTTAAGT 2037
                     * ***************** *** * * * * ***      ** * ******************** *****     ***** *** * ** ********  * ** ************* *

CERV-KOLD35587  2037 CAGTGGAAAATTACACATACAACAGGAATCCCTTATAATTCCCAAGGACAGGTCATAATTGAAAAAACTGATAGAACACTCAAAGCTCAATTGGTTAAACAAAAAAAGGAAAAAGACAGT 2156
HERV-KOLD35587  2037 CAGTGGAAAATTACACATACAACAGGAATCCCTTATAATTCCCAAGGACAGGCCATAATTGAAAAAACTGATAGAACACTCAAAGCTCAATTGGTTAAACAAAAAAAGGAAAAAGACAGT 2156
SERV-K1 chr.11  2038 CAGGGGAAAATTACACATACAACAGGAATTCCTTATAATTCCCAAGGACAGGCCATAGTTGAAAGAACTAATAGAACACTCAAAACTCAGTTAATTAAACAAAAAGAAGGGGGAGACAGT 2157
Guereza         2038 CAGTGGAATATTACACATACAACAGGAATTCCTTATAATTCCCAAGGACAGGCCATAGTTGAAAGAACTAATAGAACACTCAAAACTCAGTTAGTTAAACAAAAAGAAGGGGGAGTCAGT 2157
SERV-K1 chr.12  2038 CAGTGGAATATTACACATACAACAGGAATTCCTTATAATTCCCAAGGACAGGCCATAGTTGAAAGAACTAATAGAACACTCAAAACTCAATTAATTAAACAAAAAAAAGGGGGAGACAGT 2157
Grivet          2038 CAGTGGAATATTACACATACAACAGGAATTCCTTATAATTCCCAAGGACAGGCCATAGTTGAAAGAACTAATAGAACACTCAAAACTCAGTTAATTAAACAAAAAAAAGGGGGAGACAGT 2157
SERV-K1 chr.5   2031 CAGTGGAAAATTACACACACAACAGGAATTCCTTCTAATTCCCAAGGACAGGCCAT-GTTGAAAGAACTAATAGAACACTCAAAACTCAGTTAATTAAACAAAAAGAAGGGGGAGACAGT 2149
CERV-K60        2038 CAGTGGAAAATTTCACATACAACAGGAATTCCTTATAATTCCCAAGGACAGGCCATAGTTGAAAGAACTAACAGAACACTCAAAACTCAGTTA---ACACAAAAAGAAGGGGGAGACAGT 2154
CERV-K102       2038 CAGTGGAAAATTTCACATACAACAGGAATTCCTTATAATTCCCAAGGACAGGCCATAGTTGAAAGAACTAATAGAACACTCAAAACTCAATTAGTTAAACAAAAAGAAGGGGGAGACAGT 2157
HERV-K104       2038 CAGTGGAAAATTTCACATACAACAGGAATTCCTTATAATTCCCAAGGACAGGCCATAGTTGAAAGAACTAATAGAACACTCAACACTC----AGTTAAACAAAAAGAAGGGGGAGACAGT 2153
HERV-K50D       2038 CAGTGGAAAATTTCACATACAACAGGAATTCCTTATAATTCCCAAGGACAGGCCATAGTTGAAAGAACTAATAGAACACTCAACACTC----AGTTAAACAAAAAGAAGGGGGAGACAGT 2153
HERV-K50B       2038 CAGTGGAAAATTTCACATACAACAGGAATTCCTTATAATTCCCAAGGACAGGCCATAGTTGAAAGAACTAATAGAACACTCAAAACTCAATTAGTTAAACAAAAAGAAGGGGGAGACAGT 2157
HERV-K106       2033 CAGTGGAAAATTTCACATACAACAGGAATTCCTTATAATTCCCAAGGACAGGCCATAGTTGAAAGAACTAATAGAACACTCAAAACTCAATTAGTTAAACAAAAAGAAGGGGGAGACAGT 2152
HERV-K101       2037 CAGTGGAAAATTTCACATACGACAGGAATTCCTTATAATTCCCAAGGACAGGCCATAGTTGAAAGAACTAATAGAACACTCAAAACTCAATTAGTTAAACAAAAAGAAGGGGGAGACAGT 2156
HERV-K103       2038 CAGTGGAAAATTTCACATACAACAGGAATTCCTTATAATTCCCAAGGACAGGCCATAGTTGAAAGAACTAATAGAACACTCAAAACTCAATTAGTTAAACAAAAAGAAGGGGGAGACAGT 2157
CERV-K100       2038 CAGTGGAAAATTTCACATACAACAGGAATTCCTTATAATTCCCAAGGACAGGCCATAGTTGAAAGAACTAATAGAACACTCAAAACTCAATTAGTTAAACAAAAAGAAGGGGGAGACAGT 2157
HERV-K10        2038 CAGTGGAAAATTTCACATACAACAGGAATTCCTTATAATTCCCAAGGACAGGCCATAGTTGAAAGAACTAATAGAACACTCAAAACTCAATTAGTTAAACAAAAAGAAGGGGGAGACAGT 2157
HERV-K108       2038 CAGTGGAAAATTTCACATACAACAGGAATTCCTTATAATTCCCAAGGACAGGCCATAGTTGAAAGAACTAATAGAACACTCAAAACTCAATTAGTTAAACAAAAAGAAGGGGGAGACAGT 2157
HERV-K109       2007 CAGTGGAAAATTTCACATACAACAGGAATTCCTTATAATTCCCAAGGACAGGCCATAGTTGAAAGAACTAATAGAACACTCAAAACTCAATTAGTTAAACAAAAAGAAGGGGGAGACAGT 2126
HERV-K113       2038 CAGTGGAAAATTTCACATACAACAGGAATTCCTTATAATTCCCAAGGACAGGCCATAGTTGAAAGAACTAATAGAACACTCAAAACTCAATTAGTTAAACAAAAAGAAGGGGGAGACAGT 2157
HERV-K36        2032 CAGTGGAAAATTTCACATACAACAGGAATTCCTTATAATTCCCAAGGACAGGCCATAGTTGAAAGAACTAATAGAACACTCAAAACTCAATTAGTTAAACAAAAAGAAGGGGGAGACAGT 2151
HERV-K115       2038 CAGTGGAAAATTTCACATACAACAGGAATTCCTTATAATTCCCAAGGACAGGCCATAGTTGAAAGAACTAATAGAACACTCAAAACTCAATTAGTTAAACAAAAAGAAGGGGGAGACAGT 2157
HERV-K102       2038 CAGTGGAAAATTTCACGTACAACAGGAATTCCTTATAATTCCCAAGGACAGGCCATAGTTGAAAGAACTAATAGAACACTCAAAACTCAATTAGTTAAACAAAAAGAAGGGGGAGACAGT 2157
HERV-K50A       2038 CAGTGGAAAATTTCACATACAACAGGAATTCCTTATAATTCCCAAGGACAGGCCATAGTTGAAAGAACTAATAGAACACTCAAAACTCAATTAGTTAAACAAAAAGAAGGGGGAGACAGT 2157
                     *** **** *** ***  ** ******** **** **************** ***  ****** **** * ***********  ***        * ******* * *     ** ****

CERV-KOLD35587  2157 AAGGAGTATAACACTCCCCAGATGCAACTTAATCTAGCACTCTATACTTTAAATTTTTTAAACATTTATAGAAATCAAACCACTACTTCTCCAGAACAACATTTTACTGGTAAAAAGAAC 2276
HERV-KOLD35587  2157 AAGGAGTATAACACTCCCCAGATGCAACTTAATCTAGCACTCTATACTTTAAATTTTTTAAACATTTATAGAAATCAAACCACTACTTCTGCAGAACAACATTTTACTGGTAAAAAGAAT 2276
SERV-K1 chr.11  2158 AAGGAGTGTACCACTCCTCAGATGCAGCTTAATCTAGCACTCTATACTTTAAAATTTTTAAACATTTATAGAAATCAGACTACAACTTCTGCAGAACAACATCTTACTGGTAAAAAGAAC 2277
Guereza         2158 AAGGAGTGTACCTCTCCTCAGATGCAGCTTAATCTAGCACTCTATACTTTAAATTTTTTAAACATTTATAGAAATCAGACTACCACTTCTGCAGAACAACATCTTACTGGTAAAAAGAAC 2277
SERV-K1 chr.12  2158 AAGGAGTATACCACTCCTCAGATGCAGCTTAATCTAGCACTCTATACTTTAAATTTTTTAAACATTTATAGAAATCAGACTACTACTTCTGCAGAACAACATCTTACTGGTAAAAAGAAC 2277
Grivet          2158 AAGGAGTGTACCACTCCTCAGATGCAGCTTAATCTAGCACTCTATACTTTAAATTTTTTAAACATTTATAGAAATCAGACTACTACTTCTGCAGAACAACATCTTACTGGTAAAAAGAAC 2277
SERV-K1 chr.5   2150 AAGGAGTGTACCACTCCTCAGATGCAACTTAATCTAGCACTCTATACTTTATATGTTTTAAACATTTATAGAAGTCAGACTAGTACTTCTGTAGAACAACATCTTACTGGTAAAAAGAAC 2269
CERV-K60        2155 AAGGAGTGTACCACTCCTCAGATGCAACTTAATCTAGCACTCTATACTTTAAATTTTTTAAACATTTATAGAAATCAGACTACTACTTCTGCAG---AACATCTTACTGGTAAAAAGAAC 2271
CERV-K102       2158 AAGGAGTGTACCACTCCTCAGATGCAACTTAATCTAGCACTCTATACTTTAAATTTTTAAAACATTTATAGAAATCAGACTACTACTTCTGTAGAACAACATCTTTCTGGTAAAAAGAAC 2277
HERV-K104       2154 AAGGAGTGTACCACTCCTCAGATGCAACTTAATCTAGCACTCTATACTTTAAATTTTTTAAACATTTATAGAAATCAGACTACTACTTCTGCAGAACAACATCTTACTGGTTAAAAGAAC 2273
HERV-K50D       2154 AAGGAGTGTACCACTCCTCAGATGCAACTTAATCTAGCACTCTATACTTTAAATTTTTTAAACATTTATAGAAATCAGACTACTACTTCTGCAGAACAACATCTTACTGGTTAAAAGAAC 2273
HERV-K50B       2158 AAGGAGTGTACCACTCCTCAGATGCAACTTAATCTAGCACTCTATACTTTAAATTTTTTAAACATTTATAGAAATCAGACTACTACTTCTGCAGAACAACATCTTACTGGTAAAAAGAAC 2277
HERV-K106       2153 AAGGAGTGTACCACTCCTCAGATGCAACTTAATCTAGCACTCTATACTTTAAATTTTTTAAACATTTATAGAAATCAGACTACTACTTCTGCAGAACAACATCTTACTGGTAAAAAGAAC 2272
HERV-K101       2157 AAGGAGTGTACCACTCCTCAGATGCAACTTAATCTAGCACTCTATACTTTAAATTTTTTAAACATTTATAGAAATCAGACTACTACTTCTGCAGAACAACATCTTACTGGTAAAAAGAAC 2276
HERV-K103       2158 AAGGAGTGTACCACTCCTCAGATGCAACTTAATCTAGCACTCTATACTTTAAATTTTTTAAACATTTATAGAAATCAGACTACTACTTCTGCAGAACAACATCTTACTGGTAAAAAGAAC 2277
CERV-K100       2158 AAGGAGTGTACCACTCCTCAGATGCAACTTAATCTAGCACTCTATACTTTAAATTTTTTAAACATTTATAGAAATCAGACTACTACTTCTGCAGAACAACATCTTACTGGTAAAAAGAAC 2277
HERV-K10        2158 AAGGAGTGTACCACTCCTCAGATGCAACTTAATCTAGCACTCTATACTTTAAATTTTTTAAACATTTATAGAAATCAGACTACTACTTCTGCAGAACAACATCTTACTGGTAAAAAGAAC 2277
HERV-K108       2158 AAGGAGTGTACCACTCCTCAGATGCAACTTAATCTAGCACTCTATACTTTAAATTTTTTAAACATTTATAGAAATCAGACTACTACTTCTGCAGAACAACATCTTACTGGTAAAAAGAAC 2277
HERV-K109       2127 AAGGAGTGTACCACTCCTCAGATGCAACTTAATCTAGCACTCTATACTTTAAATTTTTTAAACATTTATAGAAATCAGACTACTACTTCTGCAGAACAACATCTTACTGGTAAAAAGAAC 2246
HERV-K113       2158 AAGGAGTGTACCACTCCTCAGATGCAACTTAATCTAGCACCCTATACTTTAAATTTTTTAAACATTTATAGAAATCAGACTACTACTTCTGCAGAACAACATCTTACTGGTAAAAAGAAC 2277
HERV-K36        2152 AAGGAGTGTACCACTCCTCAGATGCAACTTAATCTAGCACTCTATACTTTAAATTTTTTAAACATTTATAGAAATCAGACTACTACTTCTGCAGAACAACATCTTACTGGTAAAAAGAAC 2271
HERV-K115       2158 AAGGAGTGTACCACTCCTCAGATGCAACTTAATCTAGCACTCTATACTTTAAATTTTTTAAACATTTATAGAAATCAGACTACTACTTCTGCAGAACAACATCTTACTGGTAAAAAGAAC 2277
HERV-K102       2158 AAGGAGTGTACCACTCCTCAGATGCAACTTAATCTAGCACTCTATACTTTAAATTTTTTAAACATTTATAGAAATCAGACTACTACTTCTGCAGAACAACATCTTACTGGTAAAAAGAAC 2277
HERV-K50A       2158 AAGGAGTGTACCACTCCTCAGATGCAACTTAATCTAGCACTCTATACTTTAAATTTTTTAAACATTTATAGAAATCAGACTACTACTTCTGCAGAACAACATCTTACTGGTAAAAAGAAC 2277
                     ******* ** * **** ******** ************* ********** *  *** ************** *** ** *  ******   **    ***** ** ***** *******

CERV-KOLD35587  2277 AGCCCACATGAAGGAAAACTG 2297
HERV-KOLD35587  2277 AGCCCACATGAAGGAAAACTG 2297
SERV-K1 chr.11  2278 AGTCCACATGAAGGAAAACTG 2298
Guereza         2278 AGCCCACATGAAGGAAAACTG 2298
SERV-K1 chr.12  2278 AGTCCACATGAAGGAAAACTG 2298
Grivet          2278 AGTCCACATGAAGGAAAACTG 2298
SERV-K1 chr.5   2270 AGCCCACATGAAGGAAAACTG 2290
CERV-K60        2272 AGCCCACATGAAGGAAAACTA 2292
CERV-K102       2278 AGCCCACATGAAGGAAAACTA 2298
HERV-K104       2274 AGCCCACATGAAGGAAAACTA 2294
HERV-K50D       2274 AGCCCACATGAAGGAAAACTA 2294
HERV-K50B       2278 AGCCCACATGAAGGAAAACTA 2298
HERV-K106       2273 AGCCCACATGAAGGAAAACTA 2293
HERV-K101       2277 AGCCCACATGAAGGAAAACTA 2297
HERV-K103       2278 AGCCCACATGAAGGAAAACTA 2298
CERV-K100       2278 AGCCCACATGAAGGAAAACTA 2298
HERV-K10        2278 AGCCCACATGAAGGAAAACTA 2298
HERV-K108       2278 AGCCCACATGAAGGAAAACTA 2298
HERV-K109       2247 AGCCCACATGAAGGAAAACTA 2267
HERV-K113       2278 AGCCCACATGAAGGAAAACTA 2298
HERV-K36        2272 AGCCCACATGAAGGAAAACTA 2292
HERV-K115       2278 AGCCCACATGAAGGAAAACTA 2298
HERV-K102       2278 AGCCCACATGAAGGAAAACTA 2298
HERV-K50A       2278 AGCCCACATGAAGGAAAACTA 2298
                     ** *****************
```
