# Supplementary material for: Identification and spontaneous immune targeting of an endogenous retrovirus K envelope protein in the Indian rhesus macaque model of human disease
Source: Retrovirology. 2016 Jan 15;13:6. doi: 10.1186/s12977-016-0238-0 (PMC4714462; doi:10.1186/s12977-016-0238-0)

Figure S4. ELISPOT titration of SERV-K1 Env-specific T cell responses. (a) Titration of SERV-K1 Env LL15-specific CD4+ T cell response in r02120 (1 week prior to SIV infection) using decreasing concentrations of peptide. Influenza nucleoprotein 15-mer MQ15 is included as a negative control. **(b)** Titration of SERV-K1 Env PA8-specific CD8+ T cell response in r02120 (20 weeks post-SIV infection) using decreasing concentrations of peptide.

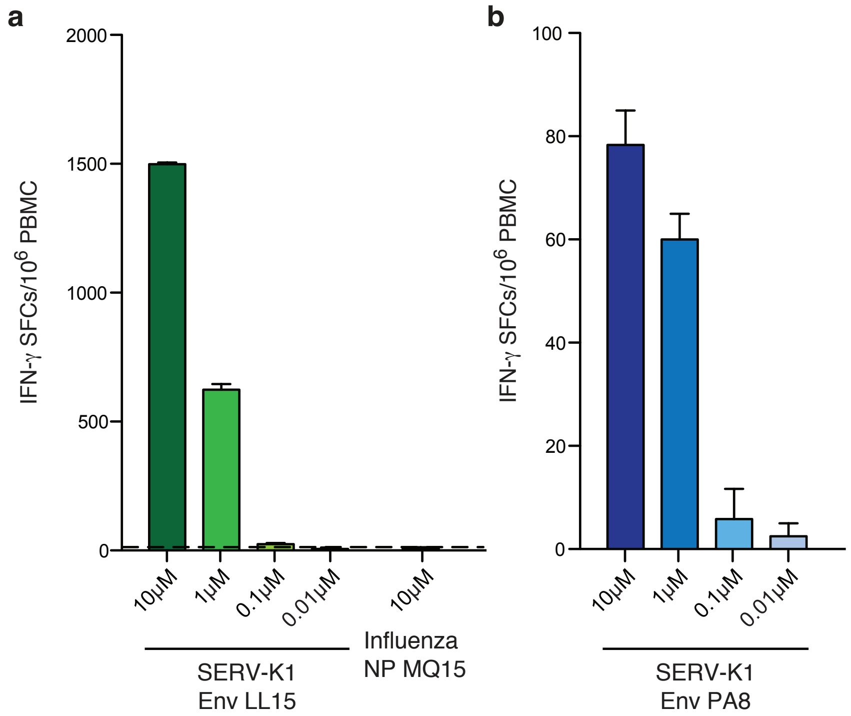

Supplement: Supplementary file 4 — 10.1186/s12977-016-0238-0 Figure S4. ELISPOT titration of SERV-K1 Env-specific T cell responses. a Titration of SERV-K1 Env LL15-specific CD4+ T cell response in r02120 (1 week prior to SIV infection) using decreasing concentrations of peptide. Influenza nucleoprotein 15-mer MQ15 is included as a negative control. b Titration of SERV-K1 Env PA8-specific CD8+ T cell response in r02120 (20 weeks post-SIV infection) using decreasing concentrations of peptide. [file 12977_2016_238_MOESM4_ESM.pdf]
