# Supplementary material for: Identification and spontaneous immune targeting of an endogenous retrovirus K envelope protein in the Indian rhesus macaque model of human disease
Source: Retrovirology. 2016 Jan 15;13:6. doi: 10.1186/s12977-016-0238-0 (PMC4714462; doi:10.1186/s12977-016-0238-0)
Supplement: Supplementary file 5 — 10.1186/s12977-016-0238-0 Figure S5. Alignments of SERV-K1 Env T cell epitopes to SIVmac239 Env peptides. a Amino acid alignment of r02120 CD4+ T cell epitope SERV-K1 Env LL15 to SIVmac239 Env LL15. This is the only SIVmac239 Env peptide with 10 or less mismatches to SERV-K1 Env LL15. b Amino acid alignment of r02120 CD8+ T cell epitope SERV-K1 Env PA8 to SIVmac239 Env PC8 and AQ8. These are the only SIVmac239 Env peptides with 5 or less mismatches to SERV-K1 Env PA8. For both a and b, matches to the SERV-K1 Env peptide are shown as dashes. [file 12977_2016_238_MOESM5_ESM.pdf]

Figure S5. Alignments of SERV-K1 Env T cell epitopes to SIVmac239 Env peptides. (a) Amino acid alignment of r02120 CD4+ T cell epitope SERV-K1 Env LL15 to SIVmac239 Env LL15. This is the only SIVmac239 Env peptide with 10 or less mismatches to SERV-K1 Env LL15. **(b)** Amino acid alignment of r02120 CD8+ T cell epitope SERV-K1 Env PA8 to SIVmac239 Env PC8 and AQ8. These are the only SIVmac239 Env peptides with 5 or less mismatches to SERV-K1 Env PA8. For both (a) and (b), matches to the SERV-K1 Env peptide are shown as dashes.

a

```
   SERV-K1 Env 526-540 LRQTVIWMGDRLMSL
 SIVmac239 Env 703-717 -LRI--YIVQM-AK-
```

b

```
   SERV-K1 Env 109-115 PFPPLIRA
 SIVmac239 Env 236-243 -GYA-L-C
 SIVmac239 Env 865-872 AI-RR--Q
```
